# Routine Laboratory Tests Predict 72‐h Fatality in Patients With D‐Dimer Levels ≥ 2 μg/mL: A Retrospective Cohort Study Comparing Statistical and Machine Learning Models

**DOI:** 10.1002/jcla.70091

**Published:** 2025-09-03

**Authors:** Shuma Hayashi, Ryoko Hayashi, Kayoko Nakamura, Kai Saito, Hidenori Sanayama, Takahiko Fukuchi, Tamami Watanabe, Kiyoka Omoto, Hitoshi Sugawara

**Affiliations:** ^1^ Division of General Medicine, Department of Comprehensive Medicine 1 Jichi Medical University, Saitama Medical Center Saitama Japan; ^2^ Division of General Medicine Center for Community Medicine, Jichi Medical University School of Medicine Shimotsuke Japan; ^3^ Department of Laboratory Medicine Jichi Medical University, Saitama Medical Center Saitama Japan

**Keywords:** 72‐h fatality, gradient boosting decision tree, machine learning, multivariate logistic regression analysis, routine laboratory test, SHapley additive exPlanation

## Abstract

**Background:**

Despite the high prognostic value of D‐dimer in various clinical conditions, limited research has addressed short‐term fatality prediction across disease categories. This study aimed to develop and compare models predicting 72‐h fatality in patients with D‐dimer levels ≥ 2 μg/mL, using laboratory variables. This timeframe was chosen based on its clinical relevance for early triage and intervention across multiple acute conditions.

**Methods:**

We retrospectively analyzed data from 5158 patients (241 deaths within 72 h). The primary outcome was 72‐h fatality; predictors included age, sex, and 40 routine hematologic, biochemical, and coagulation tests. Traditional multivariate logistic regression analysis (MLRA) was compared with four machine learning (ML) models: Prediction One, LightGBM, XGBoost, and CatBoost. External validation was performed using a separate dataset of 5550 patients (309 deaths). D‐dimer levels were recorded in any clinical setting despite limited patient medical information.

**Results:**

The 72‐h fatality rate increased with increasing D‐dimer levels (overall 4.67%). Major causes of death were intracranial disease (24.9%), malignancy (17.0%), and sepsis (8.3%). MLRA identified five key predictors: advanced age, low total protein and cholesterol levels, and elevated aspartate aminotransferase and D‐dimer levels. Its performance (AUC 0.829, 95% CI 0.768–0.888; sensitivity 0.762; specificity 0.809) was exceeded by LightGBM (AUC 0.987; sensitivity 0.987; specificity 0.911), which outperformed Prediction One (0.814), XGBoost (0.981), and CatBoost (0.937).

**Conclusion:**

ML models, particularly LightGBM, effectively identify high‐risk patients using routine laboratory tests. The model enables timely decision‐making and early risk stratification in patients with high D‐dimer values, even when clinical information is limited.

## Graphical abstract

This study compares multivariate logistic regression (MLRA) and four machine learning models in predicting 72‐h fatality in patients with D‐dimer levels ≥ 2 μg/mL. MLRA identified five significant factors: advanced age, low total protein and cholesterol levels, and high aspartate aminotransferase and D‐dimer levels. LightGBM outperformed all other models, achieving the highest predictive accuracy and calibration. These findings highlight the potential of machine learning in improving clinical management of high‐risk patients with elevated D‐dimer levels.

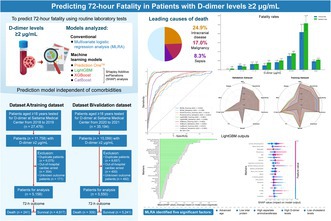

## 1 Introduction

D‐dimer is a key marker used to assess coagulation and fibrinolysis processes. D‐dimer results are typically reported as either D‐dimer units (DDUs; molecular weight, 195 kDa) or fibrinogen equivalent units (FEUs; molecular weight, 340 kDa), with a 1.75‐fold difference between the FEUs and DDUs [1]. The commonly used reference ranges are ≤ 0.5 μg/mL FEU [2] or ≤ 1.0 μg/mL DDU [3].

Negative D‐dimer results strongly suggest the absence of acute pulmonary embolism and deep vein thrombosis (DVT) [4, 5]. Research has indicated a 30% fatality rate within 72 h for patients with DDU > 300 μg/mL [6]. Elevated D‐dimer levels are associated with medical emergencies such as aortic aneurysm rupture [7], pulmonary embolism, DVT, pneumonia, sepsis, cancer [8, 9], and disseminated intravascular coagulation [1], often leading to poor outcomes [8]. Higher levels (DDU ≥ 2 μg/mL) have been associated with advanced outcomes in colorectal cancer [9], coronavirus disease 2019 (COVID‐19) [10, 11, 12, 13], and amyloidosis [14].

In real‐world clinical settings, physicians frequently make urgent decisions at first medical contact without access to complete clinical information, including medication history such as anticoagulant use or a prior history of malignancy. In this context, models based solely on routinely available laboratory data, including the D‐dimer levels, offer timely prognostic support.

The 72‐h timeframe is clinically critical in emergency and intensive care, because rapid deterioration is common in conditions such as trauma [15], sepsis [16, 17], stroke [18], organ failure [19, 20], and metabolic crises [21, 22]. This period also aligns with the “Golden 72 Hours” principle of disaster medicine [23] and corresponds to the optimal window for initiating time‐sensitive therapies, including antivirals for herpes zoster [24] and COVID‐19 [25].

Although the prognostic value of D‐dimer is well documented for specific diseases, its cross‐disease predictive utility within this timeframe remains insufficiently studied. Such a model may assist risk stratification in the absence of detailed patient information.

Multivariate logistic regression analysis (MLRA) clarifies relationships between variables but assumes linearity and is sensitive to missing data. Machine learning (ML) provides high predictive accuracy and models complex relationships but is often criticized for its lack of interpretability and dependence on data quality and quantity. Comparison of MLRA and ML improves prediction accuracy and interpretability [26].

This study aimed to develop and compare predictive models for 72‐h fatality in patients with DDU ≥ 2 μg/mL across all disease categories, using only objective laboratory values. We evaluated traditional MLRA alongside four ML methods, including Prediction One (Sony Network Communications Inc., Tokyo, Japan; https://predictionone.sony.biz/), Light Gradient Boosting Machine (LightGBM), Extreme Gradient Boosting (XGBoost), and Categorical Boosting (CatBoost). By refining these models, we sought to enable early identification of high‐risk patients, support timely clinical decisions, and improve outcomes through better prioritization of care.

## 2 Materials and Methods

### 2.1 Study Design, Setting, Participant Selection, and Data Collection

This single‐center, retrospective inception cohort study was conducted at Jichi Medical University, Saitama Medical Center and included adult patients aged ≥ 18 years who underwent D‐dimer testing. The training cohort (Dataset‐A) consisted of 27,479 patients tested between 2018 and 2019, of whom 11,758 (42.8%) had D‐dimer levels of ≥ 2 μg/mL. After applying exclusion criteria—out‐of‐hospital cardiac arrest, indeterminate outcomes due to transfer, and duplicate D‐dimer measurements, where only the highest value was retained—the final training dataset included 5158 patients.

D‐dimer measurements were included irrespective of clinical setting (outpatient, emergency, or inpatient) to reflect the model's intended application in early‐phase decision‐making across diverse clinical environments. As a retrospective study, the timing and clinical context of D‐dimer testing, such as proximity to symptom onset or anticoagulant administration, were not controlled. This approach captures the heterogeneity characteristic of real‐world medical care.

For external validation, a separate cohort (Dataset‐B) included 35,194 patients tested between 2020 and 2021. Among them, 15,099 patients (42.9%) had D‐dimer levels ≥ 2 μg/mL. Using the same exclusion criteria as for Dataset‐A, the final validation dataset included 5550 patients.

The selection process and exclusion criteria applied to both cohorts are summarized in Figure S1, which outlines the steps leading to the final datasets used for model development and validation.

### 2.2 Sample Size Estimation

Sample size was computed using G*Power [27], with assumptions based on a previous study [6]. The initial sample size was 652, but it was expanded to 5158 for higher statistical power. Detailed calculations are shown in Text S1.

### 2.3 Routine Laboratory Tests

Routine hematologic, biochemical, and coagulation tests were performed. Instruments and procedures are detailed in Text S2.

### 2.4 Primary Outcome and Endpoints

The primary outcome was defined as all‐cause fatality within 72 h after D‐dimer testing. Cases were patients with DDU ≥ 2 μg/mL who died within 72 h, either in the hospital or post‐admission. Controls were patients with DDU ≥ 2 μg/mL who survived. The index date of the inception cohort was defined as the time when the D‐dimer test was performed.

### 2.5 Issue of Interest

The study analyzed variables associated with 72‐h fatality, including age; sex; 40 routine hematologic, biochemical, and coagulation tests; and expected causes of death. A comprehensive list of variables is provided in Text S3.

### 2.6 Statistical Analysis

#### 2.6.1 Descriptive Statistics and the Estimated Cause of Death

Cross tabulations of survival and death outcomes were performed for the training dataset. Differences were tested using Fisher's exact or Pearson's chi‐square test for nominal variables and Mann–Whitney *U* test for continuous variables.

#### 2.6.2 Seventy‐Two‐Hour Fatality Rates Classified by D‐Dimer Concentration

We analyzed the 72‐h fatality rate by D‐dimer concentration for both datasets; applying chi‐squared tests across each concentration range and overall.

#### 2.6.3 Estimated Causes of Death

Causes of death in the training dataset were categorized using the International Classification of Diseases‐11 coding tool [28].

#### 2.6.4 Association Analysis Using Logistic Regression Analysis

All continuous valuables were assessed for normality and transformed where necessary using the Box‐Cox method [29]. Univariate logistic regression identified factors associated with 72‐h fatality. Significant factors were further analyzed using receiver operating characteristic curves and the area under the curve (AUC). Multivariate analysis adjusted for age and sex, calculating adjusted odds ratios (ORs) with 95% confidence intervals (CIs). Further details are provided in Text S4.

### 2.7 Model 1 Development Using MLRA

Model 1 was developed using MLRA following the TRIPOD+AI statement [30]. The selection of variables ensured at least 10 events (deaths) per variable. The final model was chosen based on achieving the highest AUC and the lowest Akaike information criterion, excluding variables with multicollinearity (variance inflation factor ≥ 5). Further details are provided in Text S5.

To assess whether malignancy modified the association between D‐dimer levels and 72‐h fatality, an exploratory analysis was conducted using Firth's penalized logistic regression [31] in R‐4.5.1 (June 2025), implemented on the Google Colaboratory environment. Given that malignancy was the second most common cause of death in the training cohort and is pathophysiologically associated with elevated D‐dimer levels, we defined a binary malignancy variable (1 = death due to malignancy, 0 = otherwise) and included its interaction with *log*‐transformed D‐dimer as a multiplicative term in the model. This analysis was limited to the training cohort, where cause‐of‐death data were complete. The full R code and output are provided in the Supporting Information Program Codes.

### 2.8 Model 2 Development Using Prediction One

Model 2 was built using Prediction One software, which automatically generates models and assesses variable importance using permutation methods. Performance metrics such as AUC, F‐score, and recall are shown in Text S6.

### 2.9 ML Prediction Models Using Gradient Boosting Decision Trees (GBDTs)

Three models were developed using LightGBM (Model 3), XGBoost (Model 4), and CatBoost (Model 5). The hyperparameters for each model are listed in Text S7. Feature importance was assessed using SHapley Additive exPlanations (SHAP) values with bar plots, beeswarm summary plots, and dependence plots.

### 2.10 Bootstrap Internal Validations

Internal validation for Model 1 and Models 3–5 was performed using 1000 bootstrap resamples, calculating the mean AUC, sensitivity, specificity, and other metrics. Details are provided in Text S8 [32].

### 2.11 External Validation

Models were externally validated using the validation dataset; comparing metrics with the training dataset to check for overfitting. Details are provided in Text S9.

### 2.12 Calibration Plots

Calibration plots were generated for all models to assess predicted probabilities against actual outcomes in both datasets. Additionally, the intercept, slope, R‐squared, *p*‐value, and standard error from the calibration lines were compared to evaluate the calibration performance of each model.

### 2.13 Statistical Software

Statistical analyses were performed using StatFlex (Model 1), Prediction One (Model 2), and Python (Models 3–5). Analyses using Python were conducted within the Google Colaboratory environment. Detailed software versions and code are provided in Text S10 and Supporting Information Program Codes. A *p*‐value < 0.05 was considered statistically significant.

### 2.14 Handling of Missing Data

The handling of missing data depended on the modeling approach. For the MLRA, complete case analysis (listwise deletion) was applied without imputation. By contrast, all evaluated ML models could process missing data internally, eliminating the need for prior imputation or deletion.

## 3 Results

### 3.1 Seventy‐Two‐Hour Fatality Rates Stratified by D‐Dimer Concentration and the Cause of Death

**FIGURE 1:**
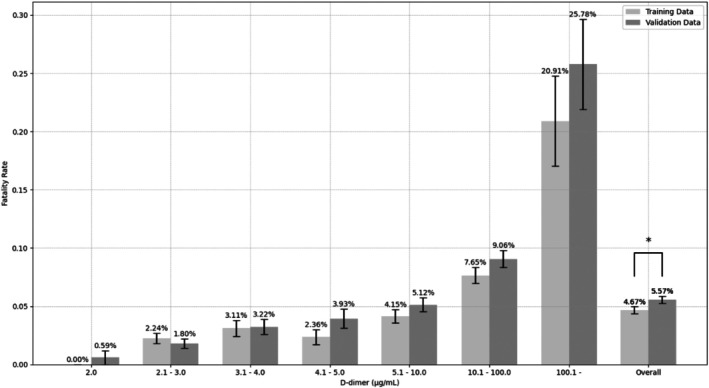
Seventy‐two‐hour fatality rates stratified by D‐dimer levels. The 72‐h fatality rates are stratified by D‐dimer levels and demonstrate a concentration‐dependent increase in fatality. The overall fatality rates are 4.67% (241/5158) and 5.57% (309/5550) in the training and validation datasets, respectively, with a statistically significant difference (*χ* ^2^ = 4.215, *p* = 0.040). Fatality rates range from 0% to 20.9% and from 0.59% to 25.8% in the training and validation datasets, respectively, depending on the D‐dimer level. Error bars represent standard errors of the mean.

Fatality rates increased in a concentration‐dependent manner across D‐dimer levels. At concentrations > 4.1 μg/mL, rates were consistently higher in Dataset B, but differences were not statistically significant. The overall fatality rates were 4.67% (241/5158) in Dataset A and 5.57% (309/5550) in Dataset B, significantly different (*p* = 0.040) (Figure 1). Causes of death in the training group are summarized in Table 1.

**TABLE 1:** Causes of death among the training group.

Expected causes of death (*n* = 241)	Number	%
Intracranial diseases	60	24.9
Any malignancy	41	17.0
Sepsis	20	8.3
Coronary artery disease	19	7.9
Aortic disease	18	7.4
Pneumonia	18	7.4
Gastrointestinal disorders (perforation or obstruction or bleeding)	15	6.2
Other infections	8	3.3
Arrhythmia	5	2.1
Heart failure	5	2.1
Pulmonary thromboembolism	3	1.2
Others	29	12.0

### 3.2 Participant Demographics

Tables S1 and S2 present characteristics of Datasets A and B. Age was significantly higher in those who died (*p* = 0.000). White blood cell (WBC) counts were significantly higher, while red blood cell and platelet counts, as well as hemoglobin and hematocrit levels, were lower in patients who died (*p* = 0.000 for all). Total bilirubin, direct bilirubin (D‐Bili), aspartate aminotransferase (AST), alanine aminotransferase, lactate dehydrogenase (LD), alkaline phosphatase, creatine kinase, amylase, C‐reactive protein (CRP), potassium (K), inorganic phosphorous, magnesium, blood urea nitrogen, creatinine, uric acid, random plasma glucose (RPG), ferritin, fibrin/fibrinogen degradation product (FDP), and D‐dimer levels; prothrombin time‐international normalized ratio (PT‐INR); and activated partial thromboplastin time (APTT) were significantly higher in non‐survivors than in survivors. By contrast, total protein (TP), albumin, chloride, calcium (Ca), total cholesterol (T‐CHO), triglyceride (TG), high‐density lipoprotein cholesterol (HDL‐C), low‐density lipoprotein cholesterol, fibrinogen, and antithrombin III levels were significantly lower in non‐survivors than in survivors.

### 3.3 Univariate and Multivariate Logistic Regression Analyses

Table S3 shows the univariate and multivariate logistic regression analyses. Higher WBC counts, total bilirubin, D‐Bili, AST, alanine aminotransferase, γ‐glutamyl transpeptidase, LD, alkaline phosphatase, creatine kinase, amylase, CRP, K, inorganic phosphorous, magnesium, blood urea nitrogen, creatinine, uric acid, RPG, iron, ferritin, FDP, and D‐dimer levels; PT‐INR; and APTT were associated with higher fatality. Lower red blood cell and platelet counts, as well as hemoglobin, Hct, TP, albumin, sodium, chloride, Ca, TC, TG, HDL‐C, LGL‐C, fibrinogen, and antithrombin III levels were also significantly associated with fatality.

### 3.4 Model 1 Created Using MLRA

Table 2 Analysis (1) presents the findings from multivariate logistic regression predicting 72‐h fatality in patients with D‐dimer levels ≥ 2 μg/mL in the training dataset. Five variables were included: age, TP, *log*‐transformed D‐dimer, AST, and TC. The logistic regression equation for estimating 72‐h fatality probability (*p*) is:

(1) $$\begin{array}{c} p=1/\left[1+\exp\;\left(1.370+0.025\;\left(\mathrm{Age}\right)–0.367\;\left(\mathrm{TP}\right)+0.433\;\log\;\left(D-\text{dimer}\right)\right.\right.\\ \left.\left.\;+0.450\;\log\;\left(\mathrm{AST}\right)–1.373\;\log\;\left(\mathrm{TC}\right)\right)\right] \end{array}$$

**TABLE 2:** Multivariate logistic regression analysis (Model 1) for predicting 72‐h fatality.

Analysis (1) Dataset‐A for training (D‐dimer ≥ 2 μg/mL: 2018–2019)								
Exp Var	MLRA: Obj Var = Death (N dead = 63, N alive = 1679)				VIF	*n* = 1742 (with all five Exp Vars)		
	*β*	SE (β)	*z*	*P*		OR		
	1.370	2.261						
Age (years)	0.025	0.011	2.36	0.0183	1.009	1.025	1.004	1.047
TP (g/dL)	−0.367	0.144	−2.55	0.0107	1.136	0.693	0.523	0.918
*log* (D‐dimer (μg/mL))	0.433	0.109	3.99	< 0.001	1.097	1.542	1.246	1.909
*log* (AST (U/L))	0.450	0.099	4.54	< 0.001	1.126	1.568	1.292	1.904
*log* (TC (mg/dL))	−1.373	0.456	−3.01	0.0026	1.064	0.253	0.104	0.619
AIC = 445.596, AUC = 0.829 (95% CI = 0.768–0.888), Sn = 0.762, Sp = 0.809								
*p* = 1/[1 + exp (1.370 + 0.025 (Age)−0.367 (TP) + 0.433 *log* (D‐dimer) + 0.450 *log* (AST)−1.373 *log* (TC))]								

Analysis (2) Dataset‐B for validation (D‐dimer ≥ 2 μg/mL: 2020–2021)							
ROC analysis to evaluate the accuracy of predicted probability (*p*) for fatal outcome							
N dead	N alive	AUC	95% CI of AUC	*n* = 1816 (with all 5 Exp Vars)			
75	1741	0.788	0.735–0.840				

Abbreviations: β, partial regression coefficient; AST, aspartate aminotransferase; AUC, area under the curve; CI, confidence interval; Exp Var, explanatory variable; MLRA, multivariate logistic regression analysis; Obj Var, object variable; OR, odds ratio; SE, standard error; Sn, sensitivity; Sp, specificity; TC, total cholesterol; TP, total protein.

**FIGURE 2:**
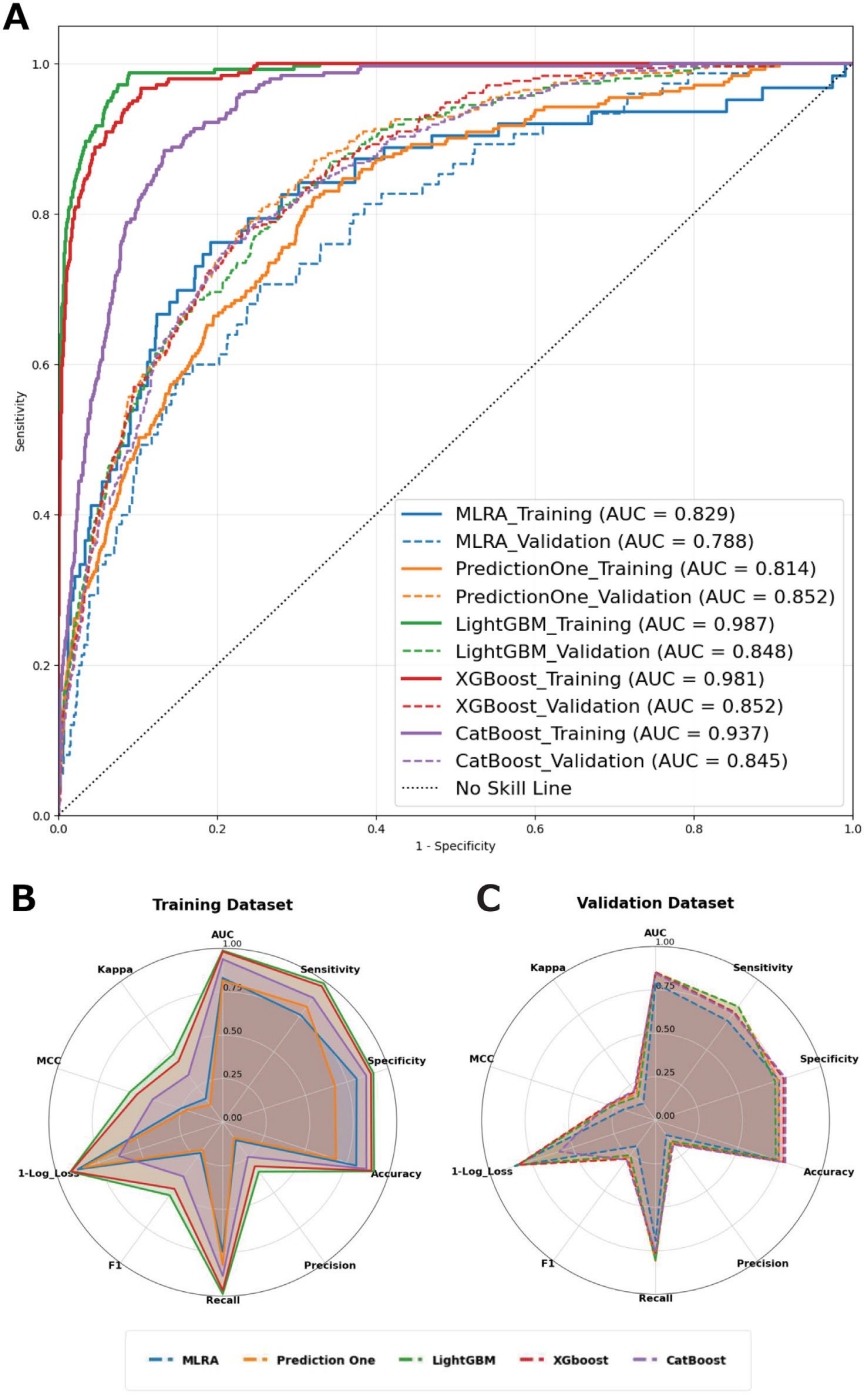
Comparison of ROC curves and radar plots among the five models between the training and validation datasets. The upper panel (A) illustrates the ROC curves for five predictive models—MLRA, Prediction One, LightGBM, XGBoost, and CatBoost—applied to both the training (2018–2019) and validation (2020–2021) datasets comprising patients with D‐dimer levels ≥ 2 μg/mL. In the training dataset, LightGBM achieved the highest AUC (0.987, 95% CI: 0.977–0.997), followed by XGBoost (AUC: 0.981, 95% CI: 0.966–0.992) and CatBoost (AUC: 0.937, 95% CI: 0.916–0.959). In the validation dataset, XGBoost yielded the highest AUC (0.852, 95% CI: 0.828–0.882), with Prediction One (AUC: 0.852, 95% CI: 0.825–0.879) and LightGBM (AUC: 0.848, 95% CI: 0.821–0.875) following closely. MLRA exhibited the lowest performance, with an AUC of 0.829 (95% CI: 0.768–0.888) and 0.788 (95% CI: 0.735–0.838) for the training and validation datasets, respectively. The lower panels (B, C) display radar plots summarizing multiple performance metrics (AUC, sensitivity, specificity, accuracy, precision, recall, F1 score, 1‐log loss, MCC, and Cohen's kappa) for each model in the training (B) and validation (C) datasets. The metric “1‐log loss” is used instead of “log loss” to harmonize the directionality of all metrics, so that higher values consistently indicate better performance. This enhances interpretability of the radar plots. LightGBM demonstrates superior discrimination and calibration in the training dataset, while XGBoost and Prediction One show the balanced performance across in the validation dataset. AUC, area under the curve; CatBoost, Categorical Boosting; CI, confidence interval; LightGBM, Light Gradient Boosting Machine; MCC, Matthews Correlation Coefficient; ROC, receiver operating characteristic; XGBoost, Extreme Gradient Boosting.

The results show that age, *log* D‐dimer, and *log* AST are positively correlated with fatality, while TP and *log* TC are negatively correlated, suggesting protective effects. The model achieved an AUC of 0.829 (95% CI: 0.768–0.888) (Figure 2A, blue solid line), with a sensitivity of 0.762 and specificity of 0.807, reflecting a balanced performance. Although precision was low at 0.128, the F1 score of 0.218 indicated a reasonable balance between precision and recall (Figure 2B, Table S4).

To evaluate whether the effect of D‐dimer on 72‐h fatality was influenced by malignancy, an exploratory interaction analysis was conducted using Firth's penalized logistic regression.

In the training cohort (*n* = 5158), 241 patients died within 72 h, including 41 (17.0%) due to malignancy. The model included an interaction term between *log* D‐dimer and malignancy status, yielding a non‐significant result (OR = 0.077, 95% CI: 0.000–2.42 × 10^5^, *p* = 0.398), indicating that the association between the D‐dimer level and short‐term fatality was not materially modified by malignancy status.

### 3.5 Model 2 Created by Prediction One

Table S4 compares the performance of Prediction One. The model achieved an AUC of 0.814, indicating high reliability (Figure 2A, yellow solid line). The sensitivity was 0.822 and specificity was 0.678, reflecting a tendency toward false positives. The F1 score of 0.196 suggested a balanced, though imperfect, performance (Figure 2B, Table S4). Elevated D‐Bil (2.3–23.8 mg/dL) and AST (100.7–15,100.0 U/L) levels, as well as decreased Ca (3.0–7.5 mg/dL) and Hct (8.3%–25.7%) levels, were strongly associated with fatality (Table S5).

### 3.6 Predictive Performance Comparison Among Models 3–5

The LightGBM, XGBoost, and CatBoost models were assessed (Figure 2A, Table S4). LightGBM was the top performer with an AUC of 0.987 (95% CI: 0.977–0.997), a high sensitivity (0.987) and specificity (0.911), and an F1 score of 0.519. XGBoost had an AUC of 0.981, with lower sensitivity (0.967), specificity (0.896), and F1 score (0.473) (Figure 2B, Table S4). CatBoost showed the lowest AUC of 0.937 and reduced consistency compared with the other models.

### 3.7 SHAP Analysis Among Models 3–5

**FIGURE 3:**
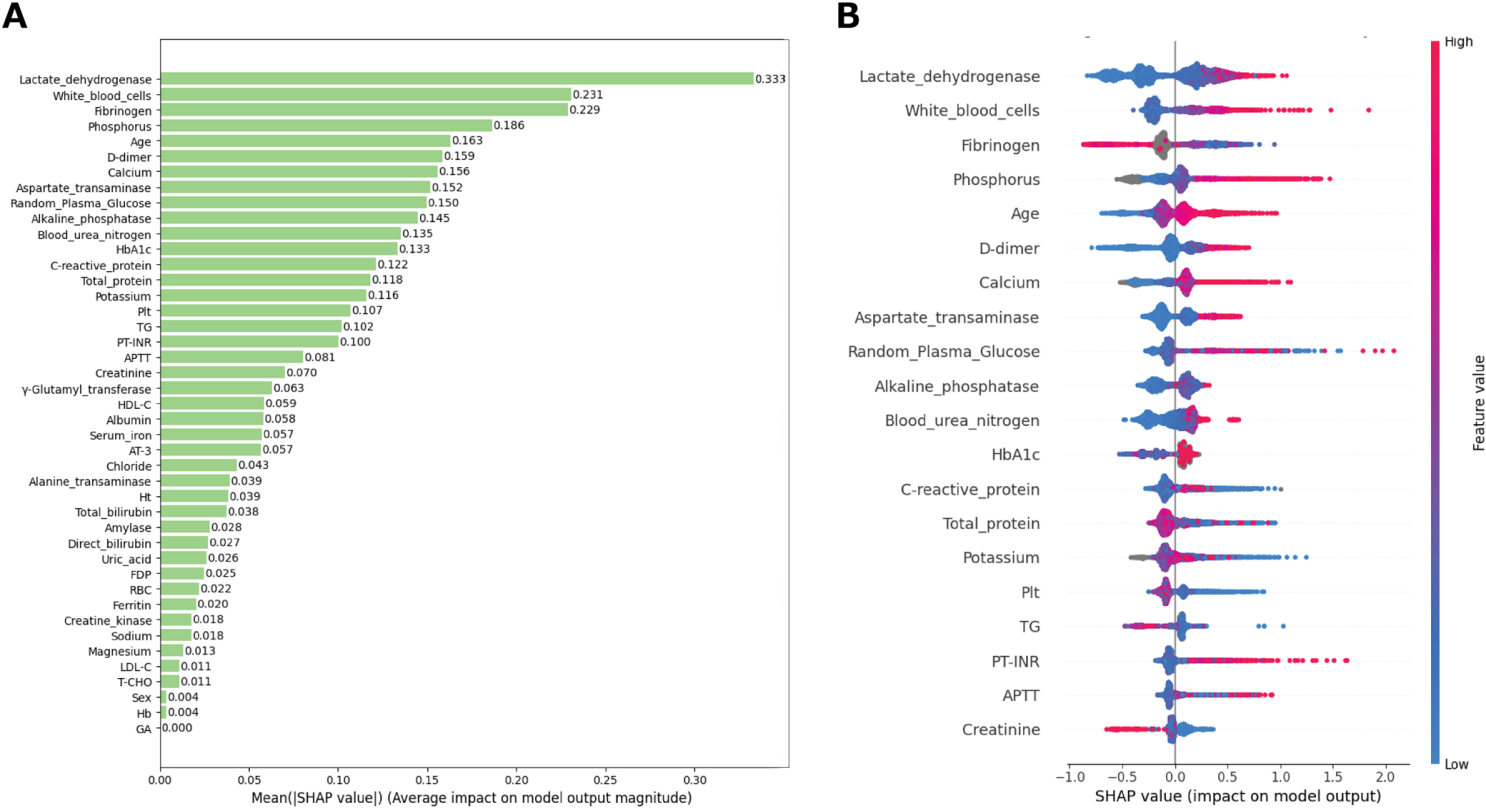
LightGBM SHAP summary and beeswarm plots. The left panel (A) displays the SHAP summary plot for the LightGBM model, showing the mean absolute SHAP values for all features, which indicate their average impact on the model's predictions. The plot highlights that lactate dehydrogenase (LD), fibrinogen, phosphorus levels, and white blood cell count are among the top predictors, with higher SHAP values indicating a greater influence on the predicted outcomes. The right panel (B) presents the SHAP beeswarm plot for the top 20 features ranked by their importance. The plot visualizes the distribution of SHAP values across all samples for these key features. The colors represent the feature values (blue for low and red for high). The beeswarm plot reveals that higher values of LD, white blood cell count, and fibrinogen tend to push the model's predictions toward a higher risk, particularly in patients with elevated D‐dimer levels. Nonlinear effects are evident in features such as potassium and total protein, where both low and high extremes significantly impact the model's predictions. LightGBM, Light Gradient Boosting Machine; SHAP, SHapley Additive exPlanations.

**FIGURE 4:**
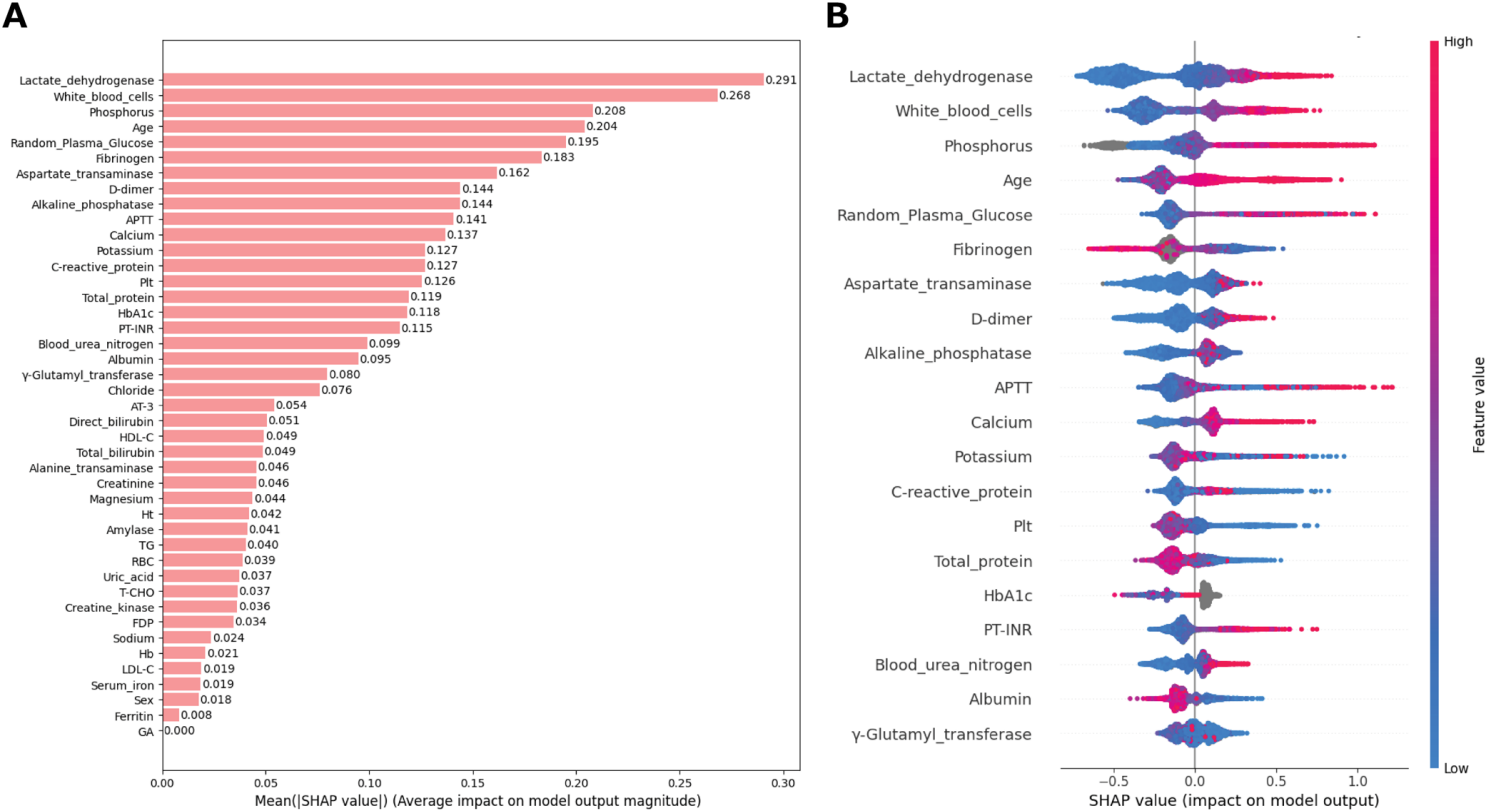
XGBoost SHAP summary and beeswarm plots. The left panel (A) presents the SHAP summary plot for the XGBoost model, illustrating the average impact of each feature on the model predictions. Lactate dehydrogenase (LD) level, white blood cell count, and phosphorus level are identified as the most influential features, with higher SHAP values indicating a greater contribution to the model's predictive outcomes. The right panel (B) shows the SHAP beeswarm plot for the top 20 features, showing the distribution of SHAP values across all samples. The colors represent the feature values, with blue indicating lower values and red indicating higher values. The plot reveals that higher levels of LD and phosphorous and increased white blood cell count are strongly associated with increased SHAP values, indicating a higher predicted risk. Additionally, nonlinear relationships are observed for features such as potassium and total protein, where both the low and high extremes result in significant shifts in SHAP values, highlighting their complex influence on the model predictions. SHAP, SHapley Additive exPlanations; XGBoost, Extreme Gradient Boosting.

**FIGURE 5:**
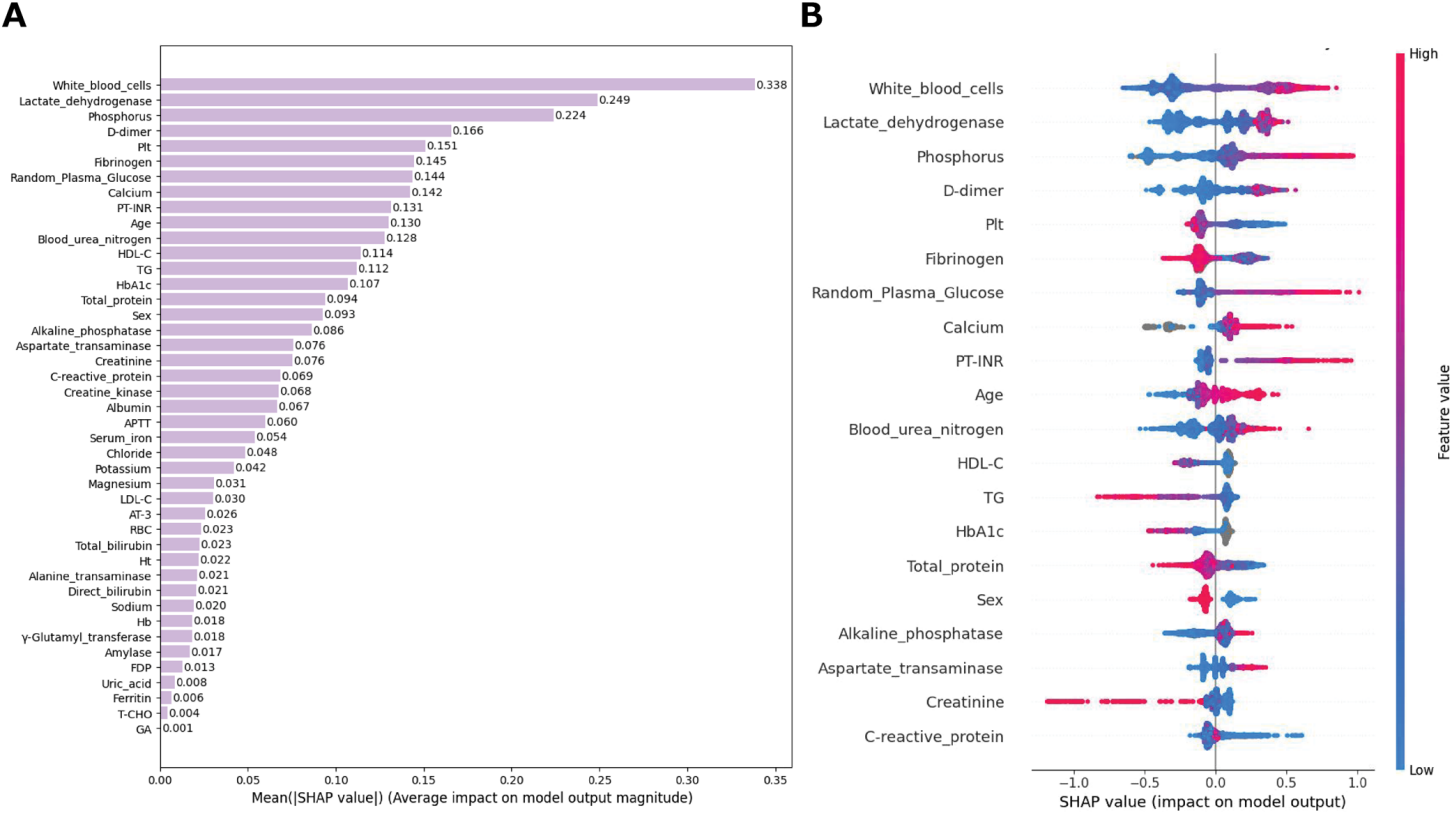
CatBoost SHAP summary and beeswarm plots. The left panel (A) presents the SHAP summary plot for the CatBoost model, which ranks features based on their average impact on the model's output. White blood cell count, lactate dehydrogenase (LD) and phosphorus levels are identified as the most influential features, with higher mean SHAP values indicating a stronger effect on the model predictions. The right panel (B) shows the SHAP beeswarm plot for the top 20 features, showing the SHAP values for individual samples. The plot uses color to represent the feature values, with blue indicating lower values and red indicating higher values. The beeswarm plot reveals that higher levels of LD and phosphorus and an increased white blood cell count are associated with increased SHAP values, signifying a higher predicted risk. Additionally, the plot indicates nonlinear effects for features such as calcium and total protein, where both the low and high values can significantly influence the predictions. CatBoost, Categorical Boosting; SHAP, SHapley Additive exPlanations.

SHAP analysis identified LD, WBC, fibrinogen, and phosphorus as the top predictors in the LightGBM model (Figure 3A,B), with similar key features highlighted in XGBoost (Figure 4A,B) and CatBoost models (Figure 5A,B). High LD level, WBC count (Figure S2), phosphorus level, and D‐dimer level, as well as advanced age (Figure S3), positively influence fatality predictions, while lower fibrinogen (Figure S2) levels also contribute to increased fatality. Non‐linear effects were evident across features such as fibrinogen (Figure S2), RPG (Figure S4), alkaline phosphatase, BUN, HbA1c (Figure S5), CRP, total TP, potassium (Figure S6), platelet counts, TG (Figure S7), APTT, creatinine, and γ‐glutamyl transpeptidase (Figure S8) demonstrating complex influences on predicted risk.

### 3.8 Bootstrap Internal Validations for Predictive Models: MLRA, LightGBM, XGBoost, and CatBoost

Bootstrap validation results (Table S6) showed that LightGBM and XGBoost outperformed MLRA and CatBoost in terms of AUC and accuracy, with minimal optimism bias. LightGBM had an AUC of 0.933, while XGBoost achieved 0.935. MLRA had the lowest AUC at 0.830.

### 3.9 External Validation Results for Predictive Models

The external validation of the models demonstrated varying performance across metrics (Figure 2A,C, Table 2 analysis (2), Table S4). MLRA showed moderate discriminatory ability with an AUC of 0.788, sensitivity of 0.707, specificity of 0.747, and accuracy of 0.744. However, precision was low at 0.105, leading to a poor F1 score of 0.183, indicating a high rate of false positives.

Prediction One achieved better results, with an AUC of 0.852, sensitivity of 0.809, specificity of 0.743, and accuracy of 0.747. The precision improved to 0.157, with an F1 score of 0.263, indicating a more balanced performance compared to MLRA.

LightGBM performed similarly to Prediction One, with an AUC of 0.848, sensitivity of 0.809, specificity of 0.723, and accuracy of 0.727. Its precision was 0.147, and the F1 score was 0.248, showing good predictive capability but a slightly lower balance between precision and recall.

XGBoost matched Prediction One with an AUC of 0.852, sensitivity of 0.770, specificity of 0.774, and accuracy of 0.774. The precision was higher at 0.167, resulting in an F1 score of 0.275, demonstrating fewer false positives compared to MLRA.

CatBoost recorded an AUC of 0.845, sensitivity of 0.761, specificity of 0.786, and accuracy of 0.784. It showed the highest precision among all models at 0.173, with the best F1 score of 0.282, indicating a strong balance between precision and recall. However, the model's higher Log Loss of 0.418 suggested potential calibration issues, possibly reflecting overfitting.

In summary, Prediction One, LightGBM, and XGBoost demonstrated strong and comparable performance, with CatBoost excelling in balancing precision and recall, though showing potential overfitting concerns. MLRA performed poorly, particularly in handling false positives, emphasizing the benefits of ML models for achieving more balanced predictions in clinical applications.

### 3.10 Calibration Plots Comparison

**FIGURE 6:**
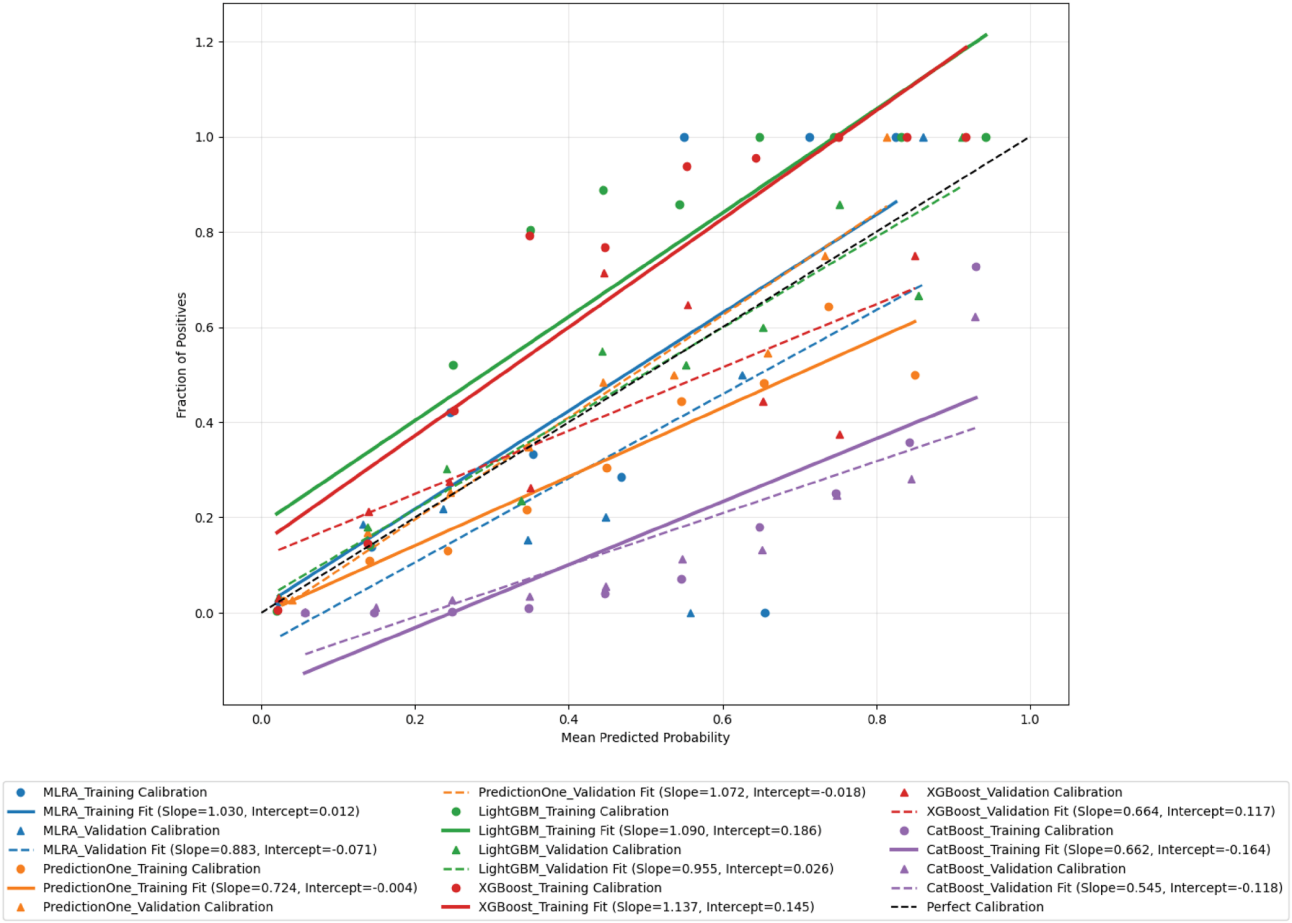
Calibration plots for the five models between the training and validation datasets. Calibration plots for the five predictive models—MLRA, Prediction One, LightGBM, XGBoost, and CatBoost—evaluated on both the training (2018–2019) and validation (2020–2021) datasets of patients with D‐dimer levels ≥ 2 μg/mL. Calibration curves are plotted with the mean predicted probability on the X‐axis and the fraction of positives (observed outcomes) on the Y‐axis. The closer the calibration curve is to the diagonal line (perfect calibration), the better the alignment of the model's predicted probabilities with the actual outcomes. The regression line statistics for the calibration fit of each model are summarized in Table S7. For example, in the training dataset, MLRA shows a slope of 1.030 and an intercept of 0.004; whereas in the validation dataset, it shows a slope of 0.879 and an intercept of −0.071. LightGBM, which shows a slope close to 1 (training: 1.090, validation: 0.955) and low intercept values (training: 0.186, validation: 0.026), demonstrates strong calibration in both datasets. Conversely, models such as XGBoost and CatBoost show deviations from the ideal slope of 1 in the validation dataset (XGBoost slope: 0.664, CatBoost slope: 0.545), indicating the need for further calibration refinement. CatBoost, Categorical Boosting; LightGBM, Light Gradient Boosting Machine; MLRA, multivariate logistic regression analysis; XGBoost, Extreme Gradient Boosting.

The calibration plots, shown in Figure 6 and Table S7, illustrate the alignment between predicted probabilities and observed outcomes across models. MLRA displayed adequate calibration with a training slope of 1.030 and a validation slope of 0.883, suggesting minor overestimation in the validation dataset. Intercepts near zero indicated overall good calibration, though slight adjustments may improve external validation.

Prediction One offered the best calibration, with a training slope of 0.724 and a validation slope of 1.072, reflecting minimal underestimation. High R‐squared values and near‐zero intercepts confirmed the model's consistent reliability across datasets.

LightGBM showed solid calibration, with a training slope of 1.090 and a validation slope of 0.955, indicating minor overestimation in validation. Intercepts were close to zero, supporting strong calibration; though a slight overfit might be present in the training phase.

XGBoost, however, revealed a significant gap between training and validation, with a training slope of 1.137 but a validation slope dropping to 0.664, indicating overestimation in external data, despite near‐zero intercepts. This calls for calibration adjustments.

CatBoost showed the most overestimation, with training and validation slopes of 0.662 and 0.545, respectively, struggling to generalize from training to validation data, as evidenced by lower slopes and negative intercepts.

In summary, Prediction One and LightGBM provided the most reliable calibration, while MLRA performed reasonably with slight overestimation. XGBoost and CatBoost required further calibration for improved external reliability.

## 4 Discussion

This study presents four primary findings: (1) 72‐h fatality rates increased with higher D‐dimer levels, indicating a concentration‐dependent trend; (2) intracranial diseases were the leading cause of death, followed by malignancies, sepsis, coronary artery disease, aortic disease, and pneumonia; (3) the MLRA model identified five key predictors of fatality—elevated D‐dimer and AST levels, advanced age, and low TP and TC levels; and (4) LightGBM provided the best discrimination, calibration, and clinical utility among all the models evaluated. These findings underscore the potential of data‐driven tools to improve early risk stratification in critical care.

Focusing on 72‐h fatality is clinically pertinent, because this period marks a well‐established window for deterioration in emergency, intensive, and disaster medicine [15, 16, 17, 18, 19, 20, 21, 22, 23]. Our model, tailored to this time frame, may thus support prompt triage and appropriate allocation of resources.

A key strength of our model is its reliance on objective laboratory values alone. While this excludes contextual factors, such as testing conditions, anticoagulant use, and clinical setting, it mirrors the real‐world trade‐off clinicians' face when making urgent decisions without detailed patient information. This enhances generalizability across diverse care environments. However, future prospective validation incorporating structured clinical metadata is essential to strengthen interpretability and robustness.

The MLRA model demonstrated high predictive performance for 72‐h fatality in patients with D‐dimer levels ≥ 2 μg/mL. The identified predictors—D‐dimer [33], AST [19, 34], age [20, 35, 36], TP [36, 37], and TC [38, 39]—are all established markers of poor prognosis in critical illness. Elevated AST levels [19, 34] are associated with cellular injury and mortality in hepatic, cardiac, and malignant conditions. A recent study also identified AST as a significant predictor of 72‐h fatality in patients with severe hyperphosphatemia (≥ 10 mg/dL) [35]. Age and hypoalbuminemia are consistently associated with adverse outcomes [20, 36], while hypocholesterolemia may reflect underlying inflammation and vulnerability to acute stress [38, 39]. These findings highlight the interplay between inflammation, metabolic health, and acute phase responses, suggesting that maintaining adequate TP and TC levels may protect against acute stressors.

The increase in fatality rates with rising D‐dimer levels reinforces its role as a marker of coagulation and fibrinolysis activation. Prior studies have associated high D‐dimer levels with mortality in sepsis, thromboembolism, cardiovascular diseases, malignancy, and COVID‐19 [7, 8, 9, 10, 11, 12, 13, 33]. Incorporating D‐dimer stratification into clinical workflows could optimize resource allocation and treatment strategies. For example, integrating D‐dimer into the Wells score improves predictive accuracy for venous thromboembolism [40].

Given that coagulation abnormalities are frequently associated with malignancy, we hypothesized that it might intensify the prognostic impact of elevated D‐dimer levels on short‐term fatality. However, the interaction between *log*‐transformed D‐dimer and malignancy was not significant (*p* = 0.398), indicating that the predictive value of D‐dimer remains consistent in both malignant and non‐malignant populations.

LightGBM outperformed other models, showing excellent calibration and discrimination, making it well‐suited for clinical use. Gradient boosting models (GBMs) such as LightGBM have proven effective in predicting outcomes in various conditions, including intradialytic hypotension, sepsis, and acute kidney injury [41, 42, 43]. With an AUC of 0.987 in training and 0.848 in validation, it showed strong predictive capability. XGBoost, while showing similar discrimination, had less optimal calibration, overestimating probabilities in validation data. Prediction One demonstrated consistent calibration, though with a slightly lower AUC in validation. CatBoost, while balanced, had a lower AUC, indicating less discrimination. MLRA, although interpretable, showed the lowest performance, with an AUC of 0.788 and weaker calibration, making it less suitable for complex clinical settings.

Comparative studies show ML models outperforming traditional regression models in predictive accuracy [26, 44]. LightGBM had the best performance in various clinical settings [45, 46], while CatBoost was reported to exhibit the highest AUC using clinical variables to predict early mortality in patients with sepsis [47]. Logistic regression remains effective for specific tasks such as predicting hospitalization time [48]. Prediction One has shown accuracy in the preoperative diagnosis of metastatic rectal cancer [49].

GBMs require meticulous hyperparameter tuning/optimization. Grid search systematically explores combinations but is computationally intensive. Random search reduces cost by sampling random combinations, often yielding comparable results. Bayesian optimization, used in tools such as Optuna, improves efficiency by leveraging previous results to focus on promising areas; though it still requires iterative adjustments [50]. Careful tuning is essential to prevent overfitting or underfitting [51]. In this study, hyperparameter settings were determined manually through trial and error, selecting values that best balanced model performance and overfitting prevention.

Future research should compare various ML models using diverse datasets to identify the best approach for specific clinical needs. Integrating high‐performing models, such as LightGBM, into electronic health records could enhance real‐time decision‐making and improve patient outcomes.

## 5 Limitations

This study has several limitations. First, its single‐center retrospective design limits generalizability. Second, testing context and anticoagulant use were unrecorded, introducing potential confounding. Third, incomplete comorbidity data limited adjustment for other conditions. Fourth, MLRA is more susceptible to bias from missing values, potentially weakening its robustness relative to ML models. Finally, while the 72‐h endpoint is clinically meaningful, future studies should evaluate longer‐term outcomes.

## 6 Conclusion

This study found approximately 5% fatality rate in patients with D‐dimer ≥ 2 μg/mL and identified five predictors of 72‐h fatality: elevated D‐dimer and AST levels, advanced age, and low TP and TC levels. LightGBM outperformed MLRA and other GBMs in both accuracy and calibration. Integrating these models into electronic health records can provide real‐time decision support, improving patient outcomes in critical care.

## Disclosure

Clinical Trial Registration: This study is a retrospective cohort study and was not prospectively registered as a clinical trial.

## Ethics Statement

This study was conducted in accordance with the principles of the Declaration of Helsinki. The study protocol was approved by the Institutional Clinical Research Ethics Review Board of Jichi Medical University, Saitama Medical Center, Saitama, Japan (Clinical Approval #S21‐100 on January 11, 2022, and #S24‐178 on April 28, 2025).

## Consent

The requirement for informed consent was waived by the Institutional Clinical Research Ethics Review Board of Jichi Medical University, Saitama Medical Center, Saitama, Japan in view of the retrospective study design and usage of anonymized data.

## Conflicts of Interest

The authors declare no conflicts of interest.

## Supporting information


**DATA S1:** jcla70091‐sup‐0001‐supinfo01.docx.

**DATA S2:** jcla70091‐sup‐0002‐supinfo02.docx.
Supplementary Text 1: Sample Size Estimation.
Supplementary Text 2: Routine Laboratory Tests.
Supplementary Text 3: List of Variables.
Supplementary Text 4: Box‐Cox Transformation Formulae.
Supplementary Text 5: Model 1 Development Using Multivariate Logistic Regression Analysis (MLRA).
Supplementary Text 6: Model 2 Development Using Prediction One.
Supplementary Text 7: Hyperparameters for Gradient Boosting Decision Trees (GBDTs).
Supplementary Text 8: Bootstrap Internal Validation.
Supplementary Text 9: External Validation of Models 1 to 5.
Supplementary Text 10: Statistical Software and Programming Details.

**DATA S3:** jcla70091‐sup‐0003‐Figures.docx.
**FIGURE S1:** Patient selection flow diagram.
**FIGURES S2–S8:** SHAP dependence plots.
**FIGURE S3:** SHAP dependence plots for phosphorus, age, and D‐dimer Phosphorus.
**FIGURE S4:** SHAP dependence plots for calcium, AST, and RPG calcium.
**FIGURE S5:** SHAP dependence plots for ALP, BUN, and HbA1c alkaline phosphatase (ALP).
**FIGURE S6:** SHAP dependence plots for CRP, total protein, and potassium.
**FIGURE S7:** SHAP dependence plots for platelet counts, TG, and PT‐INR platelet counts (Plt).
**FIGURE S8:** SHAP dependence plots for APTT, creatinine, and γ‐GTP activated partial thromboplastin time (APTT).

**DATA S4:** jcla70091‐sup‐0004‐Tables.docx.
**TABLE S1:** Patient demographics, and laboratory test values of training dataset.
**TABLE S2:** Patient demographics, and laboratory test values of validation dataset.
**TABLE S3:** Univariate and multivariate logistic regression analysis results.
**TABLE S4:** Comparison of AUC, 95% CI, sensitivity, specificity, accuracy, precision, recall, F1, log loss, MCC, and Cohen's Kappa.
**TABLE S5:** Degree of contribution of variables and the most contributive ranges to 72‐h outcomes of prediction one.
**TABLE S6:** Bootstrap statistics among MLRA, LightGBM, XGBoost, CatBoost.
**TABLE S7:** Comparison of statistics for the regression lines of the calibration plot.

## Data Availability

The datasets generated and analyzed during the current study are not publicly available due to institutional regulations but are available from the corresponding author upon reasonable request and with permission from the Institutional Clinical Research Ethics Review Board of Jichi Medical University, Saitama Medical Center, Saitama, Japan. Data requests must comply with ethical guidelines and institutional data‐sharing policies.
