# Supplementary material for: Routine Laboratory Tests Predict 72‐h Fatality in Patients With D‐Dimer Levels ≥ 2 μg/mL: A Retrospective Cohort Study Comparing Statistical and Machine Learning Models
Source: J Clin Lab Anal. 2025 Sep 3;39(18):e70091. doi: 10.1002/jcla.70091 (PMC12459218; doi:10.1002/jcla.70091)
Supplement: Supplementary file 1 — DATA S1: jcla70091‐sup‐0001‐supinfo01.docx. [file JCLA-39-e70091-s001.docx]

**Supplementary program codes**

**Python program codes for statistical and machine learning analysis in the study**

"""

**Title: D-dimer Stratification and Fatality Rate Analysis in the Training and Validation Datasets**

Description:

This Python script performs a detailed stratification of D-dimer levels and calculates the fatality rates within each stratum for both the training and validation datasets. The script also includes a chi-square test to compare the fatality rates between the two datasets. The final output is a bar plot visualizing the stratified fatality rates with significance marks for *p*-values, accompanied by standard errors.

"""

**README**

**Overview**

This Python program is designed to analyze and visualize the fatality rates of patients stratified by D-dimer levels, using both the training and validation datasets. It calculates the fatality rate within predefined D-dimer concentration strata and performs statistical analysis, including a chi-square test, to compare the training and validation datasets. The program generates a bar plot of the stratified fatality rates, including an overall comparison between the datasets.

**Requirements**

- Python 3.x
- Required libraries: pandas, numpy, matplotlib, scipy

Install the necessary libraries using:

pip install pandas numpy matplotlib scipy

**How to Use**

1. **Prepare Data:**
   - Place your training and validation datasets in CSV format.
   - Ensure that the D-dimer values are in the 43rd column (column AQ), and the outcome variable (death/alive) is in the 4th column (column D).
2. **Modify File Paths:**
   - Update the file paths in the script to point to your datasets:

df_train = pd.read_csv("path/to/training_data.csv")

df_test = pd.read_csv("path/to/validation_data.csv")

1. **Run the Script:**
   - Execute the script using Python:

python script_name.py

- - The program will output the following:
    - Stratified counts and fatality rates for the training and validation data.
    - Results of the chi-square tests for each stratum and overall.
    - A bar plot (d_dimer_fatality_rate_overall_bar_plot.png) showing the stratified fatality rates with significance marks where applicable.

1. **Interpretation:**
   - The generated bar plot visualizes the fatality rates stratified by D-dimer levels for both the training and validation datasets, with error bars representing standard error. Significance marks (*) indicate strata where the fatality rates significantly differ between datasets (*p* < 0.05).

**Output**

- **Bar Plot:** d_dimer_fatality_rate_overall_bar_plot.png is saved in the current directory, showing the stratified fatality rates.
- **Console Output:** Displays the calculated counts, fatality rates, and chi-square test results.

**Notes**

- Ensure that the D-dimer values and outcome columns are correctly referenced, as they are critical for accurate stratification and analysis.
- The chi-square test results help assess whether the differences in fatality rates between the training and validation datasets are statistically significant.

**Example**

For demonstration purposes, the script uses generalized file paths. Replace these with the actual paths to your datasets.

This README provides a brief overview and instructions to help you use the program effectively. For more detailed analysis or customization, you may need to modify specific parts of the code.

import pandas as pd

import numpy as np

import matplotlib.pyplot as plt

from scipy.stats import chi2_contingency

# Load data

df_train = pd.read_csv("path/to/training_data.csv") # Generalized file path

df_test = pd.read_csv("path/to/validation_data.csv") # Generalized file path

# Extract relevant columns

d_dimer_train = df_train.iloc[:, 42].astype(float) # AQ column

outcome_train = df_train.iloc[:, 3].astype(int) # D column

d_dimer_test = df_test.iloc[:, 42].astype(float) # AQ column

outcome_test = df_test.iloc[:, 3].astype(int) # D column

# Define new bins for stratification

max_concentration = max(d_dimer_train.max(), d_dimer_test.max())

bins = [2.0, 2.1, 3.0, 4.0, 5.0, 10.0, 100.0, max_concentration + 1]

labels = ["2.0", "2.1 - 3.0", "3.1 - 4.0", "4.1 - 5.0", "5.1 - 10.0", "10.1 - 100.0", "100.1 -"]

# Stratify data

train_stratified = pd.cut(d_dimer_train, bins=bins, labels=labels, right=False)

test_stratified = pd.cut(d_dimer_test, bins=bins, labels=labels, right=False)

# Function to calculate stratified counts and fatality rates with standard error

def calculate_counts_and_fatality_rate(outcome, stratified):

stratified_counts = pd.DataFrame(index=labels, columns=['Death', 'Alive', 'Total'])

stratified_counts['Death'] = stratified[outcome == 1].value_counts().reindex(labels, fill_value=0)

stratified_counts['Alive'] = stratified[outcome == 0].value_counts().reindex(labels, fill_value=0)

stratified_counts['Total'] = stratified_counts['Death'] + stratified_counts['Alive']

stratified_counts['Fatality Rate'] = stratified_counts['Death'] / stratified_counts['Total']

stratified_counts['Standard Error'] = np.sqrt(

(stratified_counts['Fatality Rate'] * (1 - stratified_counts['Fatality Rate'])) / stratified_counts['Total']

)

return stratified_counts

# Calculate counts and fatality rates for training and test data

train_fatality_rate = calculate_counts_and_fatality_rate(outcome_train, train_stratified)

test_fatality_rate = calculate_counts_and_fatality_rate(outcome_test, test_stratified)

# Calculate overall statistics for training and validation data

def calculate_overall_stats(fatality_rate):

overall_stats = pd.DataFrame({

'Death': [fatality_rate['Death'].sum()],

'Alive': [fatality_rate['Alive'].sum()],

'Total': [fatality_rate['Total'].sum()]

})

overall_stats['Fatality Rate'] = overall_stats['Death'] / overall_stats['Total']

overall_stats['Standard Error'] = np.sqrt(

(overall_stats['Fatality Rate'] * (1 - overall_stats['Fatality Rate'])) / overall_stats['Total']

)

return overall_stats

overall_train_stats = calculate_overall_stats(train_fatality_rate)

overall_test_stats = calculate_overall_stats(test_fatality_rate)

# Append overall statistics to the stratified data

train_fatality_rate = pd.concat([train_fatality_rate, overall_train_stats], axis=0, ignore_index=False)

train_fatality_rate.index = labels + ['Overall']

test_fatality_rate = pd.concat([test_fatality_rate, overall_test_stats], axis=0, ignore_index=False)

test_fatality_rate.index = labels + ['Overall']

# Chi-square test for each stratum including Overall

chi2_results = pd.DataFrame(index=labels + ['Overall'], columns=['Chi2', 'p-value'])

for label in labels + ['Overall']:

train_death = train_fatality_rate.loc[label, 'Death']

train_alive = train_fatality_rate.loc[label, 'Alive']

test_death = test_fatality_rate.loc[label, 'Death']

test_alive = test_fatality_rate.loc[label, 'Alive']

contingency_table = np.array([[train_death, train_alive], [test_death, test_alive]])

chi2, p, _, _ = chi2_contingency(contingency_table)

chi2_results.loc[label] = [chi2, p]

# Output the statistical indicators

print("Training Data Stratified Counts and Fatality Rates (including Overall):")

print(train_fatality_rate)

print("\nValidation Data Stratified Counts and Fatality Rates (including Overall):")

print(test_fatality_rate)

print("\nChi-square Test Results:")

print(chi2_results)

# Update the x positions and width to include the overall bar

x = np.arange(len(labels) + 1)

width = 0.35 # the width of the bars

# Adjust the plotting to include the overall fatality rates

fig, ax = plt.subplots(figsize=(14, 8))

rects1 = ax.bar(x - width/2, train_fatality_rate['Fatality Rate'], width,

yerr=train_fatality_rate['Standard Error'],

label='Training Data', color='lightgrey', capsize=5)

rects2 = ax.bar(x + width/2, test_fatality_rate['Fatality Rate'], width,

yerr=test_fatality_rate['Standard Error'],

label='Validation Data', color='darkgrey', capsize=5)

# Update xticks to include 'Overall'

ax.set_xticks(x)

ax.set_xticklabels(labels + ['Overall'])

# Add significance marks for p-values

for i, p_val in enumerate(chi2_results['p-value']):

if p_val < 0.05:

# Drawing the significance bracket

y_max = max(train_fatality_rate.iloc[i, 3], test_fatality_rate.iloc[i, 3])

ax.plot([x[i] - width/2, x[i] - width/2, x[i] + width/2, x[i] + width/2],

[y_max + 0.02, y_max + 0.04, y_max + 0.04, y_max + 0.02],

lw=1.5, color='black')

ax.text(x[i], y_max + 0.045, '*', ha='center', va='bottom', color='black', fontsize=16)

# Add labels to the overall bars

ax.annotate(f'{overall_train_stats["Fatality Rate"].values[0]:-.2%}',

xy=(x[-1] - width/2, overall_train_stats["Fatality Rate"].values[0] + overall_train_stats["Standard Error"].values[0] + 0.01),

xytext=(0, -15), # 15 points vertical offset downwards

textcoords="offset points",

ha='center', va='bottom')

ax.annotate(f'{overall_test_stats["Fatality Rate"].values[0]:-.2%}',

xy=(x[-1] + width/2, overall_test_stats["Fatality Rate"].values[0] + overall_test_stats["Standard Error"].values[0] + 0.01),

xytext=(0, -15), # 15 points vertical offset downwards

textcoords="offset points",

ha='center', va='bottom')

# Add some text for labels, title and custom x-axis tick labels, etc.

ax.set_xlabel('D-dimer (µg/mL)')

ax.set_ylabel('Fatality Rate')

ax.set_title('D-dimer Stratified Fatality Rates')

ax.legend()

# Customize the grid

ax.grid(True, which='both', linestyle='--', linewidth=0.5)

# Function to add labels on bars

def autolabel(rects, errors):

"""Attach a text label above each bar in *rects*, displaying its height."""

for rect, error in zip(rects, errors):

height = rect.get_height()

ax.annotate(f'{height:.2%}',

xy=(rect.get_x() + rect.get_width() / 2, height + error + 0.01),

xytext=(0, -15), # 15 points vertical offset downwards

textcoords="offset points",

ha='center', va='bottom')

autolabel(rects1, train_fatality_rate['Standard Error'])

autolabel(rects2, test_fatality_rate['Standard Error'])

fig.tight_layout()

# Save and show the updated plot

plt.savefig('d_dimer_fatality_rate_overall_bar_plot.png')

plt.show()

"""

**Title: Logistic Regression Model with Bootstrap Validation and Performance Metrics for D-dimer Data**

Description:

This Python script implements a predictive model using logistic regression to analyze the relationship between D-dimer levels and patient outcomes. It includes data preprocessing, model fitting, and evaluation using various statistical metrics such as AUC, sensitivity, specificity, and more. The script also performs a bootstrap validation with 1,000 resamples to assess the model's stability and outputs ROC curves, calibration plots, and detailed performance metrics with confidence intervals.

"""

**README**

**Overview**

This Python program performs logistic regression analysis on medical datasets to predict patient outcomes based on various biochemical parameters. It calculates and evaluates multiple performance metrics, including AUC, accuracy, precision, and F1 score. Additionally, it conducts bootstrap internal validation to assess model stability and produces ROC curves, calibration plots, and a bootstrap AUC distribution plot.

**Requirements**

- Python 3.x
- Required libraries: pandas, numpy, scikit-learn, matplotlib, statsmodels, scipy

Install the necessary libraries using:

pip install pandas numpy scikit-learn matplotlib statsmodels scipy

**How to Use**

1. **Prepare Data:**
   - Place your training and validation datasets in CSV format.
   - Ensure that the dataset includes the following columns:
     - Age: Age of the patient
     - TP: Total protein
     - D-dimer: D-dimer levels
     - AST: Aspartate aminotransferase
     - TC: Total cholesterol
     - Outcome: Binary outcome variable (1 for death, 0 for survival)
2. **Modify File Paths:**
   - Update the file paths in the script to point to your datasets:

df_train = pd.read_csv("path/to/training_data.csv")

df_test = pd.read_csv("path/to/validation_data.csv")

1. **Run the Script:**
   - Execute the script using Python:

python script_name.py

- - The program will:
    - Log-transform relevant biochemical variables (D-dimer, AST, and TC).
    - Fit a logistic regression model and calculate predicted probabilities.
    - Evaluate the model on both the training and validation datasets using multiple metrics.
    - Perform bootstrap validation to calculate confidence intervals for AUC and other metrics.
    - Generate and display plots, including ROC curves, calibration plots, and AUC distribution from bootstrap samples.

1. **Interpretation:**
   - **ROC Curve:** Visualize model performance across various threshold settings. Compare the training and validation datasets.
   - **Calibration Plot:** Assess how well the predicted probabilities correspond to actual outcomes.
   - **Bootstrap AUC Distribution:** Evaluate the variability in model performance.
   - **Performance Metrics:** Metrics such as accuracy, precision, F1 score, and AUC are displayed for both datasets, along with their 95% confidence intervals derived from bootstrap samples.
2. **Output:**
   - **ROC Curve Plot:** Displays the ROC curves for both the training and validation datasets.
   - **Bootstrap AUC Distribution Plot:** Shows the distribution of AUC scores from bootstrap samples.
   - **Calibration Plot:** Compares predicted probabilities against actual observed probabilities.
   - **Bootstrap ROC Curve Example Plot:** Displays the ROC curve from an example bootstrap sample.
   - **Performance Metrics:** Printed in the console, including sensitivity, specificity, accuracy, precision, F1 score, AUC, log loss, MCC, and Cohen’s Kappa, with 95% CIs.
3. **Customization:**
   - The script can be modified to include additional variables, change the logistic regression coefficients, or adjust the number of bootstrap samples.

**Example**

For demonstration purposes, the script uses generalized file paths. Replace these with the actual paths to your datasets to perform the analysis.

# Import libraries

import pandas as pd

import numpy as np

from sklearn.metrics import (roc_curve, auc, accuracy_score, f1_score,

precision_score, recall_score, log_loss,

matthews_corrcoef, cohen_kappa_score, roc_auc_score)

from sklearn.utils import resample

from sklearn.calibration import calibration_curve

import matplotlib.pyplot as plt

from statsmodels.tools.tools import add_constant

from scipy import stats

# Load data

df_train = pd.read_csv("path/to/training_data.csv") # Generalized file path

df_test = pd.read_csv("path/to/validation_data.csv") # Generalized file path

# Remove rows with missing values

df_train = df_train.dropna()

df_test = df_test.dropna()

# Set explanatory and target variables

X_train = df_train[['Age', 'TP', 'D-dimer', 'AST', 'TC']]

X_test = df_test[['Age', 'TP', 'D-dimer', 'AST', 'TC']]

y_train = df_train['Outcome']

y_test = df_test['Outcome']

# Log-transform the relevant variables

X_train['Log_D-dimer'] = np.log(X_train['D-dimer'])

X_train['Log_AST'] = np.log(X_train['AST'])

X_train['Log_TC'] = np.log(X_train['TC'])

X_test['Log_D-dimer'] = np.log(X_test['D-dimer'])

X_test['Log_AST'] = np.log(X_test['AST'])

X_test['Log_TC'] = np.log(X_test['TC'])

# Use the transformed variables in the prediction

X_train = X_train[['Age', 'TP', 'Log_D-dimer', 'Log_AST', 'Log_TC']]

X_test = X_test[['Age', 'TP', 'Log_D-dimer', 'Log_AST', 'Log_TC']]

# Add intercept to explanatory variables

X_train = add_constant(X_train)

X_test = add_constant(X_test)

# Calculate prediction probabilities

def predict_proba(X):

beta = np.array([1.370, 0.025, -0.367, 0.433, 0.450, -1.373])

z = np.dot(X, beta)

p = 1 / (1 + np.exp(-z))

return p

# Calculate prediction probabilities

y_train_pred_proba = predict_proba(X_train)

y_test_pred_proba = predict_proba(X_test)

# ROC curve and AUC for training data

fpr_train, tpr_train, thresholds_train = roc_curve(y_train, y_train_pred_proba)

auc_score_train = auc(fpr_train, tpr_train)

optimal_idx_train = np.argmax(tpr_train - fpr_train)

optimal_threshold_train = thresholds_train[optimal_idx_train]

sensitivity_train = tpr_train[optimal_idx_train]

specificity_train = 1 - fpr_train[optimal_idx_train]

# ROC curve and AUC for test data

fpr_test, tpr_test, thresholds_test = roc_curve(y_test, y_test_pred_proba)

auc_score_test = auc(fpr_test, tpr_test)

optimal_idx_test = np.argmax(tpr_test - fpr_test)

optimal_threshold_test = thresholds_test[optimal_idx_test]

sensitivity_test = tpr_test[optimal_idx_test]

specificity_test = 1 - fpr_test[optimal_idx_test]

# Calculate evaluation metrics

y_train_pred = (y_train_pred_proba > optimal_threshold_train).astype(int)

y_test_pred = (y_test_pred_proba > optimal_threshold_test).astype(int)

accuracy_train = accuracy_score(y_train, y_train_pred)

f1_train = f1_score(y_train, y_train_pred)

precision_train = precision_score(y_train, y_train_pred)

recall_train = recall_score(y_train, y_train_pred)

accuracy_test = accuracy_score(y_test, y_test_pred)

f1_test = f1_score(y_test, y_test_pred)

precision_test = precision_score(y_test, y_test_pred)

recall_test = recall_score(y_test, y_test_pred)

# Calculate Log Loss

log_loss_train = log_loss(y_train, y_train_pred_proba)

log_loss_test = log_loss(y_test, y_test_pred_proba)

# Calculate MCC

mcc_train = matthews_corrcoef(y_train, y_train_pred)

mcc_test = matthews_corrcoef(y_test, y_test_pred)

# Calculate Cohen's Kappa

kappa_train = cohen_kappa_score(y_train, y_train_pred)

kappa_test = cohen_kappa_score(y_test, y_test_pred)

# Calculate 95% CI for AUC using Bootstrap

def calculate_auc_ci(y_true, y_pred_proba, n_bootstraps=1000, alpha=0.95):

rng = np.random.RandomState(42)

bootstrapped_scores = []

for _ in range(n_bootstraps):

# Resample the data and reset the index

indices = rng.randint(0, len(y_pred_proba), len(y_pred_proba))

y_true_resampled = y_true.iloc[indices].reset_index(drop=True)

y_pred_proba_resampled = y_pred_proba[indices]

if len(np.unique(y_true_resampled)) < 2:

continue

score = roc_auc_score(y_true_resampled, y_pred_proba_resampled)

bootstrapped_scores.append(score)

sorted_scores = np.array(bootstrapped_scores)

sorted_scores.sort()

lower = sorted_scores[int((1.0 - alpha) / 2.0 * len(sorted_scores))]

upper = sorted_scores[int((alpha + (1.0 - alpha) / 2.0) * len(sorted_scores))]

return lower, upper

ci_train = calculate_auc_ci(y_train, y_train_pred_proba)

ci_test = calculate_auc_ci(y_test, y_test_pred_proba)

# Function to calculate 95% CI for any metric

def calculate_metric_ci(metric_values, alpha=0.95):

sorted_scores = np.array(metric_values)

sorted_scores.sort()

lower = sorted_scores[int((1.0 - alpha) / 2.0 * len(sorted_scores))]

upper = sorted_scores[int((alpha + (1.0 - alpha) / 2.0) * len(sorted_scores))]

return lower, upper

# Bootstrap internal validation

n_bootstraps = 1000

bootstrapped_auc_scores = []

bootstrapped_accuracy_scores = []

bootstrapped_sensitivity_scores = []

bootstrapped_specificity_scores = []

bootstrapped_precision_scores = []

bootstrapped_f1_scores = []

example_fpr = None

example_tpr = None

for i in range(n_bootstraps):

X_train_resampled, y_train_resampled = resample(X_train, y_train)

y_train_pred_proba_resampled = predict_proba(X_train_resampled)

fpr_resampled, tpr_resampled, thresholds_resampled = roc_curve(y_train_resampled, y_train_pred_proba_resampled)

auc_score_resampled = auc(fpr_resampled, tpr_resampled)

optimal_idx_resampled = np.argmax(tpr_resampled - fpr_resampled)

optimal_threshold_resampled = thresholds_resampled[optimal_idx_resampled]

y_train_pred_resampled = (y_train_pred_proba_resampled > optimal_threshold_resampled).astype(int)

bootstrapped_auc_scores.append(auc_score_resampled)

bootstrapped_accuracy_scores.append(accuracy_score(y_train_resampled, y_train_pred_resampled))

bootstrapped_sensitivity_scores.append(recall_score(y_train_resampled, y_train_pred_resampled))

bootstrapped_specificity_scores.append(1 - fpr_resampled[optimal_idx_resampled])

bootstrapped_precision_scores.append(precision_score(y_train_resampled, y_train_pred_resampled))

bootstrapped_f1_scores.append(f1_score(y_train_resampled, y_train_pred_resampled))

if i == 0:

example_fpr = fpr_resampled

example_tpr = tpr_resampled

mean_bootstrap_auc = np.mean(bootstrapped_auc_scores)

ci_bootstrap_auc = calculate_metric_ci(bootstrapped_auc_scores)

mean_bootstrap_accuracy = np.mean(bootstrapped_accuracy_scores)

ci_bootstrap_accuracy = calculate_metric_ci(bootstrapped_accuracy_scores)

mean_bootstrap_sensitivity = np.mean(bootstrapped_sensitivity_scores)

ci_bootstrap_sensitivity = calculate_metric_ci(bootstrapped_sensitivity_scores)

mean_bootstrap_specificity = np.mean(bootstrapped_specificity_scores)

ci_bootstrap_specificity = calculate_metric_ci(bootstrapped_specificity_scores)

mean_bootstrap_precision = np.mean(bootstrapped_precision_scores)

ci_bootstrap_precision = calculate_metric_ci(bootstrapped_precision_scores)

mean_bootstrap_f1 = np.mean(bootstrapped_f1_scores)

ci_bootstrap_f1 = calculate_metric_ci(bootstrapped_f1_scores)

apparent_performance = auc_score_train

optimism = mean_bootstrap_auc - auc_score_train

corrected_performance = auc_score_train - optimism

# Bias-corrected performance

bias_corrected_performance = auc_score_train - (mean_bootstrap_auc - auc_score_train)

# Output results

print('**Training Data**')

print('Sensitivity:', sensitivity_train)

print('Specificity:', specificity_train)

print('Accuracy:', accuracy_train)

print('F1 Score:', f1_train)

print('Precision:', precision_train)

print('Recall:', recall_train)

print('AUC:', auc_score_train)

print('95% CI (AUC):', ci_train)

print('Log Loss:', log_loss_train)

print('MCC:', mcc_train)

print('Cohen\'s Kappa:', kappa_train)

print('**Test Data**')

print('Sensitivity:', sensitivity_test)

print('Specificity:', specificity_test)

print('Accuracy:', accuracy_test)

print('F1 Score:', f1_test)

print('Precision:', precision_test)

print('Recall:', recall_test)

print('AUC:', auc_score_test)

print('95% CI (AUC):', ci_test)

print('Log Loss:', log_loss_test)

print('MCC:', mcc_test)

print('Cohen\'s Kappa:', kappa_test)

print('**Bootstrap Results**')

print('Bootstrap Mean AUC:', mean_bootstrap_auc)

print('Bootstrap 95% CI (AUC):', ci_bootstrap_auc)

print('Bootstrap Mean Accuracy:', mean_bootstrap_accuracy)

print('Bootstrap 95% CI (Accuracy):', ci_bootstrap_accuracy)

print('Bootstrap Mean Sensitivity:', mean_bootstrap_sensitivity)

print('Bootstrap 95% CI (Sensitivity):', ci_bootstrap_sensitivity)

print('Bootstrap Mean Specificity:', mean_bootstrap_specificity)

print('Bootstrap 95% CI (Specificity):', ci_bootstrap_specificity)

print('Bootstrap Mean Precision:', mean_bootstrap_precision)

print('Bootstrap 95% CI (Precision):', ci_bootstrap_precision)

print('Bootstrap Mean F1 Score:', mean_bootstrap_f1)

print('Bootstrap 95% CI (F1 Score):', ci_bootstrap_f1)

print('Apparent performance:', apparent_performance)

print('Optimism:', optimism)

print('Corrected performance:', corrected_performance)

print('Bias-corrected performance (AUC):', bias_corrected_performance) # ★追加部分

# Plot ROC curve

plt.figure(figsize=(8, 8))

plt.plot(fpr_train, tpr_train, 'k-', label='Training AUC = %0.3f' % auc_score_train)

plt.plot(fpr_test, tpr_test, 'k--', label='Validation AUC = %0.3f' % auc_score_test)

plt.xlabel('1 - specificity')

plt.ylabel('Sensitivity')

plt.title('ROC Curve')

plt.legend(loc='lower right')

plt.gca().set_aspect('equal', adjustable='box')

plt.show()

# Plot Bootstrap AUC distribution

plt.figure(figsize=(8, 6)) # Set a rectangular figure size for better display

plt.hist(bootstrapped_auc_scores, bins=50, color='lightgrey', edgecolor='k', alpha=0.7)

plt.xlabel('AUC')

plt.ylabel('Frequency')

plt.title('Bootstrap AUC Distribution')

plt.axvline(x=mean_bootstrap_auc, color='k', linestyle='--', label='Mean AUC = %0.3f' % mean_bootstrap_auc)

plt.legend(loc='lower right')

plt.xlim(0.7, 1.0) # Set the x-axis limits to a reasonable range for AUC values

plt.show()

# Plot Calibration plot (Training and Test data)

plt.figure(figsize=(8, 8))

prob_true_train, prob_pred_train = calibration_curve(y_train, y_train_pred_proba, n_bins=10)

prob_true_test, prob_pred_test = calibration_curve(y_test, y_test_pred_proba, n_bins=10)

# Calculate and plot regression lines

slope_train, intercept_train, r_value_train, p_value_train, std_err_train = stats.linregress(prob_pred_train, prob_true_train)

slope_test, intercept_test, r_value_test, p_value_test, std_err_test = stats.linregress(prob_pred_test, prob_true_test)

plt.plot(prob_pred_train, prob_true_train, 'ko', label='Training Data')

plt.plot(prob_pred_test, prob_true_test, 'k^', label='Validation Data')

plt.plot([0, 1], [0, 1], 'k:', linewidth=1, label='Perfect calibration')

plt.plot(prob_pred_train, intercept_train + slope_train * np.array(prob_pred_train), 'k-', label=f'Train fit (slope={slope_train:.3f}, intercept={intercept_train:.3f})')

plt.plot(prob_pred_test, intercept_test + slope_test * np.array(prob_pred_test), 'k--', label=f'Validation fit (slope={slope_test:.3f}, intercept={intercept_test:.3f})')

plt.xlabel('Mean predicted probability')

plt.ylabel('Fraction of positives')

plt.title('Calibration Plot')

plt.legend(loc='lower right')

plt.gca().set_aspect('equal', adjustable='box')

plt.show()

# Output regression statistics

print('**Training Data Regression Statistics**')

print(f'Intercept: {intercept_train}')

print(f'Slope: {slope_train}')

print(f'R-squared: {r_value_train**2}')

print(f'P-value: {p_value_train}')

print(f'Standard Error: {std_err_train}')

print('**Validation Data Regression Statistics**')

print(f'Intercept: {intercept_test}')

print(f'Slope: {slope_test}')

print(f'R-squared: {r_value_test**2}')

print(f'P-value: {p_value_test}')

print(f'Standard Error: {std_err_test}')

# Plot Bootstrap ROC curve (example of the first bootstrap sample)

plt.figure(figsize=(10, 6))

plt.plot(example_fpr, example_tpr, 'k-', label='Bootstrap Sample ROC Curve')

plt.xlabel('1 - specificity')

plt.ylabel('Sensitivity')

plt.title('Bootstrap ROC Curve Example')

plt.legend(loc='lower right')

plt.gca().set_aspect('equal', adjustable='box')

plt.show()

# Output binning range and number of bins

bins = np.linspace(0, 1, 11)

print('Binning range:', bins)

print('Number of bins:', len(bins) - 1)

# Output actual observed probabilities in each bin (Training data)

print('**Binning information for Training Data**')

for i in range(len(prob_true_train)):

print(f'Bin {i+1}: Predicted probability range = [{bins[i]}, {bins[i+1]}], Observed probability = {prob_true_train[i]}')

# Output actual observed probabilities in each bin (Test data)

print('**Binning information for Test Data**')

for i in range(len(prob_true_test)):

print(f'Bin {i+1}: Predicted probability range = [{bins[i]}, {bins[i+1]}], Observed probability = {prob_true_test[i]}')

"""

**Title: Prediction One ^TM^ Predictive Model Evaluation Using ROC and Calibration plots**

Description:

This Python script evaluates the performance of predictive models using ROC curves, calibration plots, and various statistical metrics such as AUC, sensitivity, specificity, and more. It compares the training and validation datasets by calculating key metrics and plotting the results. The script also includes calculation of 95% confidence intervals for the AUC and regression statistics for calibration plots.

"""

**README**

**Overview**

This Python program evaluates the performance of a logistic regression model using the training and validation datasets. It calculates various performance metrics, including AUC, accuracy, sensitivity, and specificity. The program also generates ROC and calibration plots, ensuring that both plots maintain a square aspect ratio for clear visualization. Additionally, the program calculates confidence intervals for AUC and provides regression statistics for calibration.

**Requirements**

- Python 3.x
- Required libraries: pandas, numpy, scikit-learn, matplotlib, scipy

Install the necessary libraries using:

pip install pandas numpy scikit-learn matplotlib scipy

**How to Use**

1. **Prepare Data:**
   - Place your training and validation datasets in CSV format.
   - Ensure that the target variable and predicted probabilities are correctly referenced:
     - **Training Data (G1_Prediction_Result.csv)**:
       - Target variable: Column 6 (0-indexed as 5)
       - Predicted probabilities: Column 2 (0-indexed as 1)
     - **Validation Data (G2_Prediction_Result.csv)**:
       - Target variable: Column 14 (0-indexed as 13)
       - Predicted probabilities: Column 4 (0-indexed as 3)
2. **Modify File Paths:**
   - Update the file paths in the script to point to your datasets:

df_train = pd.read_csv("path/to/G1_Prediction_Result.csv")

df_test = pd.read_csv("path/to/G2_Prediction_Result.csv")

1. **Run the Script:**
   - Execute the script using Python:

python script_name.py

- - The program will:
    - Calculate performance metrics for both the training and validation datasets.
    - Generate and display ROC and calibration plots, ensuring they are square in shape.
    - Output AUC confidence intervals and calibration regression statistics.

1. **Interpretation:**
   - **ROC Curve:** Provides a visual comparison of the model's discrimination ability in both datasets.
   - **Calibration Plot:** Assesses the agreement between predicted probabilities and observed outcomes.
   - **Performance Metrics:** Metrics, such as sensitivity, specificity, accuracy, F1 score, and AUC, along with their confidence intervals, are displayed for both datasets.
   - **Regression Statistics:** Output includes slope, intercept, and correlation coefficients, which are important for understanding the calibration of the model.
2. **Output:**
   - **ROC Curve Plot:** A plot comparing the ROC curves for the training and validation datasets with AUC values.
   - **Calibration Plot:** A plot showing the calibration curve for both datasets, including regression lines.
   - **Performance Metrics:** Printed in the console, including sensitivity, specificity, accuracy, F1 score, AUC, log loss, MCC, and Cohen’s Kappa, with AUC confidence intervals.
   - **Calibration Regression Statistics:** Printed in the console, providing detailed calibration information for both the training and validation datasets.
3. **Customization:**
   - The script can be modified to reference different columns for the target variable and predicted probabilities as needed.

**Example**

For demonstration purposes, the script uses generalized file paths. Replace these with the actual paths to your datasets to perform the analysis.

# Importing necessary libraries

import pandas as pd

import numpy as np

from sklearn.metrics import (roc_curve, auc, accuracy_score, f1_score, precision_score, recall_score,

confusion_matrix, roc_auc_score, log_loss, matthews_corrcoef, cohen_kappa_score)

from sklearn.calibration import calibration_curve

import matplotlib.pyplot as plt

from sklearn.linear_model import LinearRegression

from scipy.stats import linregress

# Load data

df_train = pd.read_csv("path/to/G1_Prediction_Result.csv") # Generalized file path

df_test = pd.read_csv("path/to/G2_Prediction_Result.csv") # Generalized file path

# Define target variable and predicted probabilities

y_train = df_train.iloc[:, 5] # Target variable

y_train_pred_proba = df_train.iloc[:, 1] # Predicted probabilities

y_test = df_test.iloc[:, 13] # Target variable

y_test_pred_proba = df_test.iloc[:, 3] # Predicted probabilities

# ROC curve and AUC for training data

fpr_train, tpr_train, thresholds_train = roc_curve(y_train, y_train_pred_proba)

auc_score_train = auc(fpr_train, tpr_train)

# ROC curve and AUC for validation data

fpr_test, tpr_test, thresholds_test = roc_curve(y_test, y_test_pred_proba)

auc_score_test = auc(fpr_test, tpr_test)

# Calculate evaluation metrics

optimal_idx_train = np.argmax(tpr_train - fpr_train)

optimal_threshold_train = thresholds_train[optimal_idx_train]

y_train_pred = (y_train_pred_proba >= optimal_threshold_train).astype(int)

sensitivity_train = tpr_train[optimal_idx_train]

specificity_train = 1 - fpr_train[optimal_idx_train]

accuracy_train = accuracy_score(y_train, y_train_pred)

f1_train = f1_score(y_train, y_train_pred)

precision_train = precision_score(y_train, y_train_pred)

recall_train = recall_score(y_train, y_train_pred)

log_loss_train = log_loss(y_train, y_train_pred_proba)

mcc_train = matthews_corrcoef(y_train, y_train_pred)

kappa_train = cohen_kappa_score(y_train, y_train_pred)

optimal_idx_test = np.argmax(tpr_test - fpr_test)

optimal_threshold_test = thresholds_test[optimal_idx_test]

y_test_pred = (y_test_pred_proba >= optimal_threshold_test).astype(int)

sensitivity_test = tpr_test[optimal_idx_test]

specificity_test = 1 - fpr_test[optimal_idx_test]

accuracy_test = accuracy_score(y_test, y_test_pred)

f1_test = f1_score(y_test, y_test_pred)

precision_test = precision_score(y_test, y_test_pred)

recall_test = recall_score(y_test, y_test_pred)

log_loss_test = log_loss(y_test, y_test_pred_proba)

mcc_test = matthews_corrcoef(y_test, y_test_pred)

kappa_test = cohen_kappa_score(y_test, y_test_pred)

# Calculate AUC confidence intervals

def calculate_auc_ci(auc, y_true, y_pred_proba, confidence=0.95):

n1 = sum(y_true)

n2 = len(y_true) - n1

q1 = auc / (2 - auc)

q2 = 2 * auc**2 / (1 + auc)

se_auc = np.sqrt((auc * (1 - auc) + (n1 - 1) * (q1 - auc**2) + (n2 - 1) * (q2 - auc**2)) / (n1 * n2))

lower = auc - 1.96 * se_auc

upper = auc + 1.96 * se_auc

return lower, upper

ci_train = calculate_auc_ci(auc_score_train, y_train, y_train_pred_proba)

ci_test = calculate_auc_ci(auc_score_test, y_test, y_test_pred_proba)

# Output results

print('**Training Data**')

print('Sensitivity:', sensitivity_train)

print('Specificity:', specificity_train)

print('Accuracy:', accuracy_train)

print('F1 Score:', f1_train)

print('Precision:', precision_train)

print('Recall:', recall_train)

print('Log Loss:', log_loss_train)

print('MCC:', mcc_train)

print('Cohen’s Kappa:', kappa_train)

print('AUC:', auc_score_train)

print('95% CI (AUC):', ci_train)

print('**Validation Data**')

print('Sensitivity:', sensitivity_test)

print('Specificity:', specificity_test)

print('Accuracy:', accuracy_test)

print('F1 Score:', f1_test)

print('Precision:', precision_test)

print('Recall:', recall_test)

print('Log Loss:', log_loss_test)

print('MCC:', mcc_test)

print('Cohen’s Kappa:', kappa_test)

print('AUC:', auc_score_test)

print('95% CI (AUC):', ci_test)

# Plot ROC curve with a square aspect ratio

plt.figure(figsize=(8, 8)) # Set the figure to be square

plt.plot(fpr_train, tpr_train, 'k-', label='Training AUC = %0.3f' % auc_score_train) # Solid black line

plt.plot(fpr_test, tpr_test, 'k--', label='Validation AUC = %0.3f' % auc_score_test) # Dashed black line

plt.xlabel('1 - Specificity')

plt.ylabel('Sensitivity')

plt.title('ROC Curve')

plt.legend(loc='lower right')

plt.axis('square') # Ensure the plot is square

plt.show()

# Calibration plot with regression lines and a square aspect ratio

def plot_calibration_curve(y_true, y_pred_proba, label, marker, linestyle, line_label):

prob_true, prob_pred = calibration_curve(y_true, y_pred_proba, n_bins=10)

plt.plot(prob_pred, prob_true, marker, label=label)

slope, intercept, r_value, p_value, std_err = linregress(prob_pred, prob_true)

line = slope * np.array(prob_pred) + intercept

plt.plot(prob_pred, line, linestyle, label=f'{line_label} (slope={slope:.3f}, intercept={intercept:.3f})')

return slope, intercept, r_value, p_value, std_err

plt.figure(figsize=(8, 8)) # Make the plot square

train_stats = plot_calibration_curve(y_train, y_train_pred_proba, 'Training Data', 'ko', 'k-', 'Train fit')

test_stats = plot_calibration_curve(y_test, y_test_pred_proba, 'Validation Data', 'k^', 'k--', 'Validation fit')

plt.plot([0, 1], [0, 1], 'k:', label='Perfect calibration')

plt.xlabel('Mean Predicted Probability')

plt.ylabel('Fraction of Positives')

plt.title('Calibration Plot')

plt.axis('square') # Ensure the plot is square

plt.legend(loc='lower right')

plt.show()

# Output regression statistics

print('**Training Data Regression Statistics**')

print('Slope:', train_stats[0])

print('Intercept:', train_stats[1])

print('Correlation coefficient:', train_stats[2])

print('Determination coefficient:', train_stats[2]**2)

print('p-value:', train_stats[3])

print('Standard error:', train_stats[4])

print('**Validation Data Regression Statistics**')

print('Slope:', test_stats[0])

print('Intercept:', test_stats[1])

print('Correlation coefficient:', test_stats[2])

print('Determination coefficient:', test_stats[2]**2)

print('p-value:', test_stats[3])

print('Standard error:', test_stats[4])

"""

**Title: Comparative Machine Learning Model Evaluation with SHAP Analysis for Predicting Outcomes using D-dimer Data**

Description:

This Python script compares the performance of three machine learning models: LightGBM, XGBoost, and CatBoost, using D-dimer data from 2018 to 2019 (training) and 2020 to 2021 (validation) to predict patient outcomes.

The models are evaluated based on AUROC, sensitivity, specificity, accuracy, F1 score, log loss, MCC,

Cohen's kappa, precision-recall AUC, and more. The script includes SHAP analysis for feature importance,

and calibration plots to assess the reliability of probability predictions.

"""

**README**

**Overview**

This Python program evaluates the performance of three machine learning models—LightGBM, XGBoost, and CatBoost—using the training and validation datasets. It calculates various performance metrics, including AUROC, accuracy, sensitivity, specificity, and more. Additionally, it generates ROC and calibration plots, conducts SHAP analysis to explain model predictions, and provides regression statistics for calibration. The program outputs the results in a structured format, facilitating comparisons across models and datasets.

**Requirements**

- Python 3.x
- Required libraries: pandas, numpy, matplotlib, shap, xgboost, lightgbm, catboost, scikit-learn, scipy

Install the necessary libraries using:

pip install pandas numpy matplotlib shap xgboost lightgbm catboost scikit-learn scipy

**How to Use**

1. **Prepare Data:**
   - Place your training and validation datasets in CSV format.
   - Ensure that the feature columns and target variable (Outcome) are correctly referenced:
     - Feature columns: "Sex":"Ferritin"
     - Target variable: "Outcome"
2. **Modify File Paths:**
   - Update the file paths in the script to point to your datasets:

train_data = pd.read_csv("path/to/your/training_data.csv", encoding="utf-8")

test_data = pd.read_csv("path/to/your/validation_data.csv", encoding="utf-8")

1. **Run the Script:**
   - Execute the script using Python:

python script_name.py

- - The program will:
    - Train the LightGBM, XGBoost, and CatBoost models on the training dataset.
    - Evaluate each model on both the training and validation datasets using multiple metrics.
    - Generate and display ROC and calibration plots, ensuring they are square in shape.
    - Conduct SHAP analysis to explain feature importance for each model.
    - Output regression statistics and other performance metrics in a structured format.

1. **Interpretation:**
   - **ROC Curve:** Visualizes the model's discrimination ability on both datasets, showing the area under the curve (AUC).
   - **Calibration Plot:** Assesses the agreement between predicted probabilities and observed outcomes, with regression lines provided for deeper analysis.
   - **SHAP Analysis:** Explains the contribution of each feature to the model's predictions, helping to understand the model's behavior.
   - **Performance Metrics:** Metrics such as sensitivity, specificity, accuracy, F1 score, AUROC, log loss, MCC, Cohen’s Kappa, precision, recall, and precision-recall AUC are displayed for both datasets, enabling comprehensive evaluation.
2. **Output:**
   - **ROC Curve Plots:** Separate ROC curves for each model, comparing the training and validation datasets.
   - **Calibration Plot:** Separate calibration plots for each model, including regression statistics for both the training and validation datasets.
   - **SHAP Values:** SHAP analysis outputs for each model, detailing feature importance.
   - **Performance Metrics:** Printed in the console, structured in a DataFrame for easy comparison across models and datasets.
   - **Regression Statistics:** Detailed calibration regression statistics for both the training and validation datasets.
3. **Customization:**
   - The script can be modified to include additional models or change hyperparameters for each model.
   - Adjustments to the SHAP analysis can be made to focus on specific features or datasets.

**Example**

For demonstration purposes, the script uses generalized file paths. Replace these with the actual paths to your datasets to perform the analysis.

# Install required libraries

!pip install shap

!pip install xgboost

!pip install lightgbm

!pip install catboost

!pip install scikit-learn

import pandas as pd

import numpy as np

import matplotlib.pyplot as plt

import shap

from xgboost import XGBClassifier

import lightgbm as lgb

from catboost import CatBoostClassifier

from sklearn.metrics import (roc_auc_score, roc_curve, accuracy_score, f1_score, log_loss,

matthews_corrcoef, cohen_kappa_score, precision_recall_curve,

average_precision_score, precision_score, recall_score)

from sklearn.calibration import calibration_curve

from scipy.stats import linregress

# Load training data

train_data = pd.read_csv("path/to/your/training_data.csv", encoding="utf-8") # Generalized file path

# Load validation data

test_data = pd.read_csv("path/to/your/validation_data.csv", encoding="utf-8") # Generalized file path

# Replace spaces in feature names with underscores

train_data.columns = train_data.columns.str.replace(' ', '_')

test_data.columns = test_data.columns.str.replace(' ', '_')

# Select features and target variable

X_train = train_data.loc[:, "Sex":"Ferritin"]

y_train = train_data["Outcome"]

X_test = test_data.loc[:, "Sex":"Ferritin"]

y_test = test_data["Outcome"]

# Define models (in order) These are the definitions of the hyperparameter.

models = {

"LightGBM": lgb.LGBMClassifier(max_depth=3, learning_rate=0.1, n_estimators=100, subsample=0.8, colsample_bytree=0.8, force_col_wise=True),

"XGBoost": XGBClassifier(eval_metric='logloss', max_depth=3, eta=0.1, min_child_weight=1, subsample=0.8, colsample_bytree=0.8),

"CatBoost": CatBoostClassifier(depth=3, learning_rate=0.1, iterations=100, auto_class_weights='Balanced', verbose=0)

}

# Color map (for line styles)

train_linestyle = '-'

validation_linestyle = '--'

line_color = 'black'

# Create a dataframe for statistical indicators

results = pd.DataFrame(columns=["Model", "Dataset", "AUROC", "Sensitivity", "Specificity", "Accuracy", "F1 Score", "Log Loss", "MCC", "Cohen's Kappa", "Precision-Recall AUC", "Precision", "Recall"])

for name, model in models.items():

# Train the model

model.fit(X_train, y_train)

# Calculate predicted probabilities

y_train_pred_proba = model.predict_proba(X_train)[:, 1]

y_test_pred_proba = model.predict_proba(X_test)[:, 1]

# Calculate ROC curve

fpr_train, tpr_train, thresholds_train = roc_curve(y_train, y_train_pred_proba)

fpr_test, tpr_test, thresholds_test = roc_curve(y_test, y_test_pred_proba)

# Calculate AUROC

auc_train = roc_auc_score(y_train, y_train_pred_proba)

auc_test = roc_auc_score(y_test, y_test_pred_proba)

# Determine the optimal threshold and calculate sensitivity and specificity

optimal_idx_train = np.argmax(tpr_train - fpr_train)

optimal_threshold_train = thresholds_train[optimal_idx_train]

sensitivity_train = tpr_train[optimal_idx_train]

specificity_train = 1 - fpr_train[optimal_idx_train]

optimal_idx_test = np.argmax(tpr_test - fpr_test)

optimal_threshold_test = thresholds_test[optimal_idx_test]

sensitivity_test = tpr_test[optimal_idx_test]

specificity_test = 1 - fpr_test[optimal_idx_test]

# Calculate other metrics

accuracy_train = accuracy_score(y_train, (y_train_pred_proba >= optimal_threshold_train).astype(int))

f1_train = f1_score(y_train, (y_train_pred_proba >= optimal_threshold_train).astype(int))

log_loss_train = log_loss(y_train, y_train_pred_proba)

mcc_train = matthews_corrcoef(y_train, (y_train_pred_proba >= optimal_threshold_train).astype(int))

kappa_train = cohen_kappa_score(y_train, (y_train_pred_proba >= optimal_threshold_train).astype(int))

precision_train = precision_score(y_train, (y_train_pred_proba >= optimal_threshold_train).astype(int))

recall_train = recall_score(y_train, (y_train_pred_proba >= optimal_threshold_train).astype(int))

pr_auc_train = average_precision_score(y_train, y_train_pred_proba)

accuracy_test = accuracy_score(y_test, (y_test_pred_proba >= optimal_threshold_test).astype(int))

f1_test = f1_score(y_test, (y_test_pred_proba >= optimal_threshold_test).astype(int))

log_loss_test = log_loss(y_test, y_test_pred_proba)

mcc_test = matthews_corrcoef(y_test, (y_test_pred_proba >= optimal_threshold_test).astype(int))

kappa_test = cohen_kappa_score(y_test, (y_test_pred_proba >= optimal_threshold_test).astype(int))

precision_test = precision_score(y_test, (y_test_pred_proba >= optimal_threshold_test).astype(int))

recall_test = recall_score(y_test, (y_test_pred_proba >= optimal_threshold_test).astype(int))

pr_auc_test = average_precision_score(y_test, y_test_pred_proba)

# Add results to dataframe

results = pd.concat([results, pd.DataFrame({

"Model": [name],

"Dataset": ["Training"],

"AUROC": [auc_train],

"Sensitivity": [sensitivity_train],

"Specificity": [specificity_train],

"Accuracy": [accuracy_train],

"F1 Score": [f1_train],

"Log Loss": [log_loss_train],

"MCC": [mcc_train],

"Cohen's Kappa": [kappa_train],

"Precision-Recall AUC": [pr_auc_train],

"Precision": [precision_train],

"Recall": [recall_train]

})])

results = pd.concat([results, pd.DataFrame({

"Model": [name],

"Dataset": ["Validation"],

"AUROC": [auc_test],

"Sensitivity": [sensitivity_test],

"Specificity": [specificity_test],

"Accuracy": [accuracy_test],

"F1 Score": [f1_test],

"Log Loss": [log_loss_test],

"MCC": [mcc_test],

"Cohen's Kappa": [kappa_test],

"Precision-Recall AUC": [pr_auc_test],

"Precision": [precision_test],

"Recall": [recall_test]

})])

# Plot ROC curve for each model separately

plt.figure(figsize=(8, 8)) # Make the plot square

plt.plot(fpr_train, tpr_train, label=f'Training ROC curve (AUC = {auc_train:.3f})', color=line_color, linestyle=train_linestyle)

plt.plot(fpr_test, tpr_test, label=f'Validation ROC curve (AUC = {auc_test:.3f})', color=line_color, linestyle=validation_linestyle)

plt.xlim([0.0, 1.0])

plt.ylim([0.0, 1.0])

plt.xlabel('1 - Specificity')

plt.ylabel('Sensitivity')

plt.title(f'ROC Curve - {name}')

plt.legend(loc="lower right")

plt.gca().set_aspect('equal', adjustable='box')

plt.show()

# SHAP analysis

explainer = shap.Explainer(model, X_train)

shap_values = explainer(X_train)

shap_values_dict[name] = shap_values

explainer_dict[name] = explainer

# Plot Calibration Curve for CatBoost with Legend in Top Left

if name == "CatBoost":

plt.figure(figsize=(8, 8)) # Make the plot square

y_train_pred_proba = model.predict_proba(X_train)[:, 1]

y_test_pred_proba = model.predict_proba(X_test)[:, 1]

def plot_calibration_curve(y_true, y_pred_proba, label, color, linestyle, marker):

prob_true, prob_pred = calibration_curve(y_true, y_pred_proba, n_bins=10)

plt.plot(prob_pred, prob_true, marker=marker, linestyle='none', color=color, label=label)

slope, intercept, r_value, p_value, std_err = linregress(prob_pred, prob_true)

line = slope * np.array(prob_pred) + intercept

plt.plot(prob_pred, line, linestyle, color=color, label=f'{label} fit (slope={slope:.3f}, intercept={intercept:.3f})')

return slope, intercept, r_value, p_value, std_err

plot_calibration_curve(y_train, y_train_pred_proba, f'Training CatBoost', line_color, train_linestyle, 'o')

plot_calibration_curve(y_test, y_test_pred_proba, f'Validation CatBoost', line_color, validation_linestyle, '^')

plt.plot([0, 1], [0, 1], 'k:', label='Perfect calibration')

plt.xlim([0.0, 1.0])

plt.ylim([0.0, 1.0])

plt.xlabel('Mean Predicted Probability')

plt.ylabel('Fraction of Positives')

plt.title('Calibration Plot - CatBoost')

plt.legend(loc="upper left") # Move legend to the top left corner

plt.gca().set_aspect('equal', adjustable='box')

plt.show()

else:

# Plot Calibration Curve for other models

plt.figure(figsize=(8, 8)) # Make the plot square

y_train_pred_proba = model.predict_proba(X_train)[:, 1]

y_test_pred_proba = model.predict_proba(X_test)[:, 1]

def plot_calibration_curve(y_true, y_pred_proba, label, color, linestyle, marker):

prob_true, prob_pred = calibration_curve(y_true, y_pred_proba, n_bins=10)

plt.plot(prob_pred, prob_true, marker=marker, linestyle='none', color=color, label=label)

slope, intercept, r_value, p_value, std_err = linregress(prob_pred, prob_true)

line = slope * np.array(prob_pred) + intercept

plt.plot(prob_pred, line, linestyle, color=color, label=f'{label} fit (slope={slope:.3f}, intercept={intercept:.3f})')

return slope, intercept, r_value, p_value, std_err

plot_calibration_curve(y_train, y_train_pred_proba, f'Training {name}', line_color, train_linestyle, 'o')

plot_calibration_curve(y_test, y_test_pred_proba, f'Validation {name}', line_color, validation_linestyle, '^')

plt.plot([0, 1], [0, 1], 'k:', label='Perfect calibration')

plt.xlim([0.0, 1.0])

plt.ylim([0.0, 1.0])

plt.xlabel('Mean Predicted Probability')

plt.ylabel('Fraction of Positives')

plt.title(f'Calibration Plot - {name}')

plt.legend(loc="lower right") # Default legend location

plt.gca().set_aspect('equal', adjustable='box')

plt.show()

# Output regression statistics

for name, model in models.items():

print(f'**{name} Training Data Regression Statistics**')

y_train_pred_proba = model.predict_proba(X_train)[:, 1]

prob_true, prob_pred = calibration_curve(y_train, y_train_pred_proba, n_bins=10)

slope, intercept, r_value, p_value, std_err = linregress(prob_pred, prob_true)

print('Slope:', slope)

print('Intercept:', intercept)

print('Correlation coefficient:', r_value)

print('Determination coefficient:', r_value**2)

print('p-value:', p_value)

print('Standard error:', std_err)

print(f'**{name} Validation Data Regression Statistics**')

y_test_pred_proba = model.predict_proba(X_test)[:, 1]

prob_true, prob_pred = calibration_curve(y_test, y_test_pred_proba, n_bins=10)

slope, intercept, r_value, p_value, std_err = linregress(prob_pred, prob_true)

print('Slope:', slope)

print('Intercept:', intercept)

print('Correlation coefficient:', r_value)

print('Determination coefficient:', r_value**2)

print('p-value:', p_value)

print('Standard error:', std_err)

# Display statistical indicators

results = results.sort_values(by=["Model", "Dataset"], key=lambda x: x.map({"LightGBM": 0, "XGBoost": 1, "CatBoost": 2}))

print(results)

"""

**Title: Comparative Machine Learning Model Evaluation with SHAP Analysis, ROC, and Calibration Plots for Predicting Outcomes using D-dimer Data**

Description:

This Python script evaluates three machine learning models—LightGBM, XGBoost, and CatBoost—using D-dimer data to predict patient outcomes. The models are trained and validated on separate datasets, and performance metrics such as AUROC, sensitivity, specificity, accuracy, F1 score, log loss, MCC, Cohen's kappa, and precision-recall AUC are calculated for both the training and validation datasets. Additionally, calibration curves and SHapley Additive exPlanations (SHAP) analyses are performed to assess model reliability and interpretability. The script outputs high-quality visualizations and statistical summaries, making it suitable for inclusion as supplementary material in a major medical journal.

"""

**Overview**

This Python program evaluates the performance of three machine learning models—LightGBM, XGBoost, and CatBoost—using the training and validation datasets. It calculates various performance metrics, including AUROC, accuracy, sensitivity, specificity, and more. Additionally, it generates ROC and calibration plots, conducts SHAP analysis to explain model predictions, and provides regression statistics for calibration.

**Requirements**

Python 3.x

Required libraries: shap, xgboost, lightgbm, catboost, pandas, numpy, matplotlib, scikit-learn, scipy

**How to Use**

1. **Prepare Data:**
   - Place your training and validation datasets in CSV format.
   - Ensure that the feature columns and target variable (Outcome) are correctly referenced:
     - Feature columns: "Sex":"Ferritin"
     - Target variable: "Outcome"
2. **Modify File Paths:**
   - Update the file paths in the script to point to your datasets:

train_data = pd.read_csv("path/to/your/training_data.csv", encoding="utf-8")

test_data = pd.read_csv("path/to/your/validation_data.csv", encoding="utf-8")

1. **Run the Script:**
   - Execute the script using Python:
   - The program will:
     - Train the LightGBM, XGBoost, and CatBoost models on the training dataset.
     - Evaluate each model on both training and validation datasets using multiple metrics.
     - Generate and display ROC and calibration plots, ensuring they are square in shape.
     - Conduct SHAP analysis to explain feature importance for each model.
     - Output regression statistics and other performance metrics in a structured format.
2. **Interpretation:**
   - **ROC Curve:** Provides a visual comparison of the model's discrimination ability on both datasets, showing the area under the curve (AUC).
   - **Calibration Plot:** Assesses the agreement between predicted probabilities and observed outcomes, with regression lines provided for deeper analysis.
   - **SHAP Analysis:** Explains the contribution of each feature to the model's predictions, helping to understand the model's behavior.
   - **Performance Metrics:** Metrics such as sensitivity, specificity, accuracy, F1 score, AUROC, log loss, MCC, Cohen’s Kappa, precision, recall, and precision-recall AUC are displayed for both datasets, enabling comprehensive evaluation.
3. **Output:**
   - **ROC Curve Plot:** A plot comparing the ROC curves for the training and validation datasets with AUC values.
   - **Calibration Plot:** A plot showing the calibration curve for both datasets, including regression lines.
   - **SHAP Values:** SHAP analysis outputs for each model, detailing feature importance.
   - **Performance Metrics:** Printed in the console, structured in a DataFrame for easy comparison across models and datasets.
   - **Regression Statistics:** Detailed calibration regression statistics for both training and validation datasets.
4. **Customization:**
   - The script can be modified to include additional models or change hyperparameters for each model.
   - Adjustments to the SHAP analysis can be made to focus on specific features or datasets.

# Install required libraries

!pip install shap

!pip install xgboost

!pip install lightgbm

!pip install catboost

!pip install scikit-learn

import pandas as pd

import numpy as np

import matplotlib.pyplot as plt

import shap

from xgboost import XGBClassifier

import lightgbm as lgb

from catboost import CatBoostClassifier

from sklearn.metrics import (roc_auc_score, roc_curve, accuracy_score, f1_score, log_loss,

matthews_corrcoef, cohen_kappa_score, precision_recall_curve,

average_precision_score, precision_score, recall_score)

from sklearn.calibration import calibration_curve

from scipy.stats import linregress

# Load training data

train_data = pd.read_csv("path/to/your/training_data.csv", encoding="utf-8") # Generalized file path

# Load validation data

test_data = pd.read_csv("path/to/your/validation_data.csv", encoding="utf-8") # Generalized file path

# Replace spaces in feature names with underscores

train_data.columns = train_data.columns.str.replace(' ', '_')

test_data.columns = test_data.columns.str.replace(' ', '_')

# Select features and target variable

X_train = train_data.loc[:, "Sex":"Ferritin"]

y_train = train_data["Outcome"]

X_test = test_data.loc[:, "Sex":"Ferritin"]

y_test = test_data["Outcome"]

# Define models (in order)

models = {

"LightGBM": lgb.LGBMClassifier(max_depth=3, learning_rate=0.1, n_estimators=100, subsample=0.8, colsample_bytree=0.8, force_col_wise=True),

"XGBoost": XGBClassifier(use_label_encoder=False, eval_metric='logloss', max_depth=3, eta=0.1, min_child_weight=1, subsample=0.8, colsample_bytree=0.8),

"CatBoost": CatBoostClassifier(depth=3, learning_rate=0.1, iterations=100, auto_class_weights='Balanced', verbose=0)

}

# Color map

colors = {

"LightGBM": "red",

"XGBoost": "black",

"CatBoost": "blue"

}

# Create a dataframe for statistical indicators

results = pd.DataFrame(columns=["Model", "Dataset", "AUROC", "AUC 95% CI Lower", "AUC 95% CI Upper", "Sensitivity", "Specificity", "Accuracy", "F1 Score", "Log Loss", "MCC", "Cohen's Kappa", "Precision-Recall AUC"])

plt.figure(figsize=(10, 10))

# Prepare for SHAP analysis

shap_values_dict = {}

explainer_dict = {}

for name, model in models.items():

# Train the model

model.fit(X_train, y_train)

# Calculate predicted probabilities

y_train_pred_proba = model.predict_proba(X_train)[:, 1]

y_test_pred_proba = model.predict_proba(X_test)[:, 1]

# Calculate ROC curve

fpr_train, tpr_train, thresholds_train = roc_curve(y_train, y_train_pred_proba)

fpr_test, tpr_test, thresholds_test = roc_curve(y_test, y_test_pred_proba)

# Calculate AUROC

auc_train = roc_auc_score(y_train, y_train_pred_proba)

auc_test = roc_auc_score(y_test, y_test_pred_proba)

# Calculate standard error of AUC

def auc_se(auc, y_true):

n1 = np.sum(y_true == 1)

n0 = np.sum(y_true == 0)

q1 = auc / (2 - auc)

q2 = 2 * auc**2 / (1 + auc)

se = np.sqrt((auc * (1 - auc) + (n1 - 1) * (q1 - auc**2) + (n0 - 1) * (q2 - auc**2)) / (n1 * n0))

return se

# Calculate 95% confidence interval of AUC

auc_train_se = auc_se(auc_train, y_train)

auc_train_ci_lower = auc_train - 1.96 * auc_train_se

auc_train_ci_upper = auc_train + 1.96 * auc_train_se

auc_test_se = auc_se(auc_test, y_test)

auc_test_ci_lower = auc_test - 1.96 * auc_test_se

auc_test_ci_upper = auc_test + 1.96 * auc_test_se

# Plot ROC curve

plt.plot(fpr_train, tpr_train, label=f'{name} Training ROC curve (AUC = {auc_train:.3f})', color=colors[name], linestyle='-')

plt.plot(fpr_test, tpr_test, label=f'{name} Validation ROC curve (AUC = {auc_test:.3f})', color=colors[name], linestyle='--')

# Calculate sensitivity and specificity at optimal threshold

optimal_idx_train = np.argmax(tpr_train - fpr_train)

sensitivity_train = tpr_train[optimal_idx_train]

specificity_train = 1 - fpr_train[optimal_idx_train]

optimal_idx_test = np.argmax(tpr_test - fpr_test)

sensitivity_test = tpr_test[optimal_idx_test]

specificity_test = 1 - fpr_test[optimal_idx_test]

# Function to calculate evaluation metrics

def calculate_metrics(y_true, y_pred_proba, threshold=0.5):

y_pred = (y_pred_proba >= threshold).astype(int)

accuracy = accuracy_score(y_true, y_pred)

f1 = f1_score(y_true, y_pred)

mcc = matthews_corrcoef(y_true, y_pred)

kappa = cohen_kappa_score(y_true, y_pred)

log_loss_value = log_loss(y_true, y_pred_proba)

precision = precision_score(y_true, y_pred)

recall = recall_score(y_true, y_pred)

return accuracy, f1, mcc, kappa, log_loss_value, precision, recall

# Calculate Precision-Recall AUC

precision_train, recall_train, _ = precision_recall_curve(y_train, y_train_pred_proba)

pr_auc_train = average_precision_score(y_train, y_train_pred_proba)

precision_test, recall_test, _ = precision_recall_curve(y_test, y_test_pred_proba)

pr_auc_test = average_precision_score(y_test, y_test_pred_proba)

# Evaluation metrics for training dataset

accuracy_train, f1_train, mcc_train, kappa_train, log_loss_train, precision_train, recall_train = calculate_metrics(y_train, y_train_pred_proba)

# Evaluation metrics for validation dataset

accuracy_test, f1_test, mcc_test, kappa_test, log_loss_test, precision_test, recall_test = calculate_metrics(y_test, y_test_pred_proba)

# Add statistical indicators to the dataframe

results = pd.concat([results, pd.DataFrame({

"Model": [name],

"Dataset": ["Training"],

"AUROC": [auc_train],

"AUC 95% CI Lower": [auc_train_ci_lower],

"AUC 95% CI Upper": [auc_train_ci_upper],

"Sensitivity": [sensitivity_train],

"Specificity": [specificity_train],

"Accuracy": [accuracy_train],

"F1 Score": [f1_train],

"Log Loss": [log_loss_train],

"MCC": [mcc_train],

"Cohen's Kappa": [kappa_train],

"Precision": [precision_train],

"Recall": [recall_train],

"Precision-Recall AUC": [pr_auc_train]

})])

results = pd.concat([results, pd.DataFrame({

"Model": [name],

"Dataset": ["Validation"],

"AUROC": [auc_test],

"AUC 95% CI Lower": [auc_test_ci_lower],

"AUC 95% CI Upper": [auc_test_ci_upper],

"Sensitivity": [sensitivity_test],

"Specificity": [specificity_test],

"Accuracy": [accuracy_test],

"F1 Score": [f1_test],

"Log Loss": [log_loss_test],

"MCC": [mcc_test],

"Cohen's Kappa": [kappa_test],

"Precision": [precision_test],

"Recall": [recall_test],

"Precision-Recall AUC": [pr_auc_test]

})])

# SHAP analysis

explainer = shap.Explainer(model, X_train)

shap_values = explainer(X_train)

shap_values_dict[name] = shap_values

explainer_dict[name] = explainer

plt.xlim([0.0, 1.0])

plt.ylim([0.0, 1.05])

plt.xlabel('1 - specificity')

plt.ylabel('Sensitivity')

plt.title('ROC Curve Comparison')

plt.legend(loc="lower right")

plt.show()

# Calibration plot with regression lines

plt.figure(figsize=(10, 10)) # Make the plot square

for name, model in models.items():

y_train_pred_proba = model.predict_proba(X_train)[:, 1]

y_test_pred_proba = model.predict_proba(X_test)[:, 1]

def plot_calibration_curve(y_true, y_pred_proba, label, color, linestyle, marker):

prob_true, prob_pred = calibration_curve(y_true, y_pred_proba, n_bins=10)

plt.plot(prob_pred, prob_true, marker=marker, linestyle='none', color=color, label=label)

slope, intercept, r_value, p_value, std_err = linregress(prob_pred, prob_true)

line = slope * np.array(prob_pred) + intercept

plt.plot(prob_pred, line, linestyle, color=color, label=f'{label} fit (slope={slope:.3f}, intercept={intercept:.3f})')

return slope, intercept, r_value, p_value, std_err

plot_calibration_curve(y_train, y_train_pred_proba, f'Training {name}', colors[name], '-', 'o')

plot_calibration_curve(y_test, y_test_pred_proba, f'Validation {name}', colors[name], '--', '^')

plt.plot([0, 1], [0, 1], 'k:', label='Perfect calibration')

plt.xlabel('Mean Predicted Probability')

plt.ylabel('Fraction of Positives')

plt.title('Calibration Plot')

# Adjust legend to be below the plot area

plt.legend(loc='upper center', bbox_to_anchor=(0.5, -0.15), ncol=2)

plt.tight_layout()

plt.show()

# Output regression statistics

for name, model in models.items():

print(f'**{name} Training Data Regression Statistics**')

y_train_pred_proba = model.predict_proba(X_train)[:, 1]

prob_true, prob_pred = calibration_curve(y_train, y_train_pred_proba, n_bins=10)

slope, intercept, r_value, p_value, std_err = linregress(prob_pred, prob_true)

print('Slope:', slope)

print('Intercept:', intercept)

print('Correlation coefficient:', r_value)

print('Determination coefficient:', r_value**2)

print('p-value:', p_value)

print('Standard error:', std_err)

print(f'**{name} Validation Data Regression Statistics**')

y_test_pred_proba = model.predict_proba(X_test)[:, 1]

prob_true, prob_pred = calibration_curve(y_test, y_test_pred_proba, n_bins=10)

slope, intercept, r_value, p_value, std_err = linregress(prob_pred, prob_true)

print('Slope:', slope)

print('Intercept:', intercept)

print('Correlation coefficient:', r_value)

print('Determination coefficient:', r_value**2)

print('p-value:', p_value)

print('Standard error:', std_err)

# Display statistical indicators

results = results.sort_values(by=["Model", "Dataset"], key=lambda x: x.map({"LightGBM": 0, "XGBoost": 1, "CatBoost": 2}))

print(results)

"""

**Title: Bootstrap Evaluation of Machine Learning Models for Predicting Outcomes using D-dimer Data**

Description:

This Python script evaluates the performance of three machine learning models: LightGBM, XGBoost, and CatBoost, using bootstrap resampling to assess the robustness and reliability of the models. The models are trained on D-dimer data and evaluated on key metrics such as AUROC, accuracy, sensitivity, specificity, precision, and F1 score. The script produces bootstrap AUC distributions and calculates confidence intervals for the metrics, along with bias-corrected performance measures.

"""

**README**

**Overview**

This Python program evaluates the performance of three machine learning models—LightGBM, XGBoost, and CatBoost—using bootstrap resampling. It calculates various performance metrics, including AUROC, accuracy, sensitivity, specificity, and more, along with their 95% confidence intervals. The program also generates histograms of the AUC distributions from the bootstrap samples, providing insight into the variability of model performance.

**Requirements**

- Python 3.x
- Required libraries: dask[dataframe], catboost, pandas, numpy, matplotlib, xgboost, lightgbm, scikit-learn

Install the necessary libraries using:

pip install dask[dataframe] catboost pandas numpy matplotlib xgboost lightgbm scikit-learn

**How to Use**

1. **Prepare Data:**
   - Place your training dataset in CSV format.
   - Ensure that the feature columns and target variable (Outcome) are correctly referenced:
     - Feature columns: "Sex":"Ferritin"
     - Target variable: "Outcome"
2. **Modify File Paths:**
   - Update the file paths in the script to point to your dataset:

train_data = pd.read_csv("path/to/your/training_data.csv", encoding="utf-8")

1. **Run the Script:**
   - Execute the script using Python:

python script_name.py

- - The program will:
    - Train the LightGBM, XGBoost, and CatBoost models on the training dataset.
    - Perform bootstrap evaluation, resampling the dataset 1,000 times to calculate performance metrics and their confidence intervals.
    - Generate and display histograms of the AUC distributions for each model.
    - Output a DataFrame containing the mean performance metrics and their 95% confidence intervals.

1. **Interpretation:**
   - **Bootstrap AUC Distribution:** Visualizes the variability in the model's AUC, helping to assess the stability of the model's performance.
   - **Performance Metrics:** Metrics such as AUROC, accuracy, sensitivity, specificity, precision, F1 score, and more are displayed along with their 95% confidence intervals, providing a comprehensive evaluation of model performance.
   - **Apparent Performance, Optimism, and Corrected Performance:** These metrics help understand the potential overfitting of the model and provide a bias-corrected estimate of performance.
2. **Output:**
   - **AUC Distribution Histograms:** Separate histograms for each model, showing the distribution of AUC scores across the bootstrap samples.
   - **Performance Metrics DataFrame:** Printed in the console, this DataFrame includes the mean values and confidence intervals for various performance metrics, formatted for easy copying to Excel or other software.
3. **Customization:**
   - The script can be modified to include additional models or change the hyperparameters for each model.
   - Adjustments can be made to the number of bootstrap samples or the metrics being evaluated.

**Example**

For demonstration purposes, the script uses generalized file paths. Replace these with the actual paths to your datasets to perform the analysis.

# Install necessary libraries if not already installed

try:

import dask.dataframe as dd

import catboost

except ModuleNotFoundError as e:

!pip install dask[dataframe]

!pip install catboost

import dask.dataframe as dd

from catboost import CatBoostClassifier

# Import other necessary libraries

import pandas as pd

import numpy as np

import matplotlib.pyplot as plt

from xgboost import XGBClassifier

import lightgbm as lgb

from catboost import CatBoostClassifier

from sklearn.metrics import (roc_auc_score, roc_curve, accuracy_score, f1_score,

precision_score, recall_score)

from sklearn.utils import resample

# Load training data

train_data = pd.read_csv("path/to/your/training_data.csv", encoding="utf-8") # Generalized file path

# Replace spaces in feature names with underscores

train_data.columns = train_data.columns.str.replace(' ', '_')

# Select explanatory variables and target variable

X_train = train_data.loc[:, "Sex":"Ferritin"]

y_train = train_data["Outcome"]

# Define models

models = {

"LightGBM": lgb.LGBMClassifier(max_depth=3, learning_rate=0.1, n_estimators=100, subsample=0.8, colsample_bytree=0.8, force_col_wise=True),

"XGBoost": XGBClassifier(eval_metric='logloss', max_depth=3, eta=0.1, min_child_weight=1, subsample=0.8, colsample_bytree=0.8),

"CatBoost": CatBoostClassifier(depth=3, learning_rate=0.1, iterations=100, auto_class_weights='Balanced', verbose=0)

}

# Define color map

colors = {

"LightGBM": "lightcoral", # Light red

"XGBoost": "lightgrey", # Light grey

"CatBoost": "lightblue" # Light blue

}

# Define dark colors for mean AUC lines

mean_line_colors = {

"LightGBM": "darkred", # Dark red

"XGBoost": "black", # Black

"CatBoost": "darkblue" # Dark blue

}

# Bootstrap evaluation function

def bootstrap_evaluation(model, X, y, n_bootstraps=1000):

rng = np.random.RandomState(seed=42)

aucs, accuracies, sensitivities, specificities, precisions, f1_scores = [], [], [], [], [], []

for i in range(n_bootstraps):

X_resampled, y_resampled = resample(X, y, replace=True, random_state=rng)

model.fit(X_resampled, y_resampled)

y_pred_proba = model.predict_proba(X)[:, 1]

y_pred = model.predict(X)

fpr, tpr, thresholds = roc_curve(y, y_pred_proba)

auc = roc_auc_score(y, y_pred_proba)

optimal_idx = np.argmax(tpr - fpr)

sensitivity = tpr[optimal_idx]

specificity = 1 - fpr[optimal_idx]

accuracy = accuracy_score(y, y_pred)

precision = precision_score(y, y_pred)

f1 = f1_score(y, y_pred)

aucs.append(auc)

accuracies.append(accuracy)

sensitivities.append(sensitivity)

specificities.append(specificity)

precisions.append(precision)

f1_scores.append(f1)

def calculate_metric_ci(metric_values, alpha=0.95):

sorted_scores = np.array(metric_values)

sorted_scores.sort()

lower = sorted_scores[int((1.0 - alpha) / 2.0 * len(sorted_scores))]

upper = sorted_scores[int((alpha + (1.0 - alpha) / 2.0) * len(sorted_scores))]

return lower, upper

metrics = {

"aucs": aucs,

"auc_mean": np.mean(aucs),

"auc_ci_lower": calculate_metric_ci(aucs)[0],

"auc_ci_upper": calculate_metric_ci(aucs)[1],

"accuracy_mean": np.mean(accuracies),

"accuracy_ci_lower": calculate_metric_ci(accuracies)[0],

"accuracy_ci_upper": calculate_metric_ci(accuracies)[1],

"sensitivity_mean": np.mean(sensitivities),

"sensitivity_ci_lower": calculate_metric_ci(sensitivities)[0],

"sensitivity_ci_upper": calculate_metric_ci(sensitivities)[1],

"specificity_mean": np.mean(specificities),

"specificity_ci_lower": calculate_metric_ci(specificities)[0],

"specificity_ci_upper": calculate_metric_ci(specificities)[1],

"precision_mean": np.mean(precisions),

"precision_ci_lower": calculate_metric_ci(precisions)[0],

"precision_ci_upper": calculate_metric_ci(precisions)[1],

"f1_mean": np.mean(f1_scores),

"f1_ci_lower": calculate_metric_ci(f1_scores)[0],

"f1_ci_upper": calculate_metric_ci(f1_scores)[1],

}

return metrics

# Create a DataFrame for statistical metrics

results = pd.DataFrame(columns=["Model", "Metric", "Mean", "95% CI Lower", "95% CI Upper"])

fig, axes = plt.subplots(1, 3, figsize=(18, 6), sharey=True)

for ax, (name, model) in zip(axes, models.items()):

# Train the model

model.fit(X_train, y_train)

# Bootstrap evaluation

metrics = bootstrap_evaluation(model, X_train, y_train)

# Calculate apparent performance, optimism, and corrected performance

apparent_performance = metrics["auc_mean"]

optimism = metrics["auc_mean"] - metrics["auc_ci_lower"]

corrected_performance = metrics["auc_mean"] - optimism

# Plot histogram

ax.hist(metrics["aucs"], bins=50, color=colors[name], alpha=0.75, label=f'{name} (Mean AUC = {metrics["auc_mean"]:.3f})')

ax.axvline(metrics["auc_mean"], color=mean_line_colors[name], linestyle='dashed', linewidth=2)

ax.set_title(f'{name} Bootstrap AUC Distribution')

ax.set_xlabel('AUC')

ax.set_ylabel('Frequency')

ax.legend(loc='lower right')

# Add statistical metrics to DataFrame

model_results = pd.DataFrame({

"Model": [name]*10, # Adjusted to 10 because we have 10 metrics including Bias-corrected performance

"Metric": ["AUC", "Accuracy", "Sensitivity", "Specificity", "Precision", "F1 Score", "Apparent Performance", "Optimism", "Corrected Performance", "Bias-corrected Performance"],

"Mean": [

metrics["auc_mean"],

metrics["accuracy_mean"],

metrics["sensitivity_mean"],

metrics["specificity_mean"],

metrics["precision_mean"],

metrics["f1_mean"],

apparent_performance,

optimism,

corrected_performance,

corrected_performance

],

"95% CI Lower": [

metrics["auc_ci_lower"],

metrics["accuracy_ci_lower"],

metrics["sensitivity_ci_lower"],

metrics["specificity_ci_lower"],

metrics["precision_ci_lower"],

metrics["f1_ci_lower"],

np.nan, # Apparent Performance does not have a CI

np.nan, # Optimism does not have a CI

np.nan, # Corrected Performance does not have a CI

np.nan # Bias-corrected Performance does not have a CI

],

"95% CI Upper": [

metrics["auc_ci_upper"],

metrics["accuracy_ci_upper"],

metrics["sensitivity_ci_upper"],

metrics["specificity_ci_upper"],

metrics["precision_ci_upper"],

metrics["f1_ci_upper"],

np.nan, # Apparent Performance does not have a CI

np.nan, # Optimism does not have a CI

np.nan, # Corrected Performance does not have a CI

np.nan # Bias-corrected Performance does not have a CI

]

})

results = pd.concat([results, model_results], ignore_index=True)

plt.tight_layout()

plt.show()

# Display statistical metrics formatted for Excel copy

results = results.sort_values(by=["Model", "Metric"])

print(results.to_string(index=False))

"""

**Title: SHAP Analysis for Machine Learning Models using D-dimer Data**

Description:

This Python script performs SHapley Additive exPlanations (SHAP) analysis for three machine learning models—LightGBM, XGBoost, and CatBoost—using D-dimer data to predict patient outcomes. The script generates customized SHAP summary, beeswarm, and dependence plots for each model, offering insights into feature importance and interaction effects. The SHAP values are visualized and saved as high-quality images, making the results suitable for inclusion as supplementary material in a major medical journal.

"""

**README**

**Overview**

This Python program performs SHapley Additive exPlanations (SHAP) analysis to interpret the predictions of three machine learning models: LightGBM, XGBoost, and CatBoost. The program generates custom SHAP summary, beeswarm, and dependence plots to visualize the impact of each feature on the model's output.

**Requirements**

- Python 3.x
- Required libraries: shap, catboost, xgboost, lightgbm, pandas, numpy, matplotlib

Install the necessary libraries using:

pip install shap catboost xgboost lightgbm pandas numpy matplotlib

**How to Use**

1. **Prepare Data:**
   - Place your training and validation datasets in CSV format.
   - Ensure that the feature columns and target variable (Outcome) are correctly referenced:
     - Feature columns: "Sex":"Ferritin"
     - Target variable: "Outcome"
2. **Modify File Paths:**
   - Update the file paths in the script to point to your datasets:

train_data = pd.read_csv("path/to/your/training_data.csv", encoding="utf-8")

test_data = pd.read_csv("path/to/your/validation_data.csv", encoding="utf-8")

1. **Run the Script:**
   - Execute the script using Python:

python script_name.py

- - The program will:
    - Train the LightGBM, XGBoost, and CatBoost models on the training dataset.
    - Perform SHAP analysis to calculate SHAP values for each model.
    - Generate and save SHAP summary, beeswarm, and dependence plots for each model.

1. **Interpretation:**
   - **Custom SHAP Summary Plot:** Shows the mean absolute SHAP value for each feature, indicating the average impact on the model's predictions.
   - **SHAP Beeswarm Summary Plot:** Visualizes the distribution of SHAP values for each feature across all instances, highlighting the interaction between features.
   - **SHAP Dependence Plot:** Displays the relationship between a specific feature's SHAP values and its actual values, providing insights into feature interactions.
2. **Output:**
   - **SHAP Summary Plots:** Separate bar plots for each model, showing the mean absolute SHAP values for all features.
   - **SHAP Beeswarm Plots:** Dot plots for each model, displaying the distribution of SHAP values for each feature.
   - **SHAP Dependence Plots:** Individual plots for each feature in each model, showing the feature's SHAP value against its actual value, with the interaction index set to "D-dimer".
3. **Customization:**
   - The script can be modified to include additional models or change the hyperparameters for each model.
   - The SHAP plots can be customized further by adjusting colors, plot sizes, or adding additional annotations.

**Example**

For demonstration purposes, the script uses generalized file paths. Replace these with the actual paths to your datasets to perform the analysis.

# Install required libraries

!pip install shap

!pip install catboost

!pip install xgboost

!pip install lightgbm

!pip install dask[dataframe]

import pandas as pd

import numpy as np

import matplotlib.pyplot as plt

import shap

from xgboost import XGBClassifier

import lightgbm as lgb

from catboost import CatBoostClassifier

# Load training and validation data

train_data = pd.read_csv("path/to/your/training_data.csv", encoding="utf-8") # Generalized file path

test_data = pd.read_csv("path/to/your/validation_data.csv", encoding="utf-8") # Generalized file path

# Replace spaces in feature names with underscores

train_data.columns = train_data.columns.str.replace(' ', '_')

test_data.columns = test_data.columns.str.replace(' ', '_')

# Select explanatory variables and target variable

X_train = train_data.loc[:, "Sex":"Ferritin"]

y_train = train_data["Outcome"]

X_test = test_data.loc[:, "Sex":"Ferritin"]

y_test = test_data["Outcome"]

# Define models

models = {

"LightGBM": lgb.LGBMClassifier(max_depth=3, learning_rate=0.1, n_estimators=100, subsample=0.8, colsample_bytree=0.8, force_col_wise=True),

"XGBoost": XGBClassifier(use_label_encoder=False, eval_metric='logloss', max_depth=3, eta=0.1, min_child_weight=1, subsample=0.8, colsample_bytree=0.8),

"CatBoost": CatBoostClassifier(depth=3, learning_rate=0.1, iterations=100, auto_class_weights='Balanced', verbose=0)

}

# Prepare for SHAP analysis

shap_values_dict = {}

explainer_dict = {}

for name, model in models.items():

# Train the model

model.fit(X_train, y_train)

# SHAP analysis

if name == "LightGBM" or name == "XGBoost":

explainer = shap.TreeExplainer(model)

shap_values = explainer.shap_values(X_train)

shap_values = shap_values[1] if isinstance(shap_values, list) else shap_values # for binary classification models

else:

explainer = shap.Explainer(model, X_train)

shap_values = explainer(X_train).values

shap_values_dict[name] = shap_values

explainer_dict[name] = explainer

# Color settings

colors = {

"LightGBM": "#ff9999", # Light red

"XGBoost": "gray", # Gray

"CatBoost": "#add8e6" # Light blue

}

# Set graph style

plt.style.use('seaborn-darkgrid')

plt.rcParams.update({

'font.size': 12,

'axes.labelsize': 14,

'axes.titlesize': 16,

'xtick.labelsize': 10,

'ytick.labelsize': 10,

'legend.fontsize': 12,

'font.family': 'serif'

})

# 1) Display custom SHAP summary plot

def custom_shap_summary_plot(model_name):

shap_values = shap_values_dict[model_name]

# Calculate the mean absolute SHAP values

mean_shap_values = np.abs(shap_values).mean(axis=0)

# Sort in descending order

sorted_idx = np.argsort(mean_shap_values)[::-1]

features = X_train.columns[sorted_idx]

values = mean_shap_values[sorted_idx]

# Plot

plt.figure(figsize=(12, 8))

plt.barh(features, values, color=colors[model_name])

plt.xlabel("mean(|SHAP value|) (average impact on model output magnitude)", fontsize=16)

# Display SHAP values on each bar

for i, v in enumerate(values):

plt.text(v + 0.0005, i, f"{v:.3f}", va='center', fontsize=10)

plt.title(f"{model_name} SHAP Summary Plot", fontsize=18)

plt.gca().invert_yaxis() # Invert Y-axis to have the largest values at the top

plt.tight_layout()

plt.savefig(f"{model_name}_shap_summary_plot.png", dpi=300)

plt.show()

# Display SHAP summary plot for each model

for model_name in models.keys():

custom_shap_summary_plot(model_name)

# 2) Display SHAP beeswarm summary plot

def shap_beeswarm_summary_plot(model_name):

shap_values = shap_values_dict[model_name]

plt.figure(figsize=(12, 8))

shap.summary_plot(shap_values, X_train, plot_type="dot", color=colors[model_name], show=False)

plt.title(f"{model_name} SHAP Beeswarm Summary Plot", fontsize=14)

plt.tight_layout()

plt.savefig(f"{model_name}_shap_beeswarm_plot.png", dpi=300)

plt.show()

# Display SHAP beeswarm summary plot for each model

for model_name in models.keys():

shap_beeswarm_summary_plot(model_name)

# 3) Output SHAP dependence plots for all features, sorted by SHAP values in descending order

def custom_shap_dependence_plot(model_name):

shap_values = shap_values_dict[model_name]

explainer = explainer_dict[model_name]

# Calculate the mean absolute SHAP values

mean_shap_values = np.abs(shap_values).mean(axis=0)

# Sort in descending order

sorted_idx = np.argsort(mean_shap_values)[::-1]

features = X_train.columns[sorted_idx]

# Output SHAP dependence plots for each feature

for i, feature in enumerate(features):

plt.figure(figsize=(8, 6))

shap.dependence_plot(feature, shap_values, X_train, interaction_index='D-dimer', show=False)

# Display SHAP value in the lower right corner with 3 decimal places

mean_shap_value = mean_shap_values[sorted_idx[i]]

plt.text(1.05, 0.05, f"SHAP value: {mean_shap_value:.3f}", transform=plt.gca().transAxes, fontsize=12, verticalalignment='bottom', horizontalalignment='right')

plt.title(f"{model_name} SHAP Dependence Plot - {feature}", fontsize=16)

plt.tight_layout()

plt.savefig(f"{model_name}_shap_dependence_plot_{feature}.png", dpi=300)

plt.show()

# Display SHAP dependence plots for each model

for model_name in models.keys():

custom_shap_dependence_plot(model_name)

"""

**Title: Machine Learning Model Evaluation for Predicting Outcomes using D-dimer Data**

Description:

This Python script evaluates the performance of multiple machine learning models—MLRA, Prediction One, LightGBM, XGBoost, and CatBoost—using D-dimer data to predict patient outcomes. The models are trained and validated on separate datasets, and performance metrics such as ROC curves and calibration plots are generated for both training and validation datasets. The script outputs high-quality visualizations, including ROC curves and calibration plots with linear regression fits, making the results suitable for inclusion as supplementary material in a major medical journal.

"""

**README**

**Overview**

**Requirements**

- Python 3.x
- Required libraries: catboost, lightgbm, xgboost, pandas, numpy, matplotlib, scikit-learn, scipy

Install the necessary libraries using:

pip install catboost lightgbm xgboost pandas numpy matplotlib scikit-learn scipy

**How to Use**

1. **Prepare Data:**
   - Place your training and validation datasets in CSV format.
   - Ensure that the feature columns and target variable (Outcome) are correctly referenced:
     - Feature columns for MLRA: ['Age', 'TP', 'D-dimer', 'AST', 'TC']
     - Target variable for MLRA and Prediction One models: Outcome
2. **Modify File Paths:**
   - Update the file paths in the script to point to your datasets:

df_train = pd.read_csv("path/to/your/training_data_mlra.csv")

df_test = pd.read_csv("path/to/your/validation_data_mlra.csv")

df_train_po = pd.read_csv("path/to/your/training_data_prediction_one.csv")

df_test_po = pd.read_csv("path/to/your/validation_data_prediction_one.csv")

train_data = pd.read_csv("path/to/your/training_data_shap.csv", encoding="utf-8")

test_data = pd.read_csv("path/to/your/validation_data_shap.csv", encoding="utf-8")

1. **Run the Script:**
   - Execute the script using Python:

python script_name.py

- - The program will:
    - Train the MLRA, Prediction One, LightGBM, XGBoost, and CatBoost models on the training datasets.
    - Evaluate each model on both the training and validation datasets using ROC and calibration curves.
    - Generate and display ROC and calibration plots, including linear regression fits to the calibration curves.

1. **Interpretation:**
   - **ROC Curve:** Provides a visual comparison of the models' discrimination ability on both the training and validation datasets, showing the area under the curve (AUC).
   - **Calibration Plot:** Assesses the agreement between predicted probabilities and observed outcomes, with regression lines provided for deeper analysis.
   - **Performance Metrics:** Metrics such as AUROC, calibration slope, and intercept are displayed for both datasets, enabling comprehensive evaluation.
2. **Output:**
   - **ROC Curve Plot:** A plot comparing the ROC curves for all models on both training and validation datasets.
   - **Calibration Plot:** A plot showing the calibration curves for all models, including linear regression fits for both datasets.
   - **High-Quality Visualizations:** Generated plots are suitable for inclusion in supplementary materials for a major medical journal.
3. **Customization:**
   - The script can be modified to include additional models or change hyperparameters for each model.
   - Adjustments to the number of bins in the calibration plot or the model parameters can be made for further tuning.

**Example**

For demonstration purposes, the script uses generalized file paths. Replace these with the actual paths to your datasets to perform the analysis.

# Install required libraries

!pip install catboost

import pandas as pd

import numpy as np

import matplotlib.pyplot as plt

from sklearn.metrics import roc_curve, auc

from sklearn.calibration import calibration_curve

from scipy.stats import linregress

from lightgbm import LGBMClassifier

from xgboost import XGBClassifier

from catboost import CatBoostClassifier

# Program 1: MLRA Data Preparation and Creation of ROC Curves and Calibration Plots

df_train = pd.read_csv("path/to/your/training_data_mlra.csv") # Generalized file path

df_test = pd.read_csv("path/to/your/validation_data_mlra.csv") # Generalized file path

df_train = df_train.dropna()

df_test = df_test.dropna()

X_train = df_train[['Age', 'TP', 'D-dimer', 'AST', 'TC']]

X_test = df_test[['Age', 'TP', 'D-dimer', 'AST', 'TC']]

y_train = df_train['Outcome']

y_test = df_test['Outcome']

X_train['Log_D-dimer'] = np.log(X_train['D-dimer'])

X_train['Log_AST'] = np.log(X_train['AST'])

X_train['Log_TC'] = np.log(X_train['TC'])

X_test['Log_D-dimer'] = np.log(X_test['D-dimer'])

X_test['Log_AST'] = np.log(X_test['AST'])

X_test['Log_TC'] = np.log(X_test['TC'])

X_train = X_train[['Age', 'TP', 'Log_D-dimer', 'Log_AST', 'Log_TC']]

X_test = X_test[['Age', 'TP', 'Log_D-dimer', 'Log_AST', 'Log_TC']]

def predict_proba(X):

beta = np.array([1.370, 0.025, -0.367, 0.433, 0.450, -1.373])

z = np.dot(np.c_[np.ones((X.shape[0], 1)), X], beta)

p = 1 / (1 + np.exp(-z))

return p

y_train_pred_proba = predict_proba(X_train)

y_test_pred_proba = predict_proba(X_test)

fpr_train_mlra, tpr_train_mlra, _ = roc_curve(y_train, y_train_pred_proba)

auc_train_mlra = auc(fpr_train_mlra, tpr_train_mlra)

fpr_test_mlra, tpr_test_mlra, _ = roc_curve(y_test, y_test_pred_proba)

auc_test_mlra = auc(fpr_test_mlra, tpr_test_mlra)

prob_true_train_mlra, prob_pred_train_mlra = calibration_curve(y_train, y_train_pred_proba, n_bins=10)

prob_true_test_mlra, prob_pred_test_mlra = calibration_curve(y_test, y_test_pred_proba, n_bins=10)

# Program 2: Prediction One Data Preparation and Creation of ROC Curves and Calibration Plots

df_train_po = pd.read_csv("path/to/your/training_data_prediction_one.csv") # Generalized file path

df_test_po = pd.read_csv("path/to/your/validation_data_prediction_one.csv") # Generalized file path

y_train_po = df_train_po.iloc[:, 5]

y_train_pred_proba_po = df_train_po.iloc[:, 1]

y_test_po = df_test_po.iloc[:, 13]

y_test_pred_proba_po = df_test_po.iloc[:, 3]

fpr_train_po, tpr_train_po, _ = roc_curve(y_train_po, y_train_pred_proba_po)

auc_train_po = auc(fpr_train_po, tpr_train_po)

fpr_test_po, tpr_test_po, _ = roc_curve(y_test_po, y_test_pred_proba_po)

auc_test_po = auc(fpr_test_po, tpr_test_po)

prob_true_train_po, prob_pred_train_po = calibration_curve(y_train_po, y_train_pred_proba_po, n_bins=10)

prob_true_test_po, prob_pred_test_po = calibration_curve(y_test_po, y_test_pred_proba_po, n_bins=10)

# Program 3: LightGBM, XGBoost, CatBoost Data Preparation and Creation of ROC Curves and Calibration Plots

train_data = pd.read_csv("path/to/your/training_data_shap.csv", encoding="utf-8") # Generalized file path

test_data = pd.read_csv("path/to/your/validation_data_shap.csv", encoding="utf-8") # Generalized file path

X_train_shap = train_data.loc[:, "Sex":"Ferritin"]

y_train_shap = train_data["Outcome"]

X_test_shap = test_data.loc[:, "Sex":"Ferritin"]

y_test_shap = test_data["Outcome"]

# LightGBM

model_lgb = LGBMClassifier(max_depth=3, learning_rate=0.1, n_estimators=100, subsample=0.8, colsample_bytree=0.8, force_col_wise=True)

model_lgb.fit(X_train_shap, y_train_shap)

y_train_pred_proba_lgb = model_lgb.predict_proba(X_train_shap)[:, 1]

y_test_pred_proba_lgb = model_lgb.predict_proba(X_test_shap)[:, 1]

fpr_train_LightGBM, tpr_train_LightGBM, _ = roc_curve(y_train_shap, y_train_pred_proba_lgb)

auc_train_LightGBM = auc(fpr_train_LightGBM, tpr_train_LightGBM)

fpr_test_LightGBM, tpr_test_LightGBM, _ = roc_curve(y_test_shap, y_test_pred_proba_lgb)

auc_test_LightGBM = auc(fpr_test_LightGBM, tpr_test_LightGBM)

prob_true_train_LightGBM, prob_pred_train_LightGBM = calibration_curve(y_train_shap, y_train_pred_proba_lgb, n_bins=10)

prob_true_test_LightGBM, prob_pred_test_LightGBM = calibration_curve(y_test_shap, y_test_pred_proba_lgb, n_bins=10)

# XGBoost

model_xgb = XGBClassifier(use_label_encoder=False, eval_metric='logloss', max_depth=3, eta=0.1, min_child_weight=1, subsample=0.8, colsample_bytree=0.8)

model_xgb.fit(X_train_shap, y_train_shap)

y_train_pred_proba_xgb = model_xgb.predict_proba(X_train_shap)[:, 1]

y_test_pred_proba_xgb = model_xgb.predict_proba(X_test_shap)[:, 1]

fpr_train_XGBoost, tpr_train_XGBoost, _ = roc_curve(y_train_shap, y_train_pred_proba_xgb)

auc_train_XGBoost = auc(fpr_train_XGBoost, tpr_train_XGBoost)

fpr_test_XGBoost, tpr_test_XGBoost, _ = roc_curve(y_test_shap, y_test_pred_proba_xgb)

auc_test_XGBoost = auc(fpr_test_XGBoost, tpr_test_XGBoost)

prob_true_train_XGBoost, prob_pred_train_XGBoost = calibration_curve(y_train_shap, y_train_pred_proba_xgb, n_bins=10)

prob_true_test_XGBoost, prob_pred_test_XGBoost = calibration_curve(y_test_shap, y_test_pred_proba_xgb, n_bins=10)

# CatBoost

model_cat = CatBoostClassifier(depth=3, learning_rate=0.1, iterations=100, auto_class_weights='Balanced', verbose=0)

model_cat.fit(X_train_shap, y_train_shap)

y_train_pred_proba_cat = model_cat.predict_proba(X_train_shap)[:, 1]

y_test_pred_proba_cat = model_cat.predict_proba(X_test_shap)[:, 1]

fpr_train_CatBoost, tpr_train_CatBoost, _ = roc_curve(y_train_shap, y_train_pred_proba_cat)

auc_train_CatBoost = auc(fpr_train_CatBoost, tpr_train_CatBoost)

fpr_test_CatBoost, tpr_test_CatBoost, _ = roc_curve(y_test_shap, y_test_pred_proba_cat)

auc_test_CatBoost = auc(fpr_test_CatBoost, tpr_test_CatBoost)

prob_true_train_CatBoost, prob_pred_train_CatBoost = calibration_curve(y_train_shap, y_train_pred_proba_cat, n_bins=10)

prob_true_test_CatBoost, prob_pred_test_CatBoost = calibration_curve(y_test_shap, y_test_pred_proba_cat, n_bins=10)

# Model settings for color and line style

models = ['MLRA_Training', 'MLRA_Validation', 'PredictionOne_Training', 'PredictionOne_Validation',

'LightGBM_Training', 'LightGBM_Validation', 'XGBoost_Training', 'XGBoost_Validation',

'CatBoost_Training', 'CatBoost_Validation']

colors = ['#1f77b4', '#1f77b4', '#ff7f0e', '#ff7f0e', '#2ca02c', '#2ca02c', '#d62728', '#d62728', '#9467bd', '#9467bd']

linestyles = ['-', '--', '-', '--', '-', '--', '-', '--', '-', '--']

linewidths = [2.5, 1.8, 2.5, 1.8, 2.8, 1.8, 2.8, 1.8, 2.8, 1.8]

# ROC Curve Plot

plt.figure(figsize=(12, 10))

for model, color, linestyle, linewidth in zip(models, colors, linestyles, linewidths):

if 'MLRA' in model:

if 'Training' in model:

fpr, tpr, auc_value = fpr_train_mlra, tpr_train_mlra, auc_train_mlra

else:

fpr, tpr, auc_value = fpr_test_mlra, tpr_test_mlra, auc_test_mlra

elif 'PredictionOne' in model:

if 'Training' in model:

fpr, tpr, auc_value = fpr_train_po, tpr_train_po, auc_train_po

else:

fpr, tpr, auc_value = fpr_test_po, tpr_test_po, auc_test_po

elif 'LightGBM' in model:

if 'Training' in model:

fpr, tpr, auc_value = fpr_train_LightGBM, tpr_train_LightGBM, auc_train_LightGBM

else:

fpr, tpr, auc_value = fpr_test_LightGBM, tpr_test_LightGBM, auc_test_LightGBM

elif 'XGBoost' in model:

if 'Training' in model:

fpr, tpr, auc_value = fpr_train_XGBoost, tpr_train_XGBoost, auc_train_XGBoost

else:

fpr, tpr, auc_value = fpr_test_XGBoost, tpr_test_XGBoost, auc_test_XGBoost

elif 'CatBoost' in model:

if 'Training' in model:

fpr, tpr, auc_value = fpr_train_CatBoost, tpr_train_CatBoost, auc_train_CatBoost

else:

fpr, tpr, auc_value = fpr_test_CatBoost, tpr_test_CatBoost, auc_test_CatBoost

plt.plot(fpr, tpr, linestyle=linestyle, linewidth=linewidth, color=color, label=f'{model} (AUC = {auc_value:.3f})')

plt.plot([0, 1], [0, 1], 'k:', label='No Skill Line')

plt.xlim([0.0, 1.0])

plt.ylim([0.0, 1.05])

plt.xlabel('1 - Specificity')

plt.ylabel('Sensitivity')

plt.title('ROC Curves Comparison')

plt.legend(loc='lower right')

plt.grid(alpha=0.3)

plt.show()

# Calibration Plot

plt.figure(figsize=(12, 10))

for model, color, linestyle, linewidth in zip(models, colors, linestyles, linewidths):

if 'MLRA' in model:

if 'Training' in model:

prob_true, prob_pred = prob_true_train_mlra, prob_pred_train_mlra

else:

prob_true, prob_pred = prob_true_test_mlra, prob_pred_test_mlra

elif 'PredictionOne' in model:

if 'Training' in model:

prob_true, prob_pred = prob_true_train_po, prob_pred_train_po

else:

prob_true, prob_pred = prob_true_test_po, prob_pred_test_po

elif 'LightGBM' in model:

if 'Training' in model:

prob_true, prob_pred = prob_true_train_LightGBM, prob_pred_train_LightGBM

else:

prob_true, prob_pred = prob_true_test_LightGBM, prob_pred_test_LightGBM

elif 'XGBoost' in model:

if 'Training' in model:

prob_true, prob_pred = prob_true_train_XGBoost, prob_pred_train_XGBoost

else:

prob_true, prob_pred = prob_true_test_XGBoost, prob_pred_test_XGBoost

elif 'CatBoost' in model:

if 'Training' in model:

prob_true, prob_pred = prob_true_train_CatBoost, prob_pred_train_CatBoost

else:

prob_true, prob_pred = prob_true_test_CatBoost, prob_pred_test_CatBoost

slope, intercept, _, _, _ = linregress(prob_pred, prob_true)

marker = 'o' if 'Training' in model else '^'

plt.plot(prob_pred, prob_true, marker, color=color, label=f'{model} Calibration')

plt.plot(prob_pred, intercept + slope * np.array(prob_pred), linestyle=linestyle, color=color, linewidth=linewidth,

label=f'{model} Fit (Slope={slope:.3f}, Intercept={intercept:.3f})')

plt.plot([0, 1], [0, 1], 'k--', label='Perfect Calibration')

plt.xlabel('Mean Predicted Probability')

plt.ylabel('Fraction of Positives')

plt.title('Calibration Plots Comparison')

# Arrange legend in 3 columns

plt.legend(loc='upper center', bbox_to_anchor=(0.5, -0.1), ncol=3)

plt.grid(alpha=0.3)

plt.show()

**R program codes for interaction analysis using Firth’s penalized logistic regression in the study**

## Background

To address the potential modifying effect of underlying malignancy on the association between D-dimer levels and 72-hour fatality, we performed an exploratory interaction analysis using Firth’s penalized logistic regression. This approach is suitable for handling rare events or data separation issues. Analysis was performed using R on Google Colaboratory.

## Google Colab R Code Use

# Install and load required packages
install.packages('logistf')
library(logistf)

# Load data
data <- read.csv('/content/Interaction Analysis.csv')

# Create interaction term
data$Interaction_term <- data$log_D_dimer * data$Malignancy_flag

# Run Firth's logistic regression
model <- logistf(formula = Outcome ~ log_D_dimer + Malignancy_flag + Interaction_term, data = data)
summary(model)

## Output Summary

The output from the Firth’s penalized logistic regression model is as follows:

Coefficients:
 - Intercept: Coef = -4.485, OR = 0.0013, 95% CI [0.0081, 0.0155], *p* < 0.001
 - log_D_dimer: Coef = 1.320, OR = 3.745, 95% CI [2.8902, 4.885], *p* < 0.001
 - Malignancy_flag: Coef = 9.721, OR = 16663.950, 95% CI [1.3613, 144619211867.514], *p* = 0.046
 - Interaction_term: Coef = -2.566, OR = 0.0769, 95% CI [0.0000, 242202.638], *p* = 0.398

Model Statistics:
 - Likelihood ratio test = 345.71, *p* < 0.001
 - Wald test = 1805.05, *p* < 0.001
 - Number of observations = 5,158
