# Supplementary material for: Routine Laboratory Tests Predict 72‐h Fatality in Patients With D‐Dimer Levels ≥ 2 μg/mL: A Retrospective Cohort Study Comparing Statistical and Machine Learning Models
Source: J Clin Lab Anal. 2025 Sep 3;39(18):e70091. doi: 10.1002/jcla.70091 (PMC12459218; doi:10.1002/jcla.70091)
Supplement: Supplementary file 2 — DATA S2: jcla70091‐sup‐0002‐supinfo02.docx. Supplementary Text 1: Sample Size Estimation. Supplementary Text 2: Routine Laboratory Tests. Supplementary Text 3: List of Variables. Supplementary Text 4: Box‐Cox Transformation Formulae. Supplementary Text 5: Model 1 Development Using Multivariate Logistic Regression Analysis (MLRA). Supplementary Text 6: Model 2 Development Using Prediction One. Supplementary Text 7: Hyperparameters for Gradient Boosting Decision Trees (GBDTs). Supplementary Text 8: Bootstrap Internal Validation. Supplementary Text 9: External Validation of Models 1 to 5. Supplementary Text 10: Statistical Software and Programming Details. [file JCLA-39-e70091-s003.docx]

**Supplementary texts**

1. **Supplementary text 1: Sample Size Estimation**

Sample size estimation was performed using G*Power version 3.1.9.4 [1]. The initial calculation determined a required sample size of 652, based on the following parameters:

- **Test family**: z tests
- **Statistical test**: Logistic regression
- **Type of power analysis**: A priori (compute required sample size given α, power, and effect size)
- **Pr(Y=1 | X=1) under H1**: 0.05 (probability of death for patients with D-dimer levels ≥ 2 μg/mL)
- **Pr(Y=1 | X=1) under H0**: 0.01 (probability of death for patients with D-dimer levels < 2 μg/mL)
- **α error probability**: 0.05 (Type I error)
- **Power (1-β error probability)**: 0.8 (80% power)
- **R-squared for other covariates**: 0.09 (moderate variance explained by covariates)
- **X distribution**: Binomial
- **X parameter (π)**: 0.4 (proportion of cases with D-dimer levels ≥ 2 μg/mL)

To enhance statistical power, the dataset was expanded to 5,158 cases.

1. **Supplementary text 2: Routine Laboratory Tests**

This section outlines the instruments and methods used for routine hematological, biochemical, and coagulation tests at the Department of Laboratory Medicine. The following automated systems were employed:

- **Hematology**: Complete blood counts (CBC) were performed using the XN-3100™ system (Sysmex Corporation, Kobe, Japan).
- **Biochemistry**: Routine biochemical tests were measured using the BioMajesty™ JCA-BM6010/C system (JEOL Ltd, Akishima, Japan).
- **Coagulation**: Coagulation tests, including D-dimer, were conducted using the CP3000™ system (SEKISUI MEDICAL CO., LTD., Tokyo, Japan), with D-dimer levels measured using the Nanopia® D-dimer reagent, a latex agglutination immunoassay with a monoclonal antibody specific for DD fragments.

1. **Supplementary text 3: List of Variables**

A comprehensive list of the 40 routine hematological, biochemical, and coagulation tests is provided. These variables were used to analyze factors associated with 72-hour fatality. The full set of variables included:

- **Demographics**: Age, sex
- **Hematology**: White blood cells (WBC), red blood cells (RBC), hemoglobin (Hb), hematocrit (Hct), platelets (Plt)
- **Biochemistry**: Total protein (TP), albumin (ALB), total bilirubin (T-Bili), direct bilirubin (D-Bili), aspartate aminotransferase (AST), alanine aminotransferase (ALT), γ-glutamyl transferase, lactate dehydrogenase (LD), alkaline phosphatase (ALP), creatine kinase (CK), amylase, C-reactive protein (CRP), sodium (Na), potassium (K), chloride (Cl), calcium (Ca), serum inorganic phosphorus (IP), magnesium (Mg), blood urea nitrogen (BUN), creatinine (Cr), uric acid (UA), total cholesterol (TC), triglycerides (TG), high-density lipoprotein cholesterol (HDL-C), low-density lipoprotein cholesterol (LDL-C), random plasma glucose (RPG), HbA1c, serum iron, Ferritin
- **Coagulation**: Prothrombin time–international normalized ratio (PT-INR), fibrinogen, activated partial thromboplastin time (APTT), antithrombin III, fibrin/fibrinogen degradation products (FDP), D-dimer

1. **Supplementary Text 4: Box-Cox Transformation Formulae**

Details of the Box-Cox transformation applied to non-normally distributed continuous laboratory test variables are provided. The λ values for each variable and the rationale for their transformation are included.

All continuous laboratory test results were assessed for normality. Variables that deviated from normal distribution were transformed using the Box-Cox formula [2]:

$$X=\frac{\chi^{\lambda}-1}{\lambda}\ldots\left( \lambda\neq0.0 \right); X=\log\left( \chi\right)\ldots(\lambda=0.0)$$

where x and X represent the values before and after transformation, respectively, and λ is the transformation parameter.

**Steps:**

Univariate logistic regression analysis was initially performed to identify potential factors associated with 72-hour fatality. Crude odds ratios (ORs) and 95% confidence intervals (CIs) were calculated for each factor.

Covariates with significant ORs were further evaluated using receiver-operating characteristic (ROC) analysis, and the area under the curve (AUC) was calculated to measure discriminatory power.

Multivariate logistic regression analysis (MLRA) was then conducted for significant variables from the univariate analysis, adjusting for age and sex. Adjusted ORs with 95% CIs were reported.

1. **Supplementary Text 5: Model 1 Development Using Multivariate Logistic Regression Analysis (MLRA)**

This section outlines the full process for developing Model 1, including variable selection methods, the formula for calculating the probability of 72-hour fatality, and criteria for final model selection based on AUC, AIC, and multicollinearity (VIF ≥ 5).

The development followed the Transparent Reporting of a Multivariate Predictive Model for Individual Prognosis or Diagnosis plus Artificial Intelligence (TRIPOD+AI) statement [3].

**MLRA Process**:

- MLRA was employed to estimate the effect of multiple independent variables on the binary outcome of 72-hour fatality. The probability *p* of a patient being in the fatal group was modeled as follows:

$$\rho=\frac{1}{1+e^{-\chi}}; X=\beta_{0}+\sum_{i=0}^{np} \beta_{i}\chi_{i}$$

Where *X* represents a linear combination of selected variables (χi for i = 1,…, np), and *βi* is the regression coefficient for each variable. The outcome variable was defined as 72-hour fatality (yes = 1, no = 0) following the last D-dimer test. Explanatory variables included clinical laboratory parameters and age.

**Variable Selection**:

- A variable reduction method was applied, beginning with a full model. The selection process aimed for a balance between retaining valid data through listwise elimination and ensuring at least 10 events (deaths) per variable. Model performance was optimized for the highest possible AUC and lowest Akaike Information Criterion (AIC) [4].
- Multicollinearity was assessed using the variance inflation factor (VIF), with variables showing VIF ≥ 5 excluded to avoid redundancy.

**Final Model Evaluation**:

- MLRA and ROC analyses were performed to estimate the intercept, regression coefficients, χ² value, p-value, VIF, adjusted OR, 95% CIs, AUC, AIC, sensitivity, and specificity.

1. **Supplementary Text 6: Model 2 Development Using Prediction One™**

This section describes the development of Model 2 for predicting 72-hour fatality using **Prediction One™** software. The process involved automatic model generation from raw clinical data, utilizing 2-fold internal cross-validation and permutation feature importance for variable evaluation.

**Model Building Process**: Model 2 was established for patients with D-dimer levels ≥2 μg/mL using **Prediction One™** (Sony Network Communications Inc., Tokyo, Japan, <https://predictionone.sony.biz/>). The raw data from Dataset-A was input without any manual preprocessing or normality checks, as the software automatically handles these steps.

**Permutation Feature Importance**: To assess the contribution of each variable to the 72-hour fatality prediction, **Prediction One™** uses a permutation feature importance method. This approach identifies key variables by measuring the impact on the model's output when a variable is shuffled. However, it does not provide SHapley Additive Explanation (SHAP) values, which are used in other machine learning models for detailed interpretability.

**Performance Metrics**: The software provides key performance indicators, including the **area under the curve (AUC)**, **F-score**, and **recall**, which are calculated automatically during the model-building process.

1. **Supplementary Text 7: Hyperparameters for Gradient Boosting Decision Trees (GBDTs)**

This section outlines the development and optimization of the machine learning models using gradient boosting decision trees (GBDTs): **LightGBM (Model 3)**, **XGBoost (Model 4)**, and **CatBoost (Model 5)**. These models were selected for their efficiency in handling large, complex medical datasets and their ability to model non-linear relationships. For all models, raw data from **Dataset-A** was input without normality checks to account for potential non-normal distributions in the clinical variables.

The models were evaluated using SHAP (SHapley Additive exPlanations) values to interpret the importance of each feature and to address the "black-box" issue inherent in machine learning. SHAP value bar plots ranked features by their contribution to the prediction, while beeswarm summary plots visualized the overall feature impact. SHAP dependence plots, with the interaction index set to D-dimer, were used to analyze the relationship between specific features and the predicted outcomes.

**Hyperparameter Settings**: To optimize the performance of the models, the following hyperparameter settings were applied:

- **LightGBM (Model 3)** used a maximum depth of 3 to prevent overfitting, a learning rate of 0.1 to control the step size during gradient descent, 100 estimators, a subsample rate of 0.8 to reduce overfitting by training on 80% of the data, and a column sampling rate (colsample_bytree) of 0.8. Additionally, the model was configured to enforce column-wise processing (force_col_wise=True) for efficiency with sparse data.
- **XGBoost (Model 4)** applied a log loss evaluation metric (eval_metric='logloss') to assess model performance, a maximum depth of 3, an eta (learning rate) of 0.1, and a minimum child weight of 1 to control the model's complexity. The model used an 80% subsampling rate and an 80% column sampling rate for training (subsample=0.8, colsample_bytree=0.8).
- **CatBoost (Model 5)** was trained with a depth of 3, a learning rate of 0.1, and 100 iterations. The class imbalance was addressed using automatically balanced class weights (auto_class_weights='Balanced'), and verbose logging was disabled (verbose=0).

These hyperparameters were selected to ensure optimal model performance, balancing predictive accuracy, model interpretability, and computational efficiency.

1. **Supplementary Text 8: Bootstrap Internal Validation**

This section provides details on the internal validation process for **Model 1** and **Models 3-5** using 1,000 bootstrap resamples from **Dataset-A**. The bootstrap method was used to assess the stability and predictive accuracy of the models.

For each of the 1,000 resamples drawn with replacement, **ROC curves** were generated, and the **AUC** values were calculated. From these resamples, the **mean** and **95% confidence intervals (CIs)** for the following performance metrics were computed: **AUC**, **sensitivity**, **specificity**, **accuracy**, **precision**, and **F1 score**.

To further evaluate model reliability, **apparent performance**, **optimism**, and **bias-corrected performance** were also estimated. These estimates help assess how well the models generalize to unseen data, providing a measure of the model's robustness and predictive accuracy.

1. **Supplementary Text 9: External Validation of Models 1 to 5**

The external validation of **Models 1 to 5** was performed by applying each model to **Dataset-B**. The performance metrics compared included **AUC**, **95% CI of AUC**, **sensitivity**, **specificity**, **accuracy**, **precision**, **recall**, **F1 score**, **log loss**, **Matthews Correlation Coefficient (MCC)**, and **Cohen’s kappa**. Higher values in these metrics indicate better model performance, while a lower **log-loss** value signifies better predictive accuracy.

The sensitivity and specificity for each model were calculated at the optimal threshold determined by the highest **Youden's index**. Additionally, the results from **Dataset-B** were compared with those from **Dataset-A** to evaluate any potential **overfitting** across the models.

1. **Supplementary Text 10: Statistical Software and Programming Details**

This section provides descriptions of the software used for the analysis. **StatFlex** was utilized for the development and analysis of **Model 1**, while **Prediction One™** (Sony Network Communications Inc., Tokyo, Japan; <https://predictionone.sony.biz/>) was used for **Model 2**. For **Models 3-5**, Python libraries including **LightGBM**, **XGBoost**, and **CatBoost** were employed.

The Python code used for model development, bootstrap resampling, and generating calibration plots is also provided for “Supplementary file of program codes”, ensuring reproducibility and transparency in the analysis process.

References

1. Faul F, Erdfelder E, Buchner A, Lang AG. Statistical power analyses using G*Power 3.1: tests for correlation and regression analyses. Behav Res Methods 2009;41:1149-60.

2. Ichihara K, Boyd JC, Intervals ICoR, Decision L. An appraisal of statistical procedures used in derivation of reference intervals. Clin Chem Lab Med 2010;48:1537-51.

3. Collins GS, Moons KGM, Dhiman P, Riley RD, Beam AL, Van Calster B, et al. TRIPOD+AI statement: updated guidance for reporting clinical prediction models that use regression or machine learning methods. BMJ 2024;385:e078378.

4. Akaike H. A new look at the statistical model identification. IEEE Transactions on Automatic Control 1974;19:716-23.
