# Supplementary material for: Routine Laboratory Tests Predict 72‐h Fatality in Patients With D‐Dimer Levels ≥ 2 μg/mL: A Retrospective Cohort Study Comparing Statistical and Machine Learning Models
Source: J Clin Lab Anal. 2025 Sep 3;39(18):e70091. doi: 10.1002/jcla.70091 (PMC12459218; doi:10.1002/jcla.70091)
Supplement: Supplementary file 3 — DATA S3: jcla70091‐sup‐0003‐Figures.docx. FIGURE S1: Patient selection flow diagram. FIGURES S2–S8: SHAP dependence plots. FIGURE S3: SHAP dependence plots for phosphorus, age, and D‐dimer Phosphorus. FIGURE S4: SHAP dependence plots for calcium, AST, and RPG calcium. FIGURE S5: SHAP dependence plots for ALP, BUN, and HbA1c alkaline phosphatase (ALP). FIGURE S6: SHAP dependence plots for CRP, total protein, and potassium. FIGURE S7: SHAP dependence plots for platelet counts, TG, and PT‐INR platelet counts (Plt). FIGURE S8: SHAP dependence plots for APTT, creatinine, and γ‐GTP activated partial thromboplastin time (APTT). [file JCLA-39-e70091-s004.docx]

**Supplementary Figures**

**
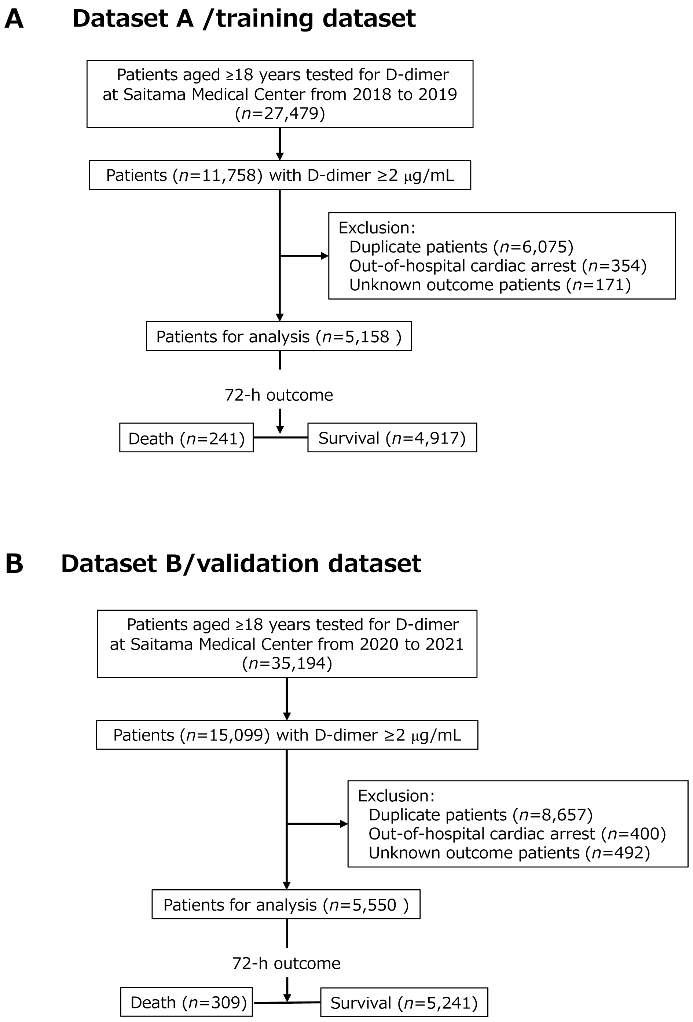
**

**Supplementary Figure 1. Patient selection flow diagram**

The patient selection process from initial cohort identification to the final datasets used for model development (A) and validation (B).

**Supplementary Figures 2-8. SHAP dependence plots**

The figure presents SHAP dependence plots for the top 21 features in LightGBM (column A), arranged in descending order of the SHAP values. For each feature, the corresponding SHAP dependence plots from XGBoost (column B) and CatBoost (column C) are displayed to the right, allowing direct comparison across the models. Each plot illustrates the relationship between the feature value (on the horizontal axis) and its SHAP value (on the vertical axis), with the interaction index set to D-dimer. The SHAP values for each feature are labeled in the bottom-right corner of each plot as "SHAP value." Red dots represent fatal cases, while blue dots represent survival cases, providing a clear visual distinction between outcomes. This layout facilitates an insightful comparison of feature impact on model predictions across the three machine learning models.

LightGBM, Light Gradient Boosting Machine, XGBoost, Extreme Gradient Boosting; CatBoost, Categorical Boosting; SHAP, SHapley Additive exPlanations

**
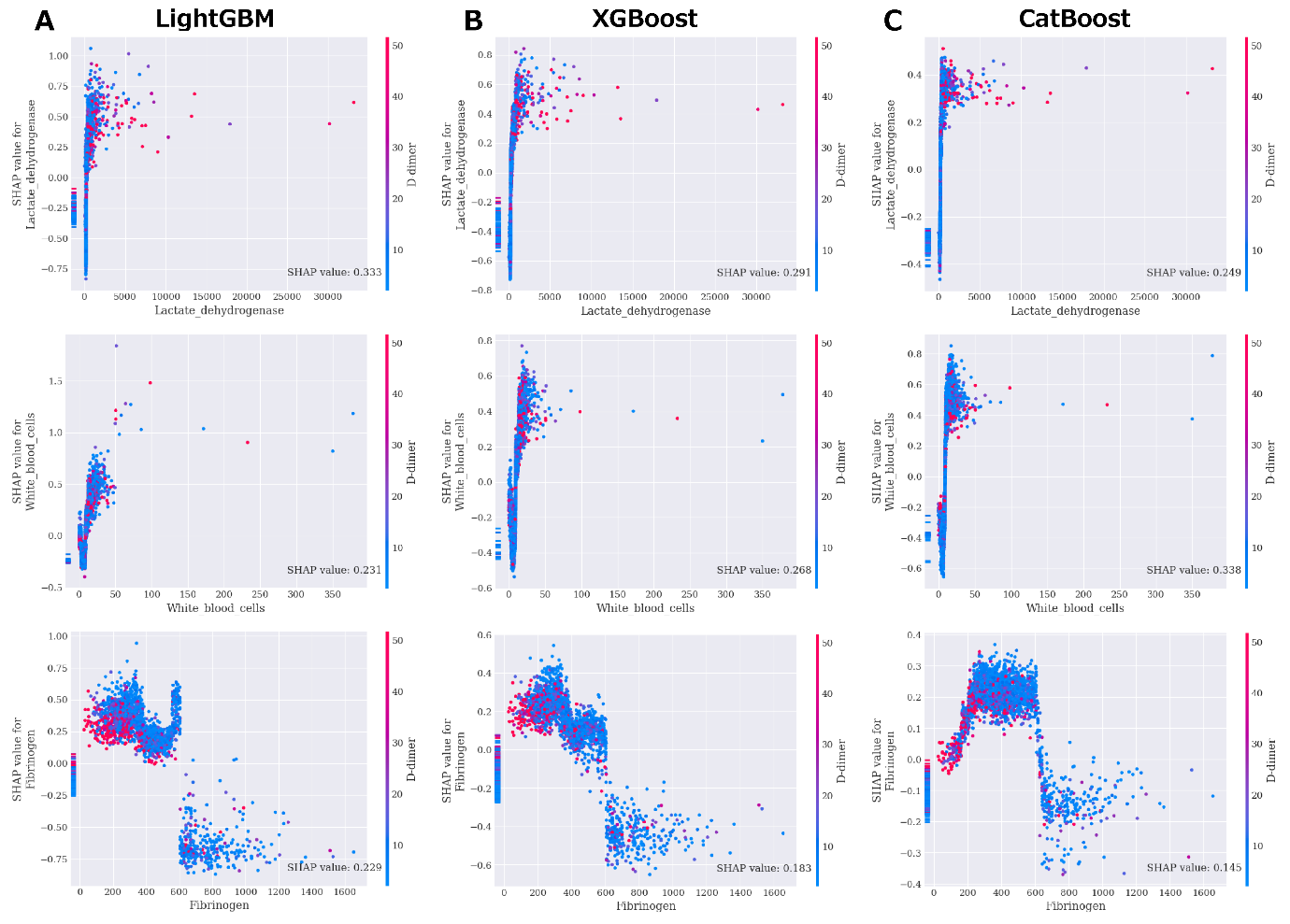
Supplementary Figure 2. SHAP Dependence Plots for LD, WBC, and Fibrinogen**

**Lactate dehydrogenase (LD)**: In all three models, higher LD levels exhibit a square-root logarithmic curve with the SHAP values, where the mid-range LD values are associated with higher SHAP values.

**White blood cells (WBC)**: In the XGBoost and CatBoost models, WBC levels resemble a square-root logarithmic relationship with the SHAP values, with the mid-range WBC count associated with higher SHAP values, indicating increased risk. In contrast, the LightGBM model displays a linear increase in the SHAP values as the WBC count rises.

**Fibrinogen**: In LightGBM and XGBoost, fibrinogen levels exhibit a reverse sigmoidal relationship with the SHAP values, where higher fibrinogen levels are associated with gradually decreasing SHAP values, indicating a negative contribution to risk predictions at elevated levels. In contrast, CatBoost displays an inverted U-shaped relationship, with the SHAP values peaking at moderate fibrinogen levels, suggesting a higher predicted risk at these levels and a reduced impact at both the lower and higher extremes.

**
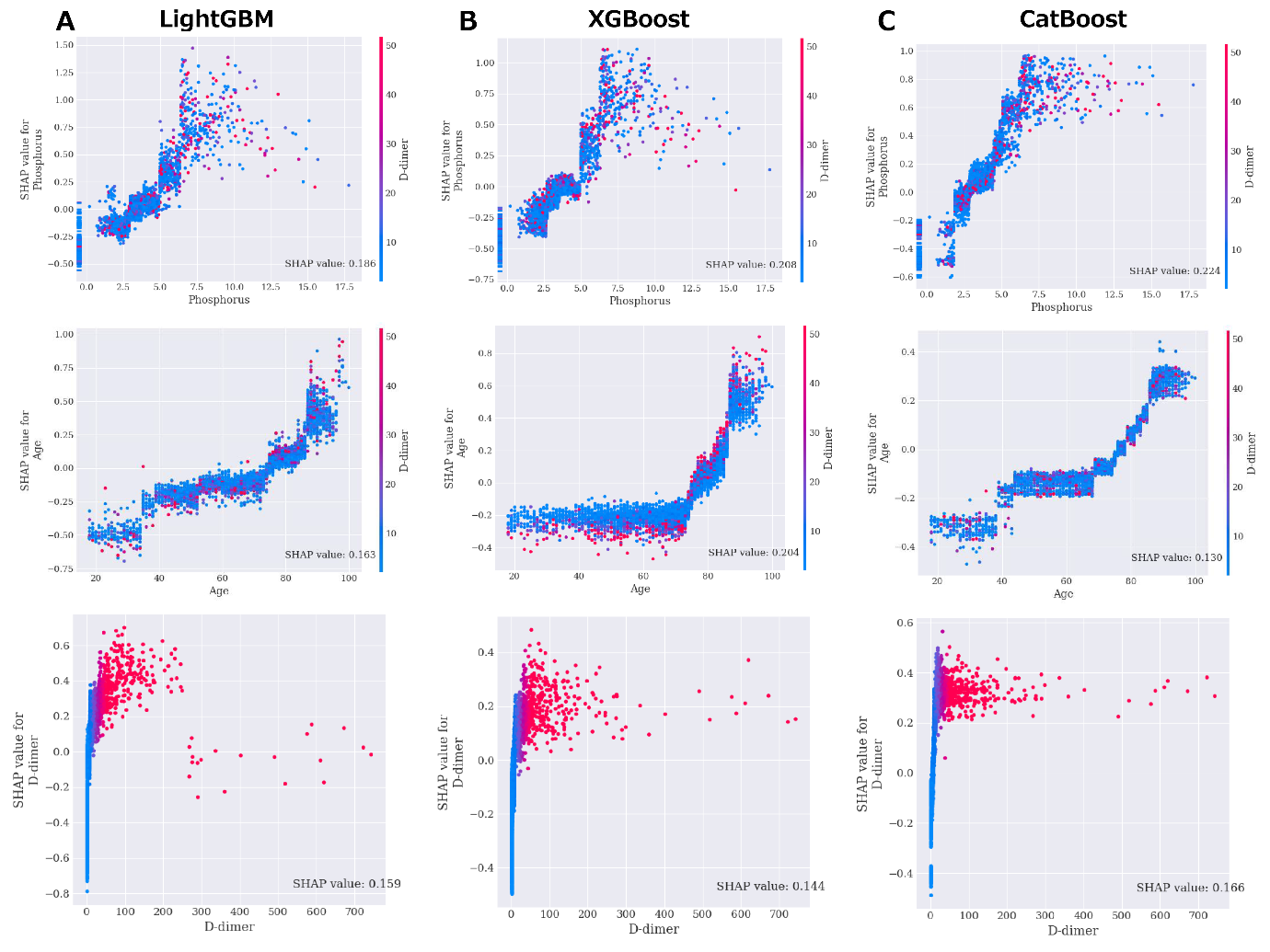
Supplementary Figure 3. SHAP Dependence Plots for Phosphorus, Age, and D-dimer**

**Phosphorus**: LightGBM and XGBoost display an inverted U-shaped pattern, with the SHAP values peaking at moderate phosphorus concentrations and decreasing at both the low and high extremes, indicating a more complex, nonlinear relationship with risk. In the CatBoost model, phosphorus concentration exhibits an upward-sloping inverted L-shaped relationship with the SHAP values, where the SHAP values increase as phosphorus levels rise from low to moderate concentrations and then plateau at high levels.

**Age**: Older age was associated with higher SHAP values, reflecting a positive impact on predicted risk. This relationship was relatively linear in LightGBM and CatBoost, while in XGBoost, the SHAP values increase linearly after approximately 60 years of age.

**D-dimer**: In all three models, D-dimer levels show nonlinear response with the SHAP values increasing significantly only at higher D-dimer concentrations.

**
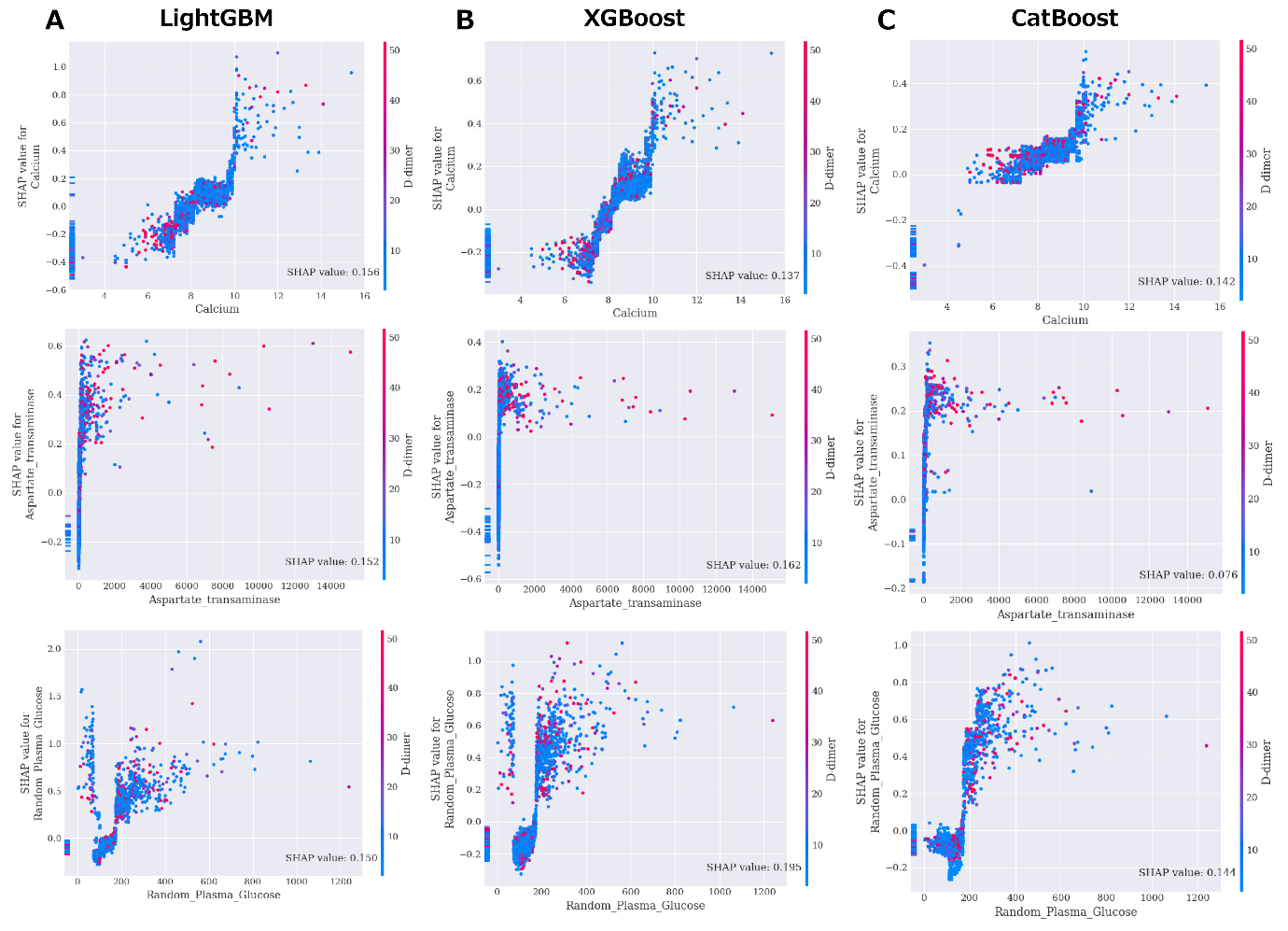
**

**Supplementary Figure 4. SHAP Dependence Plots for Calcium, AST, and RPG**

**Calcium**: Increased calcium levels correspond to increased SHAP values in all models. The linear relationship is most pronounced for LightGBM and XGBoost, whereas CatBoost exhibits a slightly nonlinear pattern.

**Aspartate transaminase (AST)**: AST levels exhibit a convex upward curve with the SHAP values across all models, where the mid-low range AST values are associated with higher SHAP values.

**Random plasma glucose (RPG)**: RPG levels exhibit a nonlinear relationship with the SHAP values across all models. In LightGBM and XGBoost, this relationship forms a U-shaped curve, where the SHAP values are lowest at the mid-range RPG levels and increase at both the low and high extremes. In contrast, CatBoost displays an S-shaped curve, with SHAP values rising sharply from the mid-low RPG levels and then plateauing at higher levels.

**
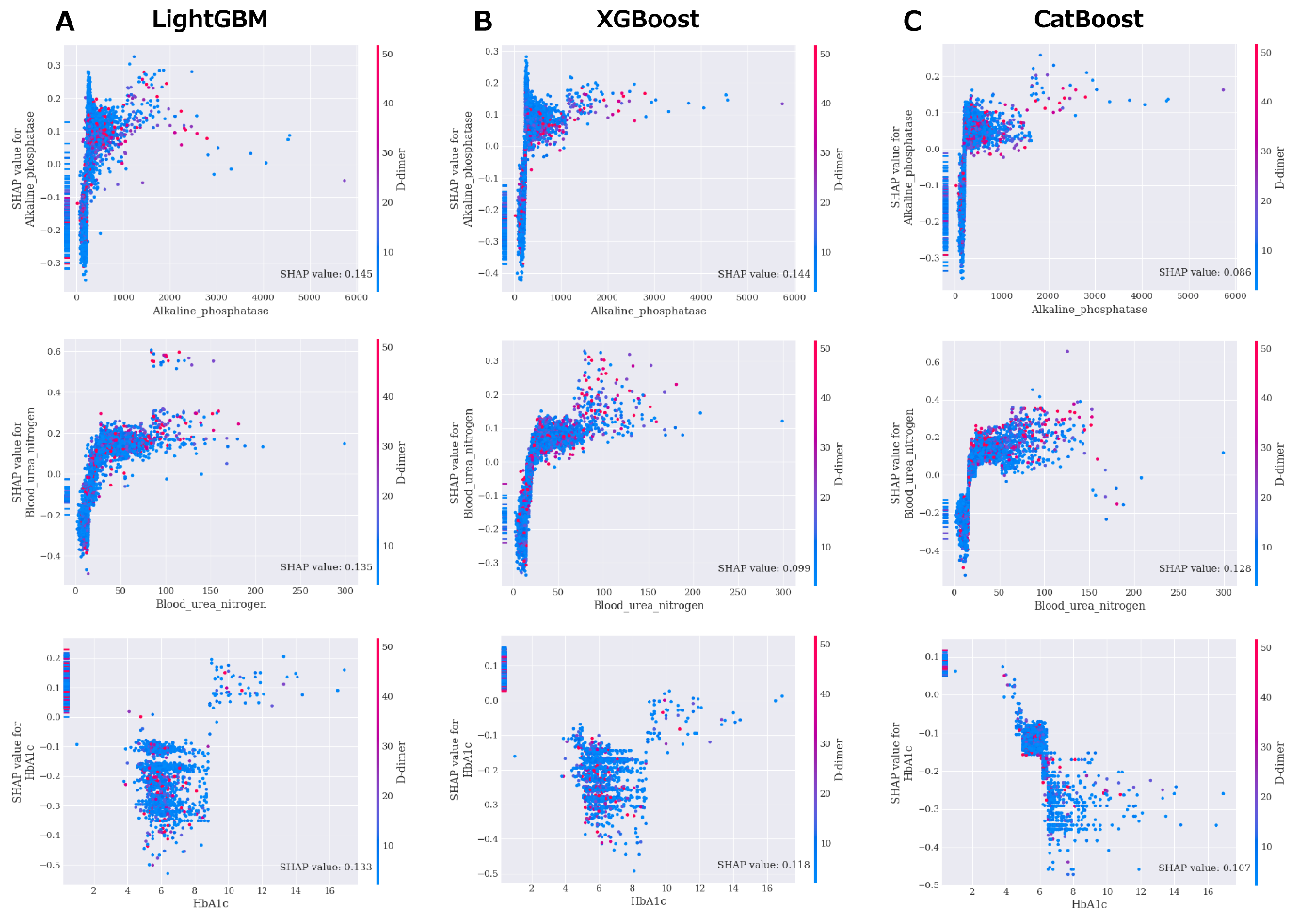
**

**Supplementary Figure 5. SHAP Dependence Plots for ALP, BUN, and HbA1c**

**Alkaline Phosphatase (ALP)**: ALP levels exhibit a square-root logarithmic curve with the SHAP values across all models, where the mid-range ALP values are associated with higher SHAP values.

**Blood urea nitrogen (BUN)**: BUN levels exhibit a square-root logarithmic curve with the SHAP values across all models, where the mid-low range BUN values are associated with higher SHAP values. XGBoost displays this relationship more gradually, while LightGBM and CatBoost show a more pronounced square-root logarithm, emphasizing a sharper increase in predicted risk at the mid-low range BUN levels.

**HbA1c:** HbA1c levels exhibit a nonlinear relationship with SHAP values in LightGBM and XGBoost. In contrast, CatBoost shows a weak negative linear relationship.

**
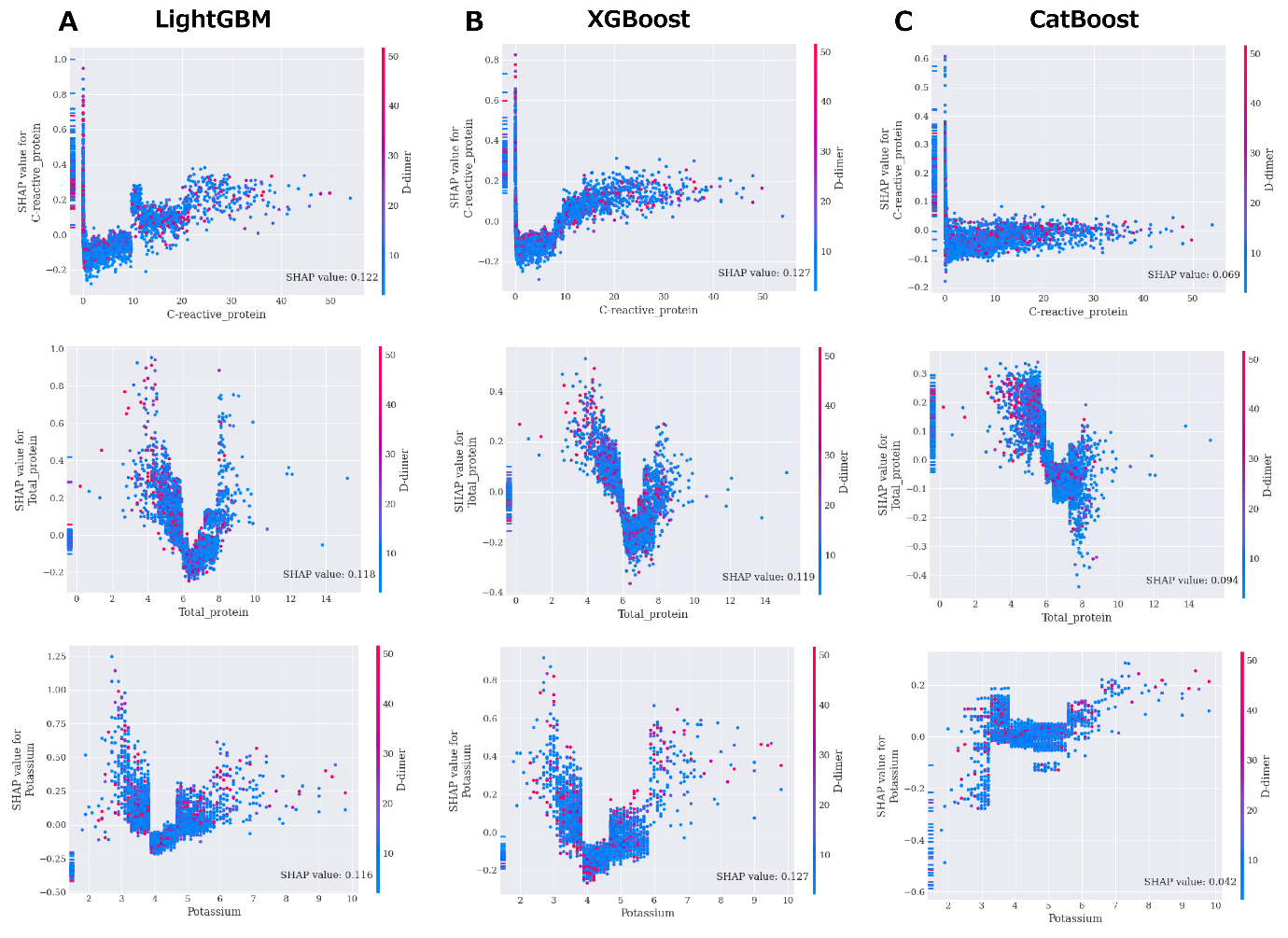
**

**Supplementary Figure 6. SHAP Dependence Plots for CRP, Total protein, and Potassium**

**C-reactive protein (CRP)**: In all models, CRP levels exhibit an L-shaped nonlinear relationship with the SHAP values. In LightGBM and XGBoost, the SHAP values show a gradual increase as CRP levels rise beyond the normal upper limit. In contrast, CatBoost displays an L-shaped curve where the SHAP values plateau, remaining almost constant at higher CRP levels.

**Total protein:** In LightGBM and XGBoost, TP levels display a U-shaped nonlinear relationship with the SHAP values, where the SHAP values reach their lowest point around 6 g/dL, with both lower and higher TP levels contributing to increased predicted risk. In contrast, CatBoost exhibits a weak negative correlation rather than a U-shape, suggesting a slight decrease in predicted risk as TP levels increase.

**Potassium:** In LightGBM and XGBoost, potassium levels display a U-shaped nonlinear relationship with the SHAP values, where the SHAP values reach their lowest point of approximately 4.0 mEq/L, with both lower and higher potassium levels contributing to increased predicted risk. In CatBoost, potassium levels exhibit a pattern similar to an inverse L-shape in their relationship with the SHAP values. The SHAP values are higher at very low potassium levels (below 3.5 mmol/L) and remain relatively stable around zero for potassium levels between 3.5 and 5 mmol/L. Beyond 5 mmol/L, the SHAP values rise, indicating that both low and high potassium levels are associated with increased predicted risk.

**
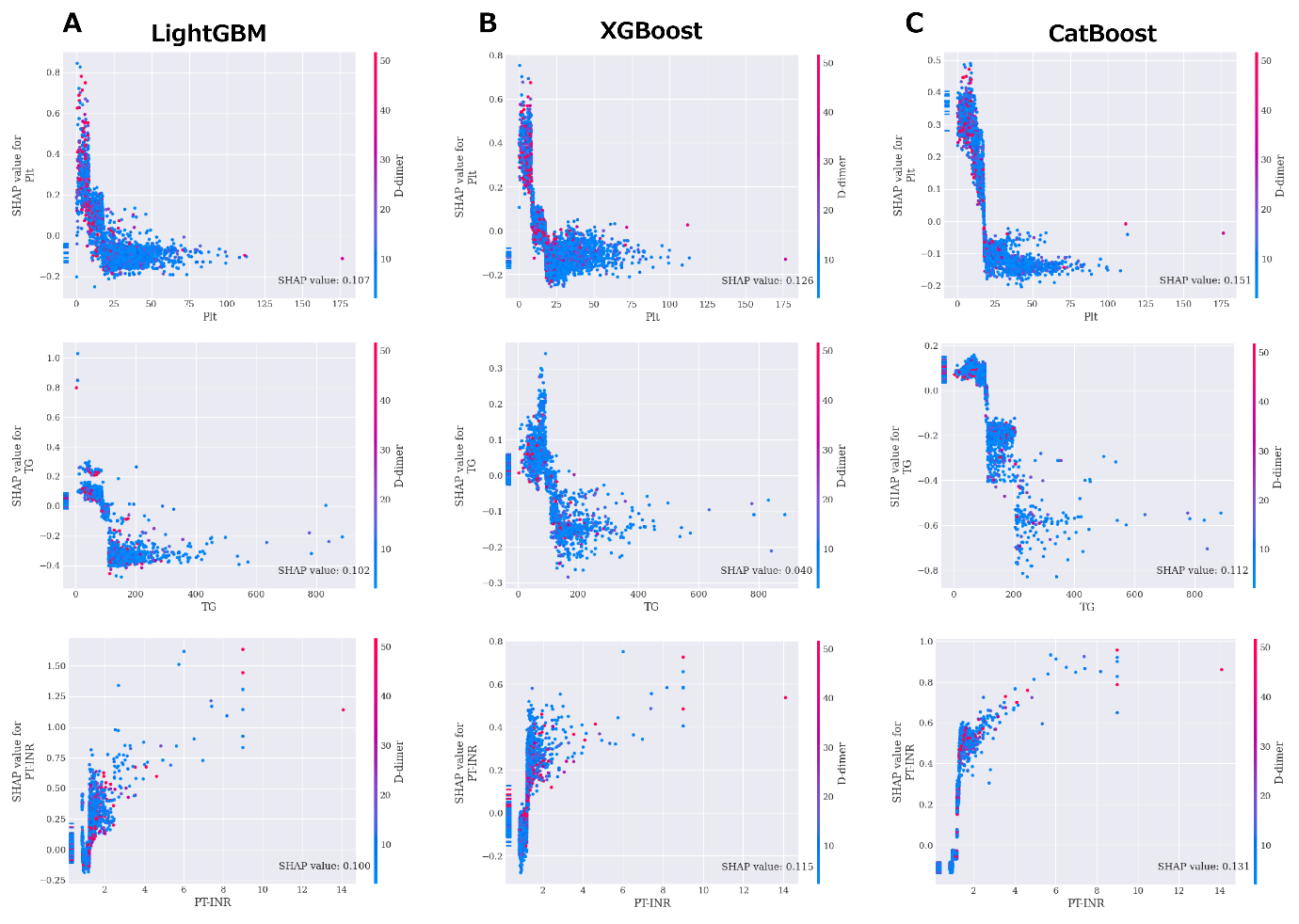
Supplementary Figure 7. SHAP Dependence Plots for Platelet counts, TG, and PT-INR**

**Platelet counts (Plt)**: Platelet counts exhibit an L-shaped relationship with the SHAP values across all models, where higher platelet counts are associated with decreased SHAP values. This pattern suggests that lower platelet levels contribute more significantly to the predicted risk across LightGBM, XGBoost, and CatBoost.

**Triglyceride (TG)**: In LightGBM and XGBoost, TG levels exhibit an initial sharp decrease in the SHAP values at lower concentrations, followed by a stable plateau at higher TG levels. This pattern suggests that while elevated TG levels may initially correspond to a reduced predicted risk, this association stabilizes once TG reaches moderate levels, showing no further significant impact on the SHAP values. This relationship deviates from a simple linear trend, reflecting a more complex interaction between TG levels and predicted risk in these models. In XGBoost, triglyceride (TG) levels show a more complex pattern compared with LightGBM and CatBoost. The SHAP values generally decrease as TG levels increase, indicating a reduction in predicted risk with higher TG levels. However, the relationship is not strictly linear; The SHAP values fluctuate more at lower TG levels, stabilizing at a negative SHAP value as TG levels reach approximately 200 mg/dL and beyond. This suggests that lower TG levels have a variable effect on risk, while higher TG levels contribute to a modestly reduced risk in a more stable manner.

**PT-INR:** Higher PT-INR levels show a positive correlation with the SHAP values across all models. In LightGBM, the relationship exhibits a gradual positive increase, while in XGBoost and CatBoost, the pattern resembles a square-root logarithmic curve.

**
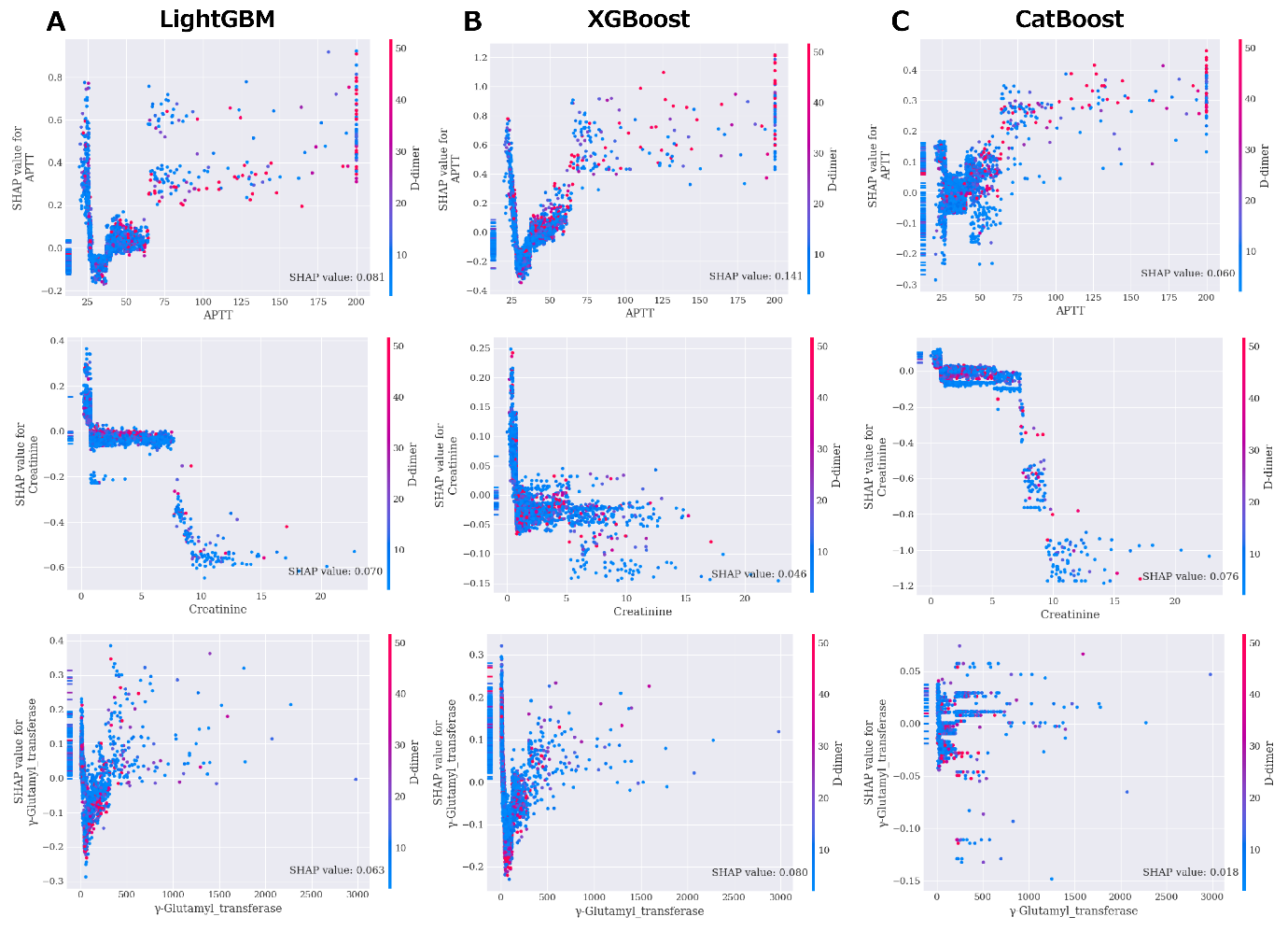
Supplementary Figure 8. SHAP Dependence Plots for APTT, Creatinine, and γ-GTP**

**Activated Partial Thromboplastin Time (APTT):** In LightGBM and XGBoost, APTT levels exhibit a V-shaped nonlinear relationship with the SHAP values, where the SHAP values reach their lowest point around 30 s. Both lower and higher APTT levels contribute to an increased predicted risk, reflecting a V-shaped risk profile associated with abnormal APTT values. In contrast, CatBoost shows a more complex, gradual increase in the SHAP values as APTT levels rise, indicating a less pronounced nonlinear relationship and a tendency for elevated APTT levels to contribute positively to risk predictions without the distinct V-shaped curve observed in the other models.

**Creatinine:** In LightGBM and CatBoost, creatinine levels show a two-tiered, L-shaped pattern in their relationship with the SHAP values. At low creatinine levels (around 1 or below), the SHAP values are relatively high, indicating a positive impact on the predicted risk. As creatinine levels increase to approximately 1.0 mg/dL, the SHAP values stabilize, creating a plateau. Beyond a creatinine level of approximately 7, the SHAP values begin to decrease in a gradual downward trend, reflecting a reduced contribution to risk prediction at higher creatinine levels. This pattern suggests a nonlinear relationship where low creatinine levels are associated with higher risk, moderate levels indicate stable risk, and elevated levels suggest a diminished contribution to risk. In XGBoost, a similar two-tiered pattern is observed, with the SHAP values initially high at low creatinine levels, stabilizing around moderate levels, and then showing a gradual decline as creatinine levels increase.

**γ-Glutamyl transpeptidase (γ-GTP):** In LightGBM and XGBoost, the SHAP dependence plots for γ-GTP levels indeed show a V-shaped pattern. This indicates a non-linear relationship, where both low and high γ-GTP levels are associated with increased SHAP values, suggesting an elevated predicted risk at these extremes. In contrast, CatBoost displays a relatively flat trend, with the SHAP values showing minimal variation across the range of γ-GTP levels, suggesting that γ-GTP contributes less significantly to risk prediction in this model.
