# Supplementary material for: Routine Laboratory Tests Predict 72‐h Fatality in Patients With D‐Dimer Levels ≥ 2 μg/mL: A Retrospective Cohort Study Comparing Statistical and Machine Learning Models
Source: J Clin Lab Anal. 2025 Sep 3;39(18):e70091. doi: 10.1002/jcla.70091 (PMC12459218; doi:10.1002/jcla.70091)
Supplement: Supplementary file 4 — DATA S4: jcla70091‐sup‐0004‐Tables.docx. TABLE S1: Patient demographics, and laboratory test values of training dataset. TABLE S2: Patient demographics, and laboratory test values of validation dataset. TABLE S3: Univariate and multivariate logistic regression analysis results. TABLE S4: Comparison of AUC, 95% CI, sensitivity, specificity, accuracy, precision, recall, F1, log loss, MCC, and Cohen's Kappa. TABLE S5: Degree of contribution of variables and the most contributive ranges to 72‐h outcomes of prediction one. TABLE S6: Bootstrap statistics among MLRA, LightGBM, XGBoost, CatBoost. TABLE S7: Comparison of statistics for the regression lines of the calibration plot. [file JCLA-39-e70091-s002.docx]

**Supplementary Tables**

**Supplementary Table 1**. Patient demographics, and laboratory test values of training dataset

| **Variables** | **Unit** | **Total number** |  | **72-hour outcome** | |  |  |
| --- | --- | --- | --- | --- | --- | --- | --- |
|  |  |  | **Death (*n* = 241)**  **Me** (2.5–97.5%) | **Number** | **Survival (*n* = 4917)**  **Me** (2.5–97.5%) | **Number** | ***P*-value** |
| Age | years | 5158 | 77.0 (40.0–94.5) | 241 | 74.0 (30.0–92.0) | 4917 | 0.000 |
| Men, proportion | % | 5158 | 51.9 | 125 | 58.0 | 2853 | 0.059 |
| **Complete blood count** |  |  |  |  |  |  |  |
| White blood cells | 10^3^/μL | 5131 | 11.020 (1.402–42.524) | 241 | 7.850 (2.755–22.860) | 4890 | 0.000 |
| Red blood cells | 10^4^/μL | 5131 | 346.0 (156.4–506.9) | 241 | 371.0 (223.0–515.0) | 4890 | 0.000 |
| Hemoglobin | g/dL | 5131 | 10.70 (5.41–15.84) | 241 | 11.30 (6.90–15.80) | 4890 | 0.000 |
| Hematocrit | % | 5131 | 32.80 (16.42–47.12) | 241 | 34.70 (21.20–47.40) | 4890 | 0.000 |
| Platelet count | 10^4^/μL | 5131 | 15.70 (1.66–48.24) | 241 | 21.00 (4.10–55.00) | 4890 | 0.000 |
| **Biochemical examinations** |  |  |  |  |  |  |  |
| Total protein | g/dL | 4925 | 5.80 (3.20–8.300) | 235 | 6.50 (4.30–8.20) | 4690 | 0.000 |
| Albumin | g/dL | 4925 | 2.70 (1.20–4.60) | 235 | 3.30 (1.70–4.60) | 4690 | 0.000 |
| Total bilirubin | mg/dL | 5057 | 0.630 (0.145–10.524) | 241 | 0560 (0.190–3.273) | 4816 | 0.000 |
| Direct bilirubin | mg/dL | 5057 | 0.280 (0.060–8.590) | 241 | 0.210 (0.070–2.000) | 4816 | 0.000 |
| Aspartate aminotransferase | U/L | 5116 | 48.0 (10.5–4452.2) | 241 | 24.0 (9.0–484.4) | 4875 | 0.000 |
| Alanine aminotransferase | U/L | 5116 | 30.0 (5.5–1561.9) | 241 | 18.0 (4.0–325.0) | 4875 | 0.000 |
| γ-Glutamyl transferase | U/L | 4625 | 34.0 (9.0–547.9) | 231 | 31.0 (10.0–380.0) | 4394 | 0.082 |
| Lactate dehydrogenase | U/L | 5053 | 383.0 (146.5–6894.8) | 239 | 251.0 (141.0–1087.6) | 4796 | 0.000 |
| Alkaline phosphatase | U/L | 4887 | 284.5 (102.5–1619.9) | 238 | 253.0 (124.0–952.3) | 4649 | 0.000 |
| Creatine kinase | U/L | 4756 | 131.0 (21.0–11231.2) | 231 | 85.0 (17.0–2371.8) | 4525 | 0.000 |
| Amylase | U/L | 1737 | 88.5 (14.8–1314.4) | 92 | 73.0 (21.0–413.5) | 1645 | 0.020 |
| C-reactive protein | mg/dL | 4956 | 3.525 (0.030–32.772) | 228 | 1.380 (0.030–26.244) | 4728 | 0.000 |
| Sodium | mEq/L | 5090 | 139.0 (121.5–154.0) | 241 | 139.0 (128.7–147.0) | 4849 | 0.143 |
| Potassium | mEq/L | 5090 | 4.50 (2.90–7.54) | 241 | 4.30 (3.10–5.80) | 4849 | 0.000 |
| Chloride | mEq/L | 5058 | 104.0 (87.5–118.0) | 241 | 104.0 (93.0–113.0) | 4848 | 0.039 |
| Calcium | mg/dL | 4300 | 8.30 (6.07–11.10) | 234 | 8.60 (6.90–10.00) | 4066 | 0.000 |
| Inorganic phosphorus | mg/dL | 4303 | 4.30 (1.74–10.60) | 234 | 3.40 (1.80–7.90) | 4069 | 0.000 |
| Magnesium | mg/dL | 3168 | 2.10 (1.50–3.80) | 195 | 2.00 (1.40–3.00) | 2973 | 0.000 |
| Blood urea nitrogen | mg/dL | 5106 | 30.00 (9.00–132.50) | 240 | 19.00 (7.00–86.00) | 4866 | 0.000 |
| Creatinine | mg/dL | 5113 | 1.165 (0.420–7.115) | 240 | 0.840 (0.380–8.250) | 4873 | 0.000 |
| Uric acid | mg/dL | 3732 | 6.60 (2.47–15.65) | 173 | 5.50 (2.10–11.95) | 3559 | 0.000 |
| Total cholesterol | mg/dL | 1752 | 142.0 (57.2–246.5) | 63 | 164.0 (88.7–275.3) | 1689 | 0.000 |
| Triglyceride | mg/dL | 1937 | 77.5 (8.0–227.0) | 68 | 96.0 (35.0–305.0) | 1869 | 0.001 |
| HDL-C | mg/dL | 1585 | 38.0 (3.0–86.2) | 39 | 43.0 (17.0–85.9) | 1546 | 0.043 |
| LDL-C | mg/dL | 1005 | 80.00 (27.03–150.40) | 21 | 92.00 (38.00–178.00) | 984 | 0.046 |
| Random plasma glucose | mg/dL | 3169 | 153.0 (35.1–545.5) | 163 | 122.0 (74.0–330.0) | 3006 | 0.000 |
| HbA1c | % | 1711 | 5.90 (4.90–9.67) | 39 | 6.00 (4.83–9.57) | 1672 | 0.746 |
| Serum iron | μg/dL | 464 | 86.5 (11.0–522.0) | 10 | 40.0 (7.0–212.1) | 454 | 0.052 |
| Ferritin | ng/mL | 346 | 2025.60 (174.20–17079.60) | 7 | 165.30 (8.38–3215.46) | 339 | 0.002 |
| **Coagulation examination** |  |  |  |  |  |  |  |
| PT-INR |  | 4543 | 1.150 (0.870–7.037) | 230 | 1.040 (0.870–1.907) | 4313 | 0.000 |
| APTT | sec | 4562 | 38.20 (23.40–200.00) | 234 | 32.90 (24.40–74.93) | 4328 | 0.000 |
| Fibrinogen | mg/dL | 2234 | 327.0 (73.6–768.6) | 159 | 373.0 (126.0–931.3) | 2075 | 0.000 |
| Antithrombin Ⅲ | % | 543 | 63.0 (17.4–114.8) | 27 | 84.0 (40.0–129.6) | 516 | 0.000 |
| FDP | μg/mL | 1687 | 38.90 (5.56–525.56) | 104 | 15.00 (5.11–273.78) | 1583 | 0.000 |
| D-dimer | μg/mL | 5158 | 11.70 (2.31–157.24) | 241 | 5.30 (2.00–79.19) | 4917 | 0.000 |

*P*-values were calculated using Fisher’s exact test for categorical variables and the Mann–Whitney *U*-test for continuous variables*.*

HDL-C, high-density lipoprotein cholesterol; LDL-C, low-density lipoprotein cholesterol, PT-INR, prothrombin time - international normalized ratio, APTT: activated partial thromboplastin time; FDP, fibrin/fibrinogen degradation product

**Supplementary Table 2**. Patient demographics, and laboratory test values of validation dataset

| **Variables** | **Unit** | **Total number** | **72-hour outcome** | | | | |
| --- | --- | --- | --- | --- | --- | --- | --- |
|  |  |  | **Death (*n* = 309)**  **Me** (2.5~97.5%) | **Number** | **Survival (*n* = 5241)**  **Me** (2.5~97.5%) | **Number** | ***P*-value** |
| Age | years | 5550 | 79.0 (43.2–94.8) | 309 | 75.0 (32.5–92.5) | 5341 | 0.000 |
| Men, proportion | % | 3215 | 59.5 | 184 | 57.8 | 3031 | 0.055 |
| **Complete blood count** |  |  |  |  |  |  |  |
| White blood cells | 10^3^/μL | 5518 | 10.760 (0.452–51.829) | 307 | 8.080 (2.690–23.517) | 5211 | 0.000 |
| Red blood cells | 10^4^/μL | 5518 | 346.0 (156.4–506.9) | 307 | 371.0 (223.0–515.0) | 5211 | 0.000 |
| Hemoglobin | g/dL | 5518 | 10.70 (5.41–15.84) | 307 | 11.30 (6.90–15.80) | 5211 | 0.000 |
| Hematocrit | % | 5518 | 32.80 (16.42–47.12) | 307 | 34.70 (21.20–47.40) | 5211 | 0.003 |
| Platelet count | 10^4^/μL | 5517 | 15.70 (1.66–48.24) | 307 | 21.00 (4.10–55.00) | 5210 | 0.000 |
| **Biochemical examinations** |  |  |  |  |  |  |  |
| Total protein | g/dL | 5435 | 5.90 (2.82~7.89) | 304 | 6.50 (4.30~8.00) | 5131 | 0.000 |
| Albumin | g/dL | 5433 | 2.70 (1.20~4.50) | 304 | 3.20 (1.70~4.50) | 5129 | 0.000 |
| Total bilirubin | mg/dL | 5453 | 0.770 (0.171~16.855) | 305 | 0.580 (0.210~3.322) | 5148 | 0.000 |
| Direct bilirubin | mg/dL | 5454 | 0.330 (0.071~12.078) | 305 | 0.210 (0.070~2.090) | 5149 | 0.000 |
| Aspartate aminotransferase | U/L | 5498 | 46.0 (15.0~2527.6) | 308 | 26.5 (12.0~371.5) | 5190 | 0.000 |
| Alanine aminotransferase | U/L | 5498 | 31.0 (7.0~1302.6) | 308 | 20.0 (6.0~254.3) | 5190 | 0.000 |
| γ-Glutamyl transferase | U/L | 5117 | 33.0 (9.0~545.1) | 297 | 32.0 (9.0~450.0) | 4820 | 0.278 |
| Lactate dehydrogenase | U/L | 5442 | 370.0 (164.4~5133.4) | 308 | 258.0 (138.0~1084.6) | 5134 | 0.000 |
| Alkaline phosphatase | U/L | 4177 | 255.0 (116.2~1034.2) | 229 | 252.0 (120.0~1026.2) | 3948 | 0.174 |
| Creatine kinase | U/L | 5012 | 132.0 (18.7~4792.8) | 286 | 84.0 (15.0~2187.1) | 4726 | 0.000 |
| Amylase | U/L | 1649 | 92.5 (20.7~927.9) | 94 | 74.0 (21.0~538.6) | 1555 | 0.009 |
| C-reactive protein | mg/dL | 5378 | 4.670 (0.030~38.502) | 301 | 1.900 (0.030~26.662) | 5077 | 0.000 |
| Sodium | mEq/L | 5479 | 139.0 (125.0~155.6) | 309 | 139.0 (128.0~148.0) | 5170 | 0.061 |
| Potassium | mEq/L | 5478 | 4.50 (2.57~7.68) | 309 | 4.20 (3.10~5.70) | 5169 | 0.000 |
| Chloride | mEq/L | 5475 | 104.0 (88.0~120.0) | 309 | 104.0 (92.0~114.0) | 5166 | 0.720 |
| Calcium | mg/dL | 4653 | 8.30 (6.20~10.00) | 296 | 8.50 (7.00~9.80) | 4357 | 0.000 |
| Inorganic phosphorus | mg/dL | 4650 | 4.60 (1.60~12.10) | 296 | 3.40 (1.80~7.40) | 4354 | 0.000 |
| Magnesium | mg/dL | 3562 | 2.20 (1.50~3.86) | 235 | 2.00 (1.40~3.00) | 3327 | 0.000 |
| Blood urea nitrogen | mg/dL | 5488 | 32.50 (10.00~134.60) | 308 | 20.00 (7.00~86.00) | 5180 | 0.000 |
| Creatinine | mg/dL | 5490 | 1.260 (0.370~9.464) | 308 | 0.840 (0.380~7.507) | 5182 | 0.000 |
| Uric acid | mg/dL | 4012 | 6.50 (2.10~17.22) | 212 | 5.40 (2.00~11.95) | 3800 | 0.000 |
| Total cholesterol | mg/dL | 1829 | 150.0 (42.1~252.6) | 75 | 164.0 (86.0~276.7) | 1754 | 0.000 |
| Triglyceride | mg/dL | 1957 | 89.0 (29.6~274.8) | 73 | 95.0 (36.6~320.0) | 1884 | 0.201 |
| HDL-C | mg/dL | 1529 | 34.0 (8.8~73.4) | 51 | 45.0 (17.0~90.6) | 1478 | 0.043 |
| LDL-C | mg/dL | 1021 | 80.00 (35.73~165.53) | 29 | 89.50 (33.30~174.00) | 992 | 0.000 |
| Random plasma glucose | mg/dL | 3423 | 156.5 (32.0~453.0) | 190 | 122.0 (73.0~333.7) | 3233 | 0.000 |
| HbA1c | % | 1694 | 6.15 (4.80~9.50) | 36 | 6.00 (4.90~9.50) | 1658 | 0.338 |
| PT-INR |  | 5198 | 1.200 (0.901~4.078) | 304 | 1.020 (0.860~1.964) | 4894 | 0.000 |
| APTT | sec | 3291 | 38.20 (23.40–200.00) | 173 | 89.0 (40.2~126.0) | 3118 | 0.000 |
| Fibrinogen | mg/dL | 2504 | 288.0 (50.0~1048.3) | 175 | 380.0 (128.7~924.0) | 2329 | 0.000 |
| Antithrombin Ⅲ | % | 502 | 67.0 (29.3~109.7) | 33 | 89.0 (40.2~126.0) | 469 | 0.000 |
| FDP | μg/mL | 1477 | 29.35 (5.35~790.51) | 96 | 14.30 (5.20~316.26) | 1381 | 0.000 |
| D-dimer | μg/mL | 5550 | 11.90 (2.20~290.33) | 309 | 5.30 (2.00~74.79) | 5241 | 0.000 |
| Serum iron | μg/dL | 234 | NA | 2 | 43.5 (8.3~213.6) | 232 | 0.152 |
| Ferritin | ng/mL | 373 | 1124.80 (15.00~124930.00) | 7 | 172.90 (7.48~4377.56) | 366 | 0.043 |

*P*-values were calculated using Fisher’s exact test for categorical variables and the Mann–Whitney *U*-test for continuous variables*.*

HDL-C, high-density lipoprotein cholesterol; LDL-C, low-density lipoprotein cholesterol, PT-INR, prothrombin time - international normalized ratio, APTT: activated partial thromboplastin time; FDP, fibrin/fibrinogen degradation products

**Supplementary Table 3**. **Univariate and multivariate logistic regression analysis results**

|  | **Univariate logistic regression analysis** | | | **Multivariate logistic regression analysis** | | | | |
| --- | --- | --- | --- | --- | --- | --- | --- | --- |
|  | **Crude OR (95% CI)** | ***P*-value** | **AUC** | **Adjusted OR (95% CI) by age, and sex** | ***P*-value** | **AUC** | **AIC** | ***n* (death: survival)** |
| Age | 1.020 (1.010–1.030) | 0.0001 | 0.582 | N/A | N/A | N/A | N/A | N/A |
| Men, proportion | 0.780 (0.602–1.010) | 0.0595 | 0.531 | N/A | N/A | N/A | N/A | N/A |
| **Complete blood count** | | | | | | | | |
| White blood cells | 2.183 (1.745–2.730) | 0.0000 | 0.649 | 2.328 (1.853–2.924) | 0.0000 | 0.649 | 1881.0 | 5131(241:4890) |
| Red blood cells | 0.995 (0.994–0.997) | 0.0000 | 0.585 | 0.996 (0.994–0.997) | 0.0000 | 0.585 | 1908.5 | 5131(241:4890) |
| Hemoglobin | 0.881 (0.833–0.931) | 0.0000 | 0.570 | 0.891 (0.841–0.943) | 0.0001 | 0.570 | 1916.2 | 5131(241:4890) |
| Hematocrit | 0.959 (0.941–0.978) | 0.0000 | 0.565 | 0.962 (0.944–0.981) | 0.0001 | 0.565 | 1917.2 | 5131(241:4890) |
| Platelet count | 0.850 (0.816–0.884) | 0.0000 | 0.642 | 0.846 (0.812–0.881) | 0.0000 | 0.642 | 1865.1 | 5131(241:4890) |
| **Biochemical examinations** | | | | | | | | |
| Total protein | 0.438 (0.368–0.521) | 0.0000 | 0.649 | 0.428 (0.358–0.511) | 0.0000 | 0.649 | 1790.25 | 4925(235:4690) |
| Albumin | 0.484 (0.411–0.570) | 0.0000 | 0.651 | 0.484 (0.410–0.572) | 0.0000 | 0.651 | 1801.99 | 4925(235:4690) |
| Total bilirubin | 1.517 (1.295–1.777) | 0.0000 | 0.572 | 1.556 (1.322–1.831) | 0.0000 | 0.572 | 1898.40 | 5057(241:4816) |
| Direct bilirubin | 1.591 (1.403–1.803) | 0.0000 | 0.601 | 1.641 (1.443–1.866) | 0.0000 | 0.601 | 1874.78 | 5057(241:4816) |
| Aspartate aminotransferase | 1.775 (1.626–1.937) | 0.0000 | 0.720 | 1.823 (1.667–1.993) | 0.0000 | 0.720 | 1780.76 | 5116(241:4875) |
| Alanine aminotransferase | 1.545 (1.408–1.694) | 0.0000 | 0.644 | 1.616 (1.471–1.774) | 0.0000 | 0.644 | 1842.93 | 5116(241:4875) |
| γ-Glutamyl transferase | 1.190 (1.048–1.352) | 0.0073 | 0.534 | 1.296 (1.137–1.477) | 0.0001 | 0.534 | 1809.84 | 4625(231:4394) |
| Lactate dehydrogenase | 2.845 (2.439–3.317) | 0.0000 | 0.734 | 2.977 (2.543–3.484) | 0.0000 | 0.734 | 1744.08 | 5053(239:4796) |
| Alkaline phosphatase | 1.722 (1.385–2.141) | 0.0000 | 0.586 | 1.748(1.402–2.178) | 0.0000 | 0.586 | 1867.40 | 4887(238:4649) |
| Creatine kinase | 1.368 (1.252–1.496) | 0.0000 | 0.611 | 1.407 (1.285–1.540) | 0.0000 | 0.611 | 1789.07 | 4756(231:4525) |
| Amylase | 1.568 (1.235–1.990) | 0.0002 | 0.572 | 1.553 (1.220–1.979) | 0.0004 | 0.572 | 551.64 | 1737 (92:1645) |
| C-reactive protein | 1.212 (1.116–1.316) | 0.0000 | 0.587 | 1.218 (1.121–1.324) | 0.0000 | 0.587 | 1811.43 | 4956(228:4728) |
| Sodium | 0.079 (0.006–0.991) | 0.0492 | 0.528 | 0.075 (0.006–0.917) | 0.0426 | 0.528 | 1924.38 | 506 (241:4849) |
| Potassium | 10.363 (4.899–21.922) | 0.0000 | 0.578 | 10.269 (4.837–21.803) | 0.0000 | 0.578 | 1893.38 | 506 (241:4849) |
| Chloride | 0.970 (0.947–0.993) | 0.0122 | 0.539 | 0.970 (0.947–0.993) | 0.0108 | 0.539 | 1921.92 | 505 (241:4848) |
| Calcium | 0.779 (0.669–0.907) | 0.0013 | 0.584 | 0.771 (0.660–0.900) | 0.0010 | 0.584 | 1796.0 | 4300(234:4066) |
| Inorganic phosphorus | 5.190 (3.742–7.198) | 0.0000 | 0.668 | 5.327 (3.823–7.421) | 0.0000 | 0.668 | 1714.6 | 4303(234:4069) |
| Magnesium | 5.999 (3.173–11.342) | 0.0000 | 0.603 | 5.776 (3.047–10.947) | 0.0000 | 0.603 | 1433.2 | 3168(195:2973) |
| Blood urea nitrogen | 2.564 (2.127–3.092) | 0.0000 | 0.676 | 2.570 (2.117–3.119) | 0.0000 | 0.676 | 1836.5 | 5106(240:4866) |
| Creatinine | 1.479 (1.266–1.728) | 0.0000 | 0.610 | 1.531 (1.301–1.801) | 0.0000 | 0.610 | 1900.1 | 5113(240:4873) |
| Uric acid | 2.784 (1.912–4.053) | 0.0000 | 0.613 | 2.852 (1.950–4.170) | 0.0000 | 0.613 | 1361.9 | 3732(173:3559) |
| Total cholesterol | 0.112 (0.052–0.241) | 0.0000 | 0.638 | 0.089 (0.041–0.196) | 0.0000 | 0.638 | 509.09 | 1752 (63:1689) |
| Triglyceride | 0.436 (0.298–0.638) | 0.0000 | 0.617 | 0.445 (0.302–0.656) | 0.0000 | 0.617 | 573.03 | 1937 (68:1869) |
| HDL-C | 0.977 (0.957–0.998) | 0.0282 | 0.595 | 0.974 (0.953–2.771) | 0.0137 | 0.595 | 365.78 | 1585 (39:1546) |
| LDL-C | 0.297 (0.107–0.726) | 0.0089 | 0.627 | 0.235 (0.089–0.621) | 0.0035 | 0.627 | 202.43 | 1005 (21:984) |
| Random plasma glucose | 1.323 (1.176–1.488) | 0.0000 | 0.607 | 1.321 (1.173–1.488) | 0.0000 | 0.607 | 1265.3 | 3169(163:3006) |
| HbA1c | 1.215 (0.403–3.662) | 0.7293 | 0.515 | 1.239 (0.396–3.874) | 0.7127 | 0.515 | 376.54 | 1711 (39:1672) |
| Serum iron | 2.454 (1.138–5.290) | 0.0220 | 0.679 | 2.670 (1.180–6.043) | 0.0185 | 0.679 | 96.560 | 464 (10:454) |
| Ferritin | 2.402 (1.539–3.748) | 0.0001 | 0.846 | 3.151 (1.789–5.553) | 0.0001 | 0.846 | 50.730 | 346 (7:339) |
| **Coagulation examinations** | |  |  |  |  |  |  |  |
| PT-INR | 2.86 0(2.358–3.469) | 0.0000 | 0.670 | 2.896 (2.385–3.517) | 0.0000 | 0.670 | 1708.2 | 4543(230:4313) |
| APTT | 3.187 (2.608–3.896) | 0.0000 | 0.641 | 3.287 (2.685–4.024) | 0.0000 | 0.641 | 1723.5 | 4562(234:4328) |
| Fibrinogen | 0.483 (0.360–0.646) | 0.0000 | 0.601 | 0.472 (0.351–0.635) | 0.0000 | 0.601 | 1112.1 | 2234(159:2075) |
| Antithrombin Ⅲ | 0.964 (0.947–0.980) | 0.0000 | 0.715 | 0.964 (0.948–0.981) | 0.0000 | 0.715 | 200.84 | 543 (27:516) |
| FDP | 1.595 (1.365–1.864) | 0.0000 | 0.652 | 1.600 (1.368–1.873) | 0.0000 | 0.652 | 745.18 | 1687(104:1583) |
| D-dimer | 1.735 (1.564–1.925) | 0.0000 | 0.681 | 1.738 (1.565–1.931) | 0.0000 | 0.681 | 1837.6 | 5212(241:4971) |

**Abbreviations**: OR: Odds ratio, CI: Confidence interval, AUC: area under the curve, AIC: Akaike's Information Criterion, HDL-C, high-density lipoprotein cholesterol; LDL-C, low-density lipoprotein cholesterol, PT-INR, prothrombin time - international normalized ratio, APTT: activated partial thromboplastin time; FDP, fibrin/fibrinogen degradation products.

To calculate the odds ratio, the variations were 1.0.

**Supplementary Table 4. Comparison of AUC, 95%CI, Sensitivity, Specificity, Accuracy, Precision, Recall, F1, Log Loss, MCC, and Cohen’s Kappa**

| Analysis (1) Training dataset | | | | | | | | | | | |
| --- | --- | --- | --- | --- | --- | --- | --- | --- | --- | --- | --- |
|  | **AUC** | **95%CI of AUC** | **Sensitivity** | **Specificity** | **Accuracy** | **Precision** | **Recall** | **F1 score** | **1-Log Loss** | **MCC** | **Cohen’s Kappa** |
| MLRA | 0.829 | 0.768, 0.888 | 0.762 | 0.809 | 0.807 | 0.128 | 0.746 | 0.218 | 0.876 | 0.254 | 0.167 |
| Prediction One | 0.814 | 0.781, 0.847 | 0.822 | 0.678 | 0.685 | 0.111 | 0.822 | 0.196 | 0.843 | 0.222 | 0.124 |
| LightGBM | 0.987 | 0.977, 0.997 | 0.987 | 0.911 | 0.914 | 0.352 | 0.988 | 0.519 | 0.921 | 0.561 | 0.483 |
| XGboost | 0.981 | 0.966, 0.992 | 0.967 | 0.896 | 0.899 | 0.313 | 0.967 | 0.473 | 0.916 | 0.518 | 0.433 |
| CatBoost | 0.937 | 0.916, 0.959 | 0.884 | 0.867 | 0.868 | 0.246 | 0.884 | 0.385 | 0.627 | 0.424 | 0.336 |

| Analysis (2) Validation dataset | | | | | | | | | | | |
| --- | --- | --- | --- | --- | --- | --- | --- | --- | --- | --- | --- |
|  | **AUC** | **95%CI of AUC** | **Sensitivity** | **Specificity** | **Accuracy** | **Precision** | **Recall** | **F1 score** | **1-Log Loss** | **MCC** | **Cohen’s Kappa** |
| MLRA | 0.788 | 0.735, 0.838 | 0.707 | 0.747 | 0.744 | 0.105 | 0.693 | 0.183 | 0.851 | 0.197 | 0.120 |
| Prediction One | 0.852 | 0.825, 0.879 | 0.809 | 0.743 | 0.747 | 0.157 | 0.809 | 0.263 | 0.829 | 0.280 | 0.187 |
| LightGBM | 0.848 | 0.821, 0.875 | 0.809 | 0.723 | 0.727 | 0.147 | 0.809 | 0.248 | 0.833 | 0.264 | 0.170 |
| XGboost | 0.852 | 0.828, 0.882 | 0.770 | 0.774 | 0.774 | 0.167 | 0.770 | 0.275 | 0.831 | 0.286 | 0.202 |
| CatBoost | 0.845 | 0.818, 0.873 | 0.761 | 0.786 | 0.784 | 0.173 | 0.761 | 0.282 | 0.582 | 0.291 | 0.210 |

AUC, area under the curve; CI, confidence interval; MCC, Matthews Correlation Coefficient.

**Supplementary Table 5**. **Degree of contribution of variables and the most contributive ranges to 72-hour outcomes of Prediction One™**

|  | **Variables** | **Total degree of contribution** | **Most contributing ranges for 72-hour fatalities** | **Most contributing ranges for 72-hour survival** |
| --- | --- | --- | --- | --- |
| 1 | Direct bilirubin (mg/dL) | 0.010044 | 2.3–23.8 | 0.2–0.3 |
| 2 | Aspartate transaminase (U/L) | 0.009854 | 100.7–15,100.0 | 1.0–13.1 |
| 3 | Calcium (mg/dL) | 0.009152 | 3.0–7.5 | 8.8–9.0 |
| 4 | Hematocrit (%) | 0.008989 | 8.3–25.7 | 36.4–38.2 |
| 5 | Magnesium (mg/dL) | 0.008487 | 2.7–7.7 | 2.1–2.3 |
| 6 | Lactate dehydrogenase (U/L) | 0.008373 | 432.2–740.8 | 14.0–170.0 |
| 7 | White blood cells (/μL) | 0.008171 | 17,100–377,900 | 9,270–10,554 |
| 8 | Blood urea nitrogen (mg/dL) | 0.008022 | 76.0–299.0 | 24.0–31.0 |
| 9 | Phosphorus (mg/dL) | 0.008020 | 4.8–6.8 | 3.7–4.1 |
| 10 | Albumin (g/dL) | 0.007930 | 0.1–2.2 | 2.6–2.9 |
| 11 | Red blood cell (×10^4^/μL) | 0.006968 | 41.0–272.1 | 371.0–391.0 |
| 12 | Total protein (g/dL) | 0.006860 | 0.2–5.1 | 5.7–6.1 |
| 13 | D-dimer (μg/mL) | 0.006853 | 16.3–33.4 | 5.6–7.4 |
| 14 | Uric acid (mg/dL) | 0.006811 | 8.3–11.3 | 7.2–8.3 |
| 15 | Alanine transaminase (U/L) | 0.006807 | 66.7–408.1 | 16.0–20.7 |
| 16 | Total bilirubin (mg/dL) | 0.006644 | 2.1– 31.9 | 0.9–1.2 |
| 17 | Creatine kinase (U/L) | 0.006438 | 352.6–1,943.8 | 1.0–36.2 |
| 18 | APTT (seconds) | 0.006368 | 53.5–200.0 | 33.4–35.4 |
| 19 | FDP (μg/mL) | 0.006214 | 113.6–2,397.0 | 12.7–16.6 |
| 20 | Chloride (mEq/L) | 0.006145 | 71.0–98.0 | 105.0–107.0 |
| 21 | Hb (g/dL) | 0.005902 | 2.9–8.3 | 10.8–11.5 |
| 22 | PT-INR | 0.005819 | 1.18–1.37 | 0.96–1.00 |
| 23 | Sodium (mEq/L) | 0.005766 | 143.0–192.0 | 139.0–140.0 |
| 24 | Amylase (U/L) | 0.005683 | 1.9–34.5 | 132.5–204.5 |
| 25 | C-reactive protein (mg/dL) | 0.005641 | 16.05–53.89 | 5.44–9.32 |
| 26 | AT-3 (%) | 0.005630 | 53.0–64.0 | 64.0–72.0 |
| 27 | γ-Glutamyl transferase (U/L) | 0.005514 | 223.1–2,977.0 | 61.2–96.0 |
| 28 | HDL-C (mg/dL) | 0.005494 | 2.0–26.0 | 75.0–135.0 |
| 29 | Total cholesterol (mg/dL) | 0.005256 | 39.0–112.0 | 210.0–243.0 |
| 30 | Platelet (/μL) | 0.005230 | 2,000–96,100 | 134,035–161,042 |
| 31 | Potassium (mEq/L) | 0.005143 | 5.6–9.8 | 4.4–4.6 |
| 32 | Fibrinogen (mg/dL) | 0.004971 | 333.1–372.0 | 25.0–198.0 |
| 33 | Alkaline phosphatase (U/L) | 0.004957 | 529.1–5,743.0 | 19.0–159.3 |
| 34 | Age (years) | 0.004918 | 86.0–94.0 | 59.0–67.0 |
| 35 | Random Plasma Glucose (mg/dL) | 0.004727 | 254.0–1,239.0 | N/A |

APTT, activated partial thromboplastin time; FDP, fibrin/fibrinogen degradation product; Hb, hemoglobin; PT-INR, prothrombin time-international normalized ratio; AT-3, antithrombin 3; HDL-C, high-density lipoprotein cholesterol.

**Supplementary Table 6. Bootstrap statistics among MLRA, LightGBM, XGBoost, CatBoost**

| Bootstrap statistics | MLRA | LightGBM | XGboost | CatBoost |
| --- | --- | --- | --- | --- |
| AUC Mean (95%CI) | 0.830 (0.770, 0.889) | 0.933 (0.918, 0.948) | 0.935 (0.920, 0.947) | 0.912 (0.900, 0.926) |
| Accuracy Mean (95%CI) | 0.802 (0.699, 0.877) | 0.968 (0.966, 0.971) | 0.967 (0.965, 0.970) | 0.875 (0.860, 0.889) |
| Sensitivity Mean (95%CI) | 0.749 (0.629, 0.870) | 0.831 (0.763, 0.896) | 0.839 (0.772, 0.904) | 0.841 (0.776, 0.900) |
| Specificity Mean (95%CI) | 0.804 (0.694, 0.885) | 0.895 (0.835, 0.942) | 0.885 (0.826, 0.937) | 0.848 (0.784, 0.900) |
| Precision Mean (95%CI) | 0.131 (0.080, 0.197) | 0.904 (0.842, 0.966) | 0.911 (0.851, 0.965) | 0.243 (0.224, 0.265) |
| F1 Score Mean (95%CI) | 0.221 (0.146, 0.309) | 0.511 (0.456, 0.565) | 0.491 (0.431, 0.542) | 0.372 (0.350, 0.397) |
| Apparent performance | 0.829 | 0.934 | 0.935 | 0.913 |
| Optimism | 0.000 | 0.016 | 0.014 | 0.013 |
| Corrected performance | 0.829 | 0.918 | 0.920 | 0.900 |
| Bias-corrected performance (AUC) | 0.829 | 0.918 | 0.920 | 0.900 |

AUC, area under the curve; CI, confidence interval

**Supplementary Table 7. Comparison of statistics for the regression lines of the calibration plot**

|  | MLRA | | Prediction One | | LightGBM | | XGboost | | | CatBoost | |
| --- | --- | --- | --- | --- | --- | --- | --- | --- | --- | --- | --- |
|  | **Training** | **Validation** | **Training** | **Validation** | **Training** | **Validation** | **Training** | **Validation** | **Training** | | **Validation** |
| Slope | 1.030 | 0.883 | 0.724 | 1.072 | 1.090 | 0.955 | 1.137 | 0.664 | 0.662 | | 0.545 |
| Intercept | 0.012 | -0.071 | -0.004 | -0.018 | 0.186 | 0.026 | 0.145 | 0.117 | -0.164 | | -0.118 |
| R-squared | 0.412 | 0.555 | 0.912 | 0.934 | 0.792 | 0.902 | 0.838 | 0.573 | 0.716 | | 0.719 |
| P-value | 0.062 | 0.034 | 0.000 | 0.000 | 0.001 | 0.000 | 0.000 | 0.018 | 0.002 | | 0.002 |
| Standard error | 0.461 | 0.321 | 0.085 | 0.108 | 0.197 | 0.111 | 0.177 | 0.217 | 0.148 | | 0.210 |
